# Systematic Distribution of Bioluminescence in Marine Animals: A Species-Level Inventory

**DOI:** 10.3390/life14040432

**Published:** 2024-03-24

**Authors:** Julien M. Claes, Steven H. D. Haddock, Constance Coubris, Jérôme Mallefet

**Affiliations:** 1 Marine Biology Laboratory, Earth and Life Institute, Université catholique de Louvain, 1348 Louvain-la-Neuve, Belgium; constance.coubris@uclouvain.be (C.C.); jerome.mallefet@uclouvain.be (J.M.); 2 Monterey Bay Aquarium Research Institute, 7700 Sandholdt Road, Moss Landing, CA 95039, USA

**Keywords:** bioluminescence, deep-sea, diversity, systematics, photophores, photocytes, marine luminescent animals

## Abstract

Bioluminescence is the production of visible light by an organism. This phenomenon is particularly widespread in marine animals, especially in the deep sea. While the luminescent status of numerous marine animals has been recently clarified thanks to advancements in deep-sea exploration technologies and phylogenetics, that of others has become more obscure due to dramatic changes in systematics (themselves triggered by molecular phylogenies). Here, we combined a comprehensive literature review with unpublished data to establish a catalogue of marine luminescent animals. Inventoried animals were identified to species level in over 97% of the cases and were associated with a score reflecting the robustness of their luminescence record. While luminescence capability has been established in 695 genera of marine animals, luminescence reports from 99 additional genera need further confirmation. Altogether, these luminescent and potentially luminescent genera encompass 9405 species, of which 2781 are luminescent, 136 are potentially luminescent (e.g., suggested luminescence in those species needs further confirmation), 99 are non-luminescent, and 6389 have an unknown luminescent status. Comparative analyses reveal new insights into the occurrence of luminescence among marine animal groups and highlight promising research areas. This work will provide a solid foundation for future studies related to the field of marine bioluminescence.

## 1. Introduction

Bioluminescence has fascinated humans and puzzled scientists since Antiquity [1,2]. Defined as the emission of visible light by an organism, this phenomenon is the by-product of an oxidation reaction [3]. Luminescent organisms harbor various photogenic structures ranging from simple cells (photocytes) to complex organs called photophores. Photophores typically combine photocytes with sophisticated optical elements (e.g., mirrors, light guides, lenses, filters, and shutters) modifying the physical characteristics (intensity, wavelength, and angular distribution) of the light emission [4,5]. In symbiotic photophores, these photocytes are replaced by luminescent bacteria [4]. Photogenic structures are controlled by diverse physiological ‘switches’ (e.g., neurotransmitters, hormones, and extraocular opsin-based feedback loops [4,6,7,8,9,10,11,12,13,14]) and produce diverse photic displays. These displays are classified into two broad categories based on the duration of the light emission: flashes (light emission ≤ 2 s) and glows (light emission > 2 s) [4].

Luminescence can be observed in most of the Earth’s ecological niches and displays a broad phylogenetic distribution, with a peak diversity in marine animals [15]. In the permanent darkness of the deep-sea biome, and especially in the shelter-less space of the twilight mesopelagic zone (layer ranging from 200 to 1000 m depth), representatives of most animal groups have indeed evolved an arsenal of light-generating adaptations for predator evasion, prey capture, and conspecific or host attraction [16,17]. Luminescent animals may be particularly abundant. In recent comprehensive studies involving in situ video recording, luminescence capability has been observed in 76% of macroscopic individuals from the water column (from surface to abyssal depths) and in up to 41% of macroscopic individuals from the benthos [18,19]. In addition, the luminescent bristlemouth fish *Cyclothone* is the world’s most abundant vertebrate genus and the species it contains are key components of marine ecosystems [20].

Establishing if an animal is luminescent can be challenging since bioluminescence is a low-intensity and ephemeral event that can only be reliably visible in live animals [21]. Moreover, individuals of a luminescent species might not appear to be luminous due to a series of factors including temporal (e.g., seasonal) changes in their luminescence capabilities [22,23,24], sexual dimorphism (e.g., there are no luminescent male anglerfishes) [25], ontogenetic variations (e.g., the visceral organ of *Thysanoteuthis rhombus* degenerates with age) [26] or dietary deficiencies in the components of the chemiluminescent reaction [27,28,29,30,31,32]. In addition to these situations sometimes leading to ‘false-negatives’, a series of factors can also lead to the identification of ‘false-positives’, e.g., non-luminescent animals incorrectly considered as luminescent. This latter case can lead to long-term problems, because once reported, it is very challenging to rule out the observation, especially if its exact context cannot be established [15,16]. Erroneous luminescence reports typically come from the contamination of dead or moribund animals with luminescent bacteria [33], gut contents, or adhered organisms, taxonomic mistakes [15], incorrect assumption that all species from a genus are luminous, confusion with other photic phenomena (e.g., iridescence or fluorescence) [16], or vivid imagination from researchers and naturalists establishing the photogenic character of non-luminous structures simply based on superficial inference rather than scientific evidence [17]. 

For many animal groups, the rise of molecular phylogenies enabled the evolutionary history of luminescence to be reconstructed, which was previously challenging because bioluminescence is a soft tissue-based process leaving no fossil clues [34,35,36,37,38,39]. Molecular phylogenetics also triggered a deep re-organization of marine animal systematics. Many species have been synonymized or moved to other genera (or even to higher taxonomic levels), subspecies have been erected as new species, subgenera have become new genera, and so on. All these elements have created additional confusion about the luminescence competence of a given species or genus. This, and the previously mentioned challenges in determining the luminescence status of a given species with certainty, might explain why—despite the paramount importance of animal luminescence for understanding marine ecology—there is currently no consolidated species-level list of luminescent marine animals available. Systematic studies on luminescence typically go no further than the genus-level [15,40] or they reach species-level but only for a relatively narrow taxonomic group [41,42,43,44,45,46]. 

Here, we have undertaken a comprehensive inventory of marine animal luminescence based on the review of over one thousand publications, close to 800 of which are featured in this publication. We also compiled erroneous reports of luminescence with associated references, effectively ‘busting the myth’, and established a list of non-luminous species from luminescent genera. We expect that this extensive listing will provide marine scientists and bioluminescence researchers with a strong foundation to perform research in their respective fields. 

## 2. Materials and Methods

### 2.1. Catalogue of Marine Luminescent Animals

A catalogue of marine luminescent animals was built leveraging prior exhaustive reviews (e.g., Herring’s luminescent genera catalogue from 1987 [15]) confirmed, corrected, and complemented with an exhaustive scientific literature screening spanning over one thousand publications, as well as unpublished data. Keywords such as ‘(bio)luminescence’, ‘light production’, ‘luminous’, ‘photophore’, ‘photocyte’, or ‘photogenic structure’ combined with taxonomic concepts (e.g., species, genera, families) mentioned in luminescence reviews led to the recovery of initial luminescence reports and their associated context (authority reporting the observation and its luminescence knowledge, year of the report, experimental procedure if any, and so on). This allowed us to identify the animal involved at the lowest possible taxonomic level (species in most of the cases) and to bring additional nuance to the typical ‘luminescent vs. non-luminescent’ binary classification, instead proposing a ‘luminescence scoring scale’ providing a finer view on the level of confidence in the luminescence competence of a given species (Table 1). 

Species or genera with a score of 4 or above were considered as luminescent, while species or genera with a score of 3 or below were considered as potentially luminescent, with additional research needed to confirm their luminescence competence. Genera received the highest score reported among the species they encompass.

In our review process, we also collected information regarding reports that have been conclusively considered as erroneous, and listed species from luminescent or potentially luminescent genera that are known to be non-luminescent. In addition, to provide an upper estimate of the number of luminescent marine animals, we counted the number of species from luminescent (scores 4–6) and potentially luminescent (scores 1–3) genera for which the luminescence competence has not been determined yet (unknown luminescent status). Finally, species not belonging to a luminescent or a potentially luminescent genus have been conservatively considered as non-luminescent in our calculation of the proportion of luminescent species across marine taxa. 

### 2.2. Taxonomy and Phylogenetic Tree Building

The taxonomy used in this study (from phylum to species level) followed The World Register of Marine Species (WoRMS), a comprehensive open-access inventory of all marine species’ names, which has increasingly become the authoritative reference for marine taxonomy [47,48]. Throughout our scientific literature review, however, we noticed a few deviations from the currently available version of the WoRMS database [49]. These deviations represent species recently described that are likely to be included as valid species in the WoRMS database soon, species for which synonymy or (non-)validity has been properly established, or species that need to be reallocated to another (often recently described) genus (deviations we followed as well as their associated references can be found in Table S1). 

A cladogram (constant branch length) of marine luminescent animals was built based on the topology from Lau and Oakley [50] (itself built based on recent molecular phylogenies of luminescent organisms, e.g., [37]), while, however, only including marine animal groups with luminescent representatives and placing Ctenophora as the most basal animal group, following the recent discovery by Schultz et al. [51]. Porifera has been included in the cladogram following the new description of a deep-sea luminescent sponge from the genus *Cladorhiza* [52]. Taxonomic groups were presented at the phylum level, with a higher taxonomic resolution (e.g., class, superorder, or family level) being provided for three phyla displaying a relatively higher specific diversity (20,000 species or more), e.g., Mollusca, Arthropoda, and Chordata.

The illustrated silhouettes of marine animal representatives were drawn using Adobe Illustrator (version 28.1).

## 3. Results

### 3.1. Catalogue of Marine Luminescent Animals

**Figure 1:**
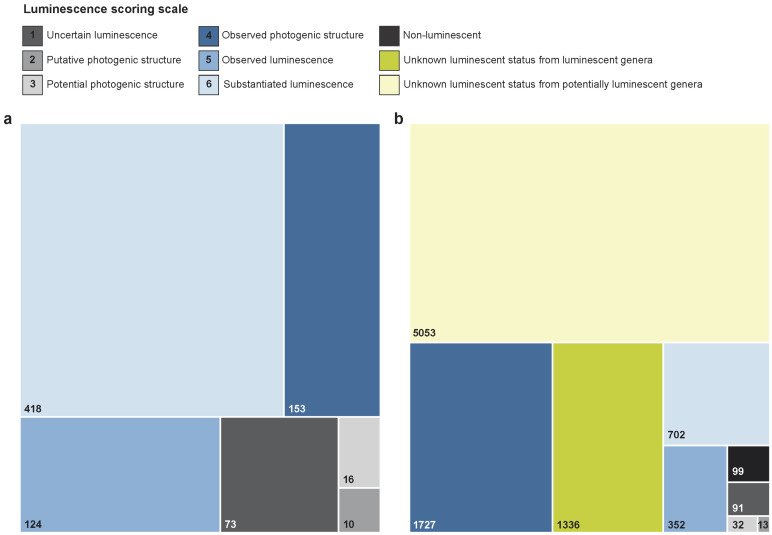
Proportional area chart showing the distribution of genera (**a**) and species (**b**) per luminescence score category (not including erroneous reports). The categories ‘Non luminescent’, ‘Unknown from luminescent genera’ and ‘Unknown from potentially luminescent genera’ are only relevant for panel b.

Our screening of the scientific literature, complemented by unpublished data, led to the record of 819 genera and 2951 species of marine animals from luminescence reports, of which only a tiny fraction—30 genera and 34 species—appears to be erroneous reports (Table S2). A luminescence score of 4 or above was attributed to most of the inventoried genera (87.53%) and species (95.34%), which we then considered as ‘luminescent’ in the rest of the article (Figure 1a). Luminescent and potentially luminescent (luminescence score of 3 or below) species (or higher taxonomic level such as genus, when no specific species has been identified) from valid reports are listed for each marine animal phylum in Table S3 (‘Catalogue of marine luminescent animals’), along with their respective taxonomic information and luminescence score (with associated luminescence report reference). Luminescent genera are mostly associated with a luminescence score of 6 (‘Substantiated luminescence’; 60.14% of valid reports) while luminescent species are mostly associated with a luminescence score of 4 (‘Photogenic structure’; 59.21% of valid reports). Luminescent and potentially luminescent genera encompass 9405 species, among which a small fraction (1.05% or 99 species) can be conclusively considered non-luminescent (Table S4) and about two-thirds (67.96%) have an unknown luminescent status (Figure 1b).

### 3.2. Generic Distribution of Luminescence across Marine Taxonomic Groups

**Figure 2:**
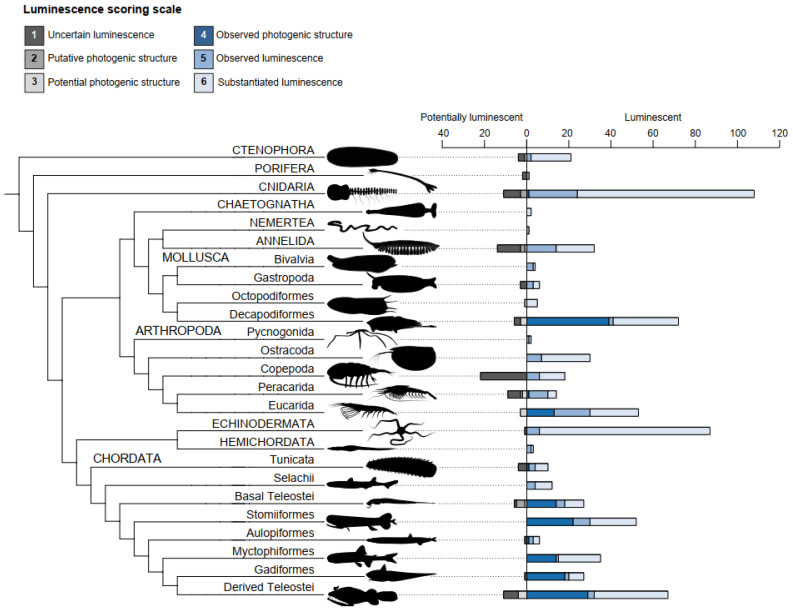
Generic distribution of luminescent marine animals. Capital letters are used for phylum-level groups while lower case labels are used for groups of lower taxonomic level. The bar chart next to the cladogram indicates the number of genera in each luminescence score category (not including erroneous reports). Luminescent genera encompass genera with at least one species displaying a luminescence score of 4 or above while potentially luminescent genera only have species with a luminescence score of 3 or below. ‘Basal Teleostei’ includes Alepocephaliformes, Anguilliformes, Argentiniformes, Clupeiformes, Nothacanthiformes and Saccopharyngiformes, while ‘Derived Teleostei’ includes Acanthuriformes (including Lophiiformes), Acropomatiformes, Batrachoidiformes, Beryciformes (including Trachicthyiformes), Eupercaria incertae sedis, Kurtiformes, Ophidiiformes and Scombriformes. Drawings by J.M. Claes.

The 695 luminescent genera of marine animals are distributed across numerous marine phyla (Figure 2), with about two-thirds of them found in Chordata (236 genera, including 226 genera of fishes), Arthropoda (117 genera, mostly of pelagic crustacea), and Cnidaria (108 genera). Mollusca (87 genera, including 72 genera of Decapodiformes, e.g., squids) and Echinodermata (87 genera) are other luminescent genera-rich phyla (each representing 12.52% of the total), while a very small number of luminescent genera are found in Porifera (one genus), Nemertea (one genus), Chaetognatha (two genera), and Hemichordata (three genera). In all phyla and most taxonomic groups, genera with luminescence score of 5 or above are more frequently observed; only in Decapodiformes, basal Teleostei, and Gadiformes are genera with luminescence score of 4 more frequent (54.17%, 51.85% and 66.67% of luminescent genera, respectively). Most of the 99 potentially luminescent genera are found in Arthropoda (34 genera, including 22 genera of Copepoda), Chordata (23 genera, including 11 genera of derived Teleostei), and Annelida (14 genera). 

### 3.3. Specific Distribution of Bioluminescence across Marine Taxonomic Groups

**Figure 3:**
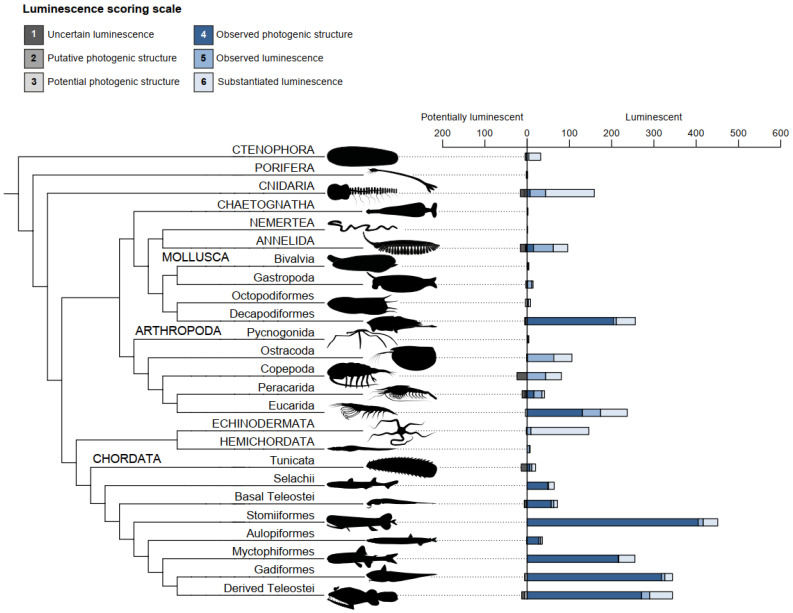
Specific distribution of marine luminescent animals. Capital letters are used for phylum-level groups while lowercase labels are used for groups of lower taxonomic level. The bar chart next to the cladogram indicates the number of species in each luminescence score category (not including erroneous reports). Luminescent species encompass species with luminescence scores ranging from 4 to 6 while potentially luminescent species encompass species with luminescence scores ranging from 1 to 3 while. ‘Basal Teleostei’ and ‘Derived Teleostei’ include the same orders as in Figure 2. Drawings by J.M. Claes.

Chordata (1586 luminescent species, including 451 luminescent species of Stomiiformes), Arthropoda (469 species, including 237 Eucarida species), and Mollusca (282 species, including 256 Decapodiformes species) account for 84.04% of the 2781 species of marine luminescent animals (Figure 3; Table S3). Cnidaria (159 species), Echinodermata (146 species), and Annelida (96 species) are other luminescent species-rich phyla, while the other phyla only contain a small fraction (1.55%) of the remaining luminescent species. The majority of the 136 potentially luminescent species are found in Chordata (41 species, including 14 species of Tunicata and 13 species of derived Teleostei), Arthropoda (41 species, including 24 species of Copepoda), Cnidaria (16 species), and Annelida (16 species). 

**Figure 4:**
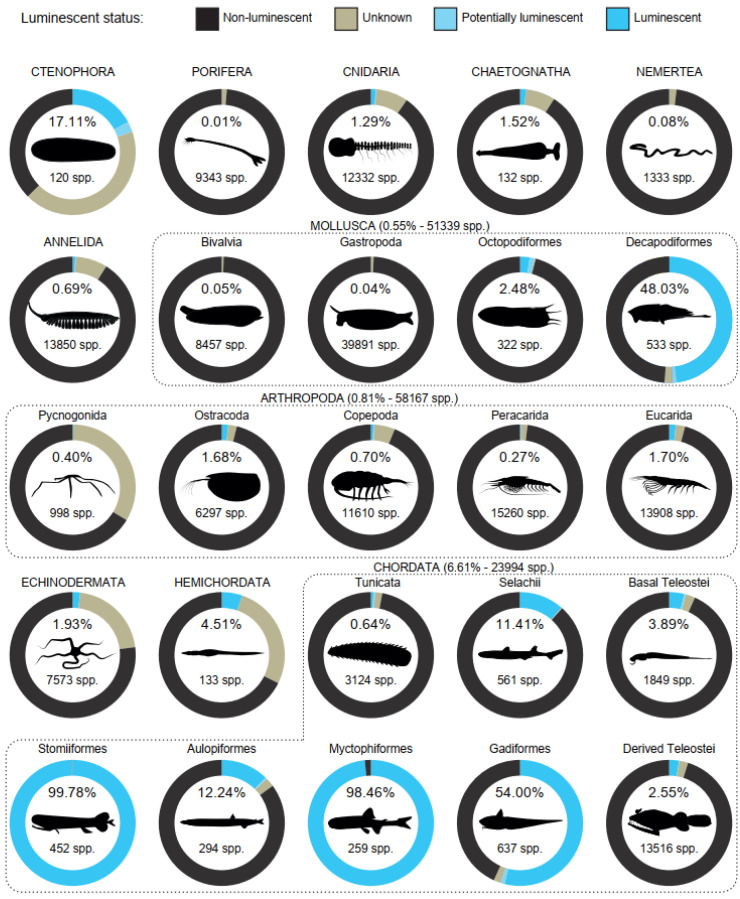
Proportion of luminescent species across marine phyla. Percentages at the top of each circle indicate the proportion of luminescent species (e.g., species with a luminescence score of 4 or above) among the total number of valid species in the corresponding taxonomic grouping (indicated at the bottom of the circle). Capital letters are used for phylum-level groups while minuscule letters are used for groups of lower taxonomic level. ‘Basal Teleostei’ includes Albuliformes, Elopiformes, Gonorhynchiformes, Osmeriformes Salmoniformes, and Siluriformes, in addition to the orders mentioned in Figure 2. ‘Derived Teleostei’ includes Anabantiformes, Atheriniformes, Beloniformes, Blenniformes, Callionymiformes, Carangiformes, Centrarchiformes, Cetomimiformes, Cichliformes, Cyprinodontiformes, Dactylopteriformes, Gobiesociformes, Holocentriformes, Labriformes, Mugiliformes, Mulliformes, Pleuronectiformes, Scorpaeniformes, Synbranchiformes, Syngnathiformes, Tetraodontiformes, and Uranoscopiformes in addition to the orders mentioned in Figure 2. Drawings by J.M. Claes.

Luminescent species represent a small fraction (<5%) of the total number of valid species from most invertebrate groups, except in Decapodiformes and Ctenophora, where the number of luminescent species reaches 48.03% and 17.11%, respectively (Figure 4). Overall, marine Chordata (including marine Tetrapoda and Cephalochordata) display a proportion of luminescent species of 6.61% and contains a series of taxonomic groupings with higher luminescent species richness (e.g., Selachii and Aulopiformes), or where luminescent species prevail (Gadiformes) or are virtually ubiquitous (Stomiiformes and Myctophiformes). Species with unknown luminescent status from luminescent and potentially luminescent genera represent over 5.00% of some of the taxonomic groups such as Ctenophora (42.78%), Pycnogonida (33.47%), Hemichordata (27.07%), Echinodermata (21.02%), Cnidaria (8.19%), Annelida (8.15%), and Chaetognatha (7.58%). 

The 6392 species from luminescent and potentially luminescent genera with unknown luminescent status are mostly found in Arthropoda (1655 species, including 617 Copepoda species and 334 Pycnogonida species), Echinodermata (1592 species), Annelida (1129 species), and Cnidaria (1009 species).

All invertebrate taxonomic groups (except Cephalopoda) have a proportion of species with unknown luminescent status higher than 50% (Table 2). The 99 non-luminescent species from luminescent or potentially luminescent genera were found in Echinodermata (fifty-five species), Chordata (thirty-one species), Cnidaria (seven species), Mollusca (five species) and Annelida (one species).

## 4. Discussion

The establishment of an exhaustive listing of luminescent marine animals has long been considered a virtually impossible task due to, on the one hand, how abundant the scientific literature treating the topic has been over the last two centuries, and, on the other hand, the incomplete or incorrect character of many of these luminescence reports [15,16]. While the exhaustiveness of our catalogue cannot be assessed (we included all the information we found), the number of missed reports (if any) is likely to be small. Indeed, our comprehensive literature review involved the screening of over one thousand publications and allowed us to recover species-level luminescence reports for a high fraction (>97%, excluding genera for which the absence of species-level report is certain) of marine animal genera seen or suspected to produce light in authoritative luminescence books [53,54] and luminescence reviews covering various taxonomic groups from luminescent species-rich phyla such as Cnidaria [55], Annelida [43,56], Mollusca [45], Arthropoda [33,39,57,58,59,60,61], Echinodermata [44,46,62], and Chordata [40,42,63]. This and the significant amount of unpublished (or about-to-be-published [55]) luminescence information (59 species and 27 genera for which the luminescence competence is reported here for the first time) encompassed by our catalogue, position this paper as—to our knowledge—the most comprehensive listing of marine luminescent animals ever published. The validity of each species included herein has been checked using WoRMS (complemented with taxonomic information from recent publications), widely recognized as the gold standard in marine taxonomy [47]. We expect the taxonomic information provided in our file (e.g., phylum, class, order, family, genus, species, and authority) to ensure the resilience of each record to future taxonomic changes. Finally, our luminescence scoring scale provides a new way of defining the luminescence capabilities of a given species, departing from the typical luminescent/non-luminescent binary approach. Not only does it allow for a more informative and conservative assessment of the luminescence status of a given species, but it also highlights areas for future research, e.g., to confirm the luminescent status of some species. While we cannot rule out some judgment calls between some categories (e.g., typically between luminescence scores of 2 and 3), the scoring of the reports was nevertheless carried out using a rigorous approach based on clear criteria and always opting for a conservative approach (e.g., in the case of doubt, the lowest luminescence score was selected). 

With 695 luminescent genera of marine animals, our catalogue is ~40% larger than Herring’s authoritative listing from 1987 [15]. Combined with luminescent records from non-marine Annelida (~35 species from 16 genera [56,64,65]), Mollusca (six species from three genera [66]) and Arthropoda (3000 species from ~240 genera [15,67,68,69,70,71,72,73,74,75,76,77]), Fungi (~100 species from 14 genera [78,79,80]), Protists (~100 species from 27 luminescent genera [41,81]) and bacteria (forty-four species from six genera [82,83,84,85]), our listing amounts to over 6000 luminescent species from about 1000 genera, which represents at least a 20% increase compared to previous estimates [3,17]. Two driving forces have fueled this augmentation: an ‘organic’ growth linked to taxonomic changes triggered by the rise of molecular phylogenies (e.g., elevation of subgenera to genera level, splitting of genera to avoid paraphyly, and so on), and a ‘methodological’ growth linked to the discovery of new luminescent species (or to the discovery of the luminescence competence of species discovered before). In the coming decades, however, we expect the latter to be the main vector of luminescent animal genera increase since (i) many taxonomic groups have now undergone molecular-enabled taxonomic changes and, hence, might have reached relative taxonomic stability, and (ii) the development of deep-sea exploration technologies and light measurement techniques will likely fuel the detection of new luminescent animals, facilitating the in situ observation of luminescent behaviors and the collection of animals in good physiological conditions for laboratory experimentation [18,19,86,87,88,89]. 

Strikingly, while marine animals encompass about 70% of luminescent genera known to science, they represent less than half of the world’s known luminescent organisms. This discrepancy between generic and specific diversity might reflect the lower accessibility of field observation and laboratory experiments to marine animals (especially those inhabiting the deep-sea). The large number of species with an unknown luminescent status found in both luminescent (~65% of species) and potentially luminescent (~90% of species) genera from our catalogue supports this idea. We believe that many of these species are in fact endowed with a luminescent capability since, as highlighted by the present work, non-luminous species are rare among marine luminescent genera (e.g., they occur in <5% of them). If true, this would double the number of luminescent taxa recorded. Two bioluminescence characteristics might explain the infrequent occurrence of non-luminous representatives among luminescent genera. The first is the high adaptive value of luminescence [16,17], which makes it a resilient trait persisting during long evolutionary times, hence leading to relatively rare ‘luminescence loss’ events. The second is the accelerated speciation rate of luminescent species [90,91,92], which quickly generates a generic-level genetic distance from their non-luminescent counterparts, making non-luminescent species from a luminescent genus only a transient situation. This would be particularly true for species like *Cylothone obscura* (the only non-luminescent Stomiidae), which lost the photophores involved in camouflage by counterillumination following the adoption of a bathypelagic lifestyle (where counterillumination is useless [93,94,95]) and hence have a physical barrier separating them from congeneric species. 

As already observed by Harvey [53], the distribution of luminescence across marine animal taxa is far from homogeneous: some groups are luminescent taxa-rich (e.g., fishes and Decapodiformes) while others are luminescent taxa-poor (e.g., Porifera and Nemertea) or completely lack luminescent representatives (e.g., 20 phyla including marine species-rich phyla such Platyhelminthes, Bryozoa, and Nematoda). In relative terms, luminescence clearly stands out as a rare phenomenon across marine taxa since confirmed luminescent species account for only 1.32% of marine animals currently considered valid in WoRMS (*circa* 210,000 species including all phyla) [49]). In addition, five phyla show less than 1% of luminous species. However, a similarly small fraction of marine animal species has been thoroughly tested under appropriate conditions, so fundamental discoveries will continue to be made.

Our results confirmed the exceptional occurrence of luminescence in Decapodiformes, which is ~100 times higher than that observed in the phylum Mollusca. Yet, they challenge the view that virtually all Ctenophora are luminous, since benthic Platyctenes—which currently lack any luminescent reports—have had high taxonomic proliferation (52 species in WoRMS), although we did find this phylum to have the highest relative richness in luminescent species (17.00%). Finally, our work allows us, for the first time, to properly compare the luminescence occurrence in bony and cartilaginous fishes, e.g., using robust taxonomy and controlling for environment (marine) and taxonomic level (class); as a result, luminescent species represent a higher proportion of Teleostei (8.50% of 17,671 species) than Elasmobranchii (5.20% of 1255 species). Determining if such results reflect uneven information from heterogeneous research efforts or rather highlight fundamental differences in functional ecology across groups remains a challenging scientific question [17]. This overview, however, reveals some intriguing broad trends. Luminescent taxon-rich groups appear to contain a high fraction of relatively large pelagic species bearing counterilluminating photophores. These photophores are easy to spot using the naked eye, even by a luminescence non-specialist since (i) they are often relatively large with a well-identifiable structure adapted to their role (e.g., they typically have a pigmented sheath with accessory optical structures such as lenses and/or filters [96]), and (ii) they produce a long lasting-light display (typically ‘glows’, which last >2 s) even in moribund specimens, which facilitate field and laboratory observations [97]. On the other hand, many of the luminescent taxon-poor groups contain benthic species with virtually invisible photogenic structures (e.g., isolated photocytes or internal secretory organs) involved in short light displays (e.g., typically ‘flashes’, which last ≤2 s) upon predator stimulation [16,98,99,100,101,102,103]. This strongly suggests the underrepresentation of luminescent taxa in some taxonomic groups to reflect (at least partly) an experimental bias rather than a difference in functional ecology. The higher number of species with unknown luminescent status (from both luminescent and potentially luminescent genera) and luminescence score 6 (‘Substantiated luminescence’) reports in these groups support this hypothesis. Benthic deep-sea invertebrates, therefore, likely represent an unexplored potential area for discovering new luminescent animals. A recent increase in experimental focuses on deep-sea Cnidaria and Echinodermata over the last decade has yielded a fair amount of new luminescent genera and species [44,46,55,104,105,106], and we believe this trend will continue. In addition, the improvement in deep-sea collection techniques will also likely lead to luminous discoveries in groups such as Chaetognatha, which have historically been challenging to study in good physiological conditions [107,108]. 

## 5. Conclusions

Our catalogue, based on the latest taxonomic information and associated with an innovative luminescence scoring scale, represents the most comprehensive and informative listing of luminescent marine animals ever published. As such, it not only provides updates on key ecological numbers (e.g., it shows that the world’s luminescent organisms now encompass ~1000 genera, which represents a significant increase compared to previous estimates), it also consists in a robust foundational tool for studies in or related to the field of bioluminescence and marine biology. We hope this work will inspire future luminescence research dedicated to taxonomic groups whose luminous properties have, to this day, remained obscure.

## Figures and Tables

**Table 1:** Luminescence scoring scale.

Score	Name	Description
1	Uncertain luminescence	Report previously considered as dubious by a luminescence authority (e.g., Peter J. Herring), or to genera reported as luminescent but currently lacking a species-level report following the reallocation of luminescent species to other genera.
2	Putative photogenic structure	Report featuring an anatomical element considered as photogenic while not sharing similarities with structures confirmed as photogenic in species from the same order.
3	Potential photogenic structure	Report featuring an anatomical element sharing similarities (e.g., similar shape or fluorescence pattern) with structures confirmed as photogenic in species from the same order.
4	Photogenic structure	Report featuring an anatomical element displaying the typical morphology of structures confirmed as photogenic in species from the same family (e.g., counterilluminating photophores of crustaceans, cephalopods, and fishes).
5	Observed luminescence	Report featuring luminescence observations made in relevant conditions and reported by credible scientific authorities.
6	Substantiated luminescence	Report for which irrefutable scientific evidence (e.g., via luminometry, spectrophotometry, biochemistry, long exposure photography or light-intensified video recordings) of the luminescence competence of the species has been provided.

**Table 2:** Distribution of species with undetermined luminescent status from luminescent and potentially luminescent genera across marine animal taxonomic groupings.

	Number of Species with Undetermined Status		
Taxonomic Group	From Luminescent Genera	From Potentially Luminescent Genera	Proportion with Undetermined Status (% ^1,2^)
CTENOPHORA *	54	26	46.15–68.38
PORIFERA *	0	118	0.00–97.52
CNIDARIA **	898	111	75.40–84.72
CHAETOGNATHA **	10	0	83.33
NEMERTEA **	24	0	96.00
ANNELIDA **	944	185	76.01–90.90
MOLLUSCA			
Bivalvia **	38	0	90.48
Gastropoda **	238	31	82.93–93.73
Octopodiformes	1	0	7.69
Decapodiformes	0	11	0.0–3.97
ARTHROPODA			
Pycnogonida **	334	0	98.82
Ostracoda **	139	1	56.28–56.68
Copepoda *	200	414	27.82–85.40
Peracarida *	58	178	21.16–81.91
Eucarida **	303	21	53.63–57.35
ECHINODERMATA **	1589	3	88.52–88.69
HEMICHORDATA **	36	0	83.33
CHORDATA			
Tunicata **	55	2	56.70–58.76
Selachii	1	0	1.54
Basal Teleostei	22	23	15.94–32.61
Stomiiformes	0	0	0.00
Aulopiformes	7	0	15.91
Myctophiformes	0	0	0.00
Gadiformes	11	1	3.02–3.30
Derived Teleostei	88	211	13.25–45.03

^1^ For each taxonomic grouping, the smallest value refers to species from luminescent genera (luminescence scores from 4 to 6) with undetermined luminescent status while the highest value refers to species from both luminescent and potentially luminescent (luminescence scores from 1 to 3) genera with undetermined luminescent status. ^2^ Using the total number of species from both luminescent and potentially luminescent genera as a common denominator. * Taxonomic group with a proportion of species with undetermined luminescent status from luminescent and potentially luminescent genera > 50%; ** Taxonomic group with a proportion of species with undetermined luminescent status from luminescent genera > 50%. Capital letters are used for phylum-level groups while lowercase labels are used for groups of lower taxonomic level. Basal Teleostei’ and ‘Derived Teleostei’ include the same orders as in Figure 2.

## Data Availability

All data supporting the article are included in the Supplementary Materials section.

## Supplementary Materials

The following supporting information can be downloaded at: https://www.mdpi.com/article/10.3390/life14040432/s1, Table S1: List of followed deviations from WoRMS (World Register of Marine Species) database (https://www.marinespecies.org/; accessed on 15 January 2024); Table S2: Erroneous luminescence reports; Table S3: Catalogue of marine luminescent animals; Table S4: List of non-luminescent species from luminescent genera.
