# Supplementary material for: Systematic Distribution of Bioluminescence in Marine Animals: A Species-Level Inventory
Source: Life (Basel). 2024 Mar 24;14(4):432. doi: 10.3390/life14040432 (PMC11051050; doi:10.3390/life14040432)
Supplement: Supplementary file 1 [file life-14-00432-s001.zip › Supplementary references.pdf]

Supplementary references

1. Moore J.M., Moore, 2019. Phylogeny, Systematics, and evolution of functional morphology in Chaetopteridae (Annelida). PhD thesis, University of Florida, Gainesville, Florida, USA, 24 October 2019.
2. Dolganov, V.N. Description of new species of sharks of the family Squalidae (Squaliformes) from the north-western part of the Pacific Ocean with remarks of validity of *Etmopterus frontimaculatus*. *Zool. Zhurnal* **1986** 65(1), 149–153
3. Ng, S-L; Liu, K.-M.; Joung, S.L. Description of a new lanternshark species from the South China Sea, with additional description of *Etmopterus sheikoi* from Taiwanese waters (Squaliformes: Etmopteridae). *Raffles Bull. Zool*. **2024** 72, 26–41. (doi:10.26107/RBZ-2024-0002)
4. Luna, A.; Rocha, F.; Perales-Raya, C. A review of cephalopods (Phylum: Mollusca) of the Canary Current Large Marine Ecosystem (Central-East Atlantic, African coast). *J. Mar. Biol. Assoc. U. K.* **2021** 101(1), 1-25. (doi:10.1017/S0025315420001356)
5. Evans, A.B.; Bolstad, K.S.R. Diversity of the squid genus *Leachia* (Oegopsida: Cranchiidae) in the Pacific Ocean. *Mar. Biol.* **2023** 170(6), 72. (doi:10.1007/s00227-023-04215-2)
6. Braid, H.E.; Bolstad, K.S.R. Systematics of the Mastigoteuthidae Verrill, 1881 (Cephalopoda: Oegopsida) from New Zealand waters. *N. Z. J. Zool.* **2015** 42(3), 187-256. (doi:10.1080/03014223.2015.1063516)
7. Kelly, J.T. Systematics of the Octopoteuthidae Berry, 1912 (Cephalopoda: Oegopsida). PhD thesis, Auckland University of Technology, New Zealand, 30 October 2019.
8. Qiu, D.; Liu, B.; Guo, Y.; Lakmini, W.A.S.M.; Tan, Y.; Li, G.; Ke, Z., Li, K.;Huang, L. *Vampyroteuthis southchinaseais* sp. nov., a second Recent widely distributed species of Vampyromorpha (Cephalopoda, Coleoidea). *bioRxiv* **2023** 14 Feb. (doi:10.1101/2023.02.13.526086)
9. Fricke, R.; Durville, P. *Chlorophthalmus vulcanus*, a new species of greeneye from La Réunion, southwestern Indian Ocean (Teleostei: Chlorophthalmidae). *J. Fish Taxonomy* **2020** 17: 1-11.
10. Cohen, D.M.; Inada, T.; Iwamoto, T.; Scialabba, N. Gadiform Fishes of the World (Order Gadiformes; volume 10). An Annotated and Illustrated Catalogue of Cods, Hakes, Grenadiers and Other Gadiform Fishes Known to Date; Food and Agriculture Organization of the United Nations: Rome, Italy, 1990, pp. 139-140.
11. Nakayama, N. Grenadiers (Teleostei: Gadiformes: Macrouridae) of Japan and adjacent waters, a taxonomic monograph. *Megataxa* **2020** 3(1), 1-383. (doi:10.11646/MEGATAXA.3.1.1)
12. Prokofiev, A.M. A new species of the genus *Coelorinchus f*rom the Northwestern and Hawaiian submarine ridges (Pacific Ocean) (Teleostei, Gadiformes, Macrouridae). *Amur Zool. J.* **2020** 12(3), 299-310 (doi:10.15468/39omei)
13. Baldwin, Z.H.; Sparks, J.S. A new species of *Secutor* (Teleostei: Leiognathidae) from the western Indian Ocean. *Zootaxa* **2011** 2998(1), 39-47. (doi:10.11646/zootaxa.2998.1.3)
14. Miki, R.; Murase, A.; Wada, M. First record of the ponyfish *Deveximentum interruptum* (Teleostei: Leiognathidae) from Miyazaki Prefecture, Kyushu, Japan. *Biogeography* **2017** 19, 127-132 (doi:10.11358/biogeo.19.127)
15. Prokofiev, A.M.; Emelyanova, O.R.; Orlov, A.M.; Orlova, S.Y. A New Species of *Diaphus* Associated with Seamounts of the Emperor Chain, North-Western Pacific Ocean (Teleostei: Myctophiformes: Myctophidae). *J. Mar. Sci. Eng.* **2022** 10(1), 65. (doi:10.3390/jmse10010065)
16. Gibbs, R.H.J.; Clarke, T.A.; Gomon, J.R. Taxonomy and distribution of the stomioid fish genus *Eustomias* (Melanostomiidae), I: Subgenus *Nominostomias. Smithson. Contrib. Zool.* **1983** 380, 1–139.
17. Sparks, J.S. A new species of ponyfish (Teleostei:Leiognathidae:*Photoplagios*) from Madagascar, with a phylogeny for *Photoplagios* and comments on the status of *Equula lineolata* Valenciennes. *Am. Mus. Novit.* **2006** 3526, 1-20. (doi10.1206/0003-0082(2006)3526[1:ANSOPT]2.0.CO;2)
18. Koeda, K.; Ho, H.C. Review of the genus *Eustomias* (Stomiiformes: Stomiidae: Melanostomiinae) of Taiwan, with descriptions of three new species. *Zootaxa* **2019** *4702*(1), 94-106. (doi:10.11646/zootaxa.4702.1.14)
19. Pietsch, T.W.*Oceanic anglerfishes: extraordinary diversity in the deep sea*. Univ of California Press. Berkeley and Los Angeles, USA, 2009., pp. 1-576.
20. Schwarzhans, W. Head and otolith morphology of the genera *Hymenocephalus*, *Hymenogadus* and *Spicomacrurus* (Macrouridae), with the description of three new species. *Zootaxa* 2014 *3888*(1), 1-73. (doi:10.11646/zootaxa.3888.1.1)
21. Gon, O. Revision of the cardinalfish subgenus Jaydia (Perciformes, Apogonidae, Apogon). *Trans. R. Soc. S. Afr.* **1997** 51(1), 147-194. (doi:10.1080/00359199609520605)
22. Fraser, T.H. A new species of Apogon (Perciformes: Apogonidae) from the Saya de Malha Bank, Indian Ocean, with redescriptions of *Apogon regani* Whitley, 1951, *A. gardineri* Regan, 1908, and *A. heraldi* (Herre, 1943). *Proc. Biol. Soc. Wash.* **2000** 113 (1), 249-263.

23. Mabuchi, K.; Fraser, T.H.; Song, H.; Azuma, Y.; Nishida, M. Revision of the systematics of the cardinalfishes (Percomorpha: Apogonidae) based on molecular analyses and comparative reevaluation of morphological characters. *Zootaxa* **2014** 3846(2), 151-203. (doi:10.11646/zootaxa.3846.2.1)
24. Sparks, J.S.; Chakrabarty, P. Description of a new genus of ponyfishes (Teleostei: Leiognathidae), with a review of the current generic-level composition of the family. *Zootaxa* **2015** *3947*(2), 181-190. (doi:10.11646/zootaxa.3947.2.2)
25. Villarins, B.T.; Fischer, L.G.; Prokofiev, A.M.; Mincarone, M.M. A New Species of the Dragonfish Genus *Melanostomias* (Stomiidae: Melanostomiinae) from the Western Tropical Atlantic. *Ichthyol. Herpetol.* **2023** 111(2), 254-263. (doi:10.1643/i2022082)
26. Sazonov, Y. Three rare species of slickheads (Alepocephalidae), found for the first time in the Indian Ocean, with remarks about the status of *Microphotolepis schmidti*. *Oceanogr. Lit. Rev.* **1996** 6(43), 588.
27. Su, Y.; Lin, H.-C.; Ho, H.-C. A new cryptic species of the pineapple fish genus *Monocentris* (Family Monocentridae) from the western Pacific Ocean, with redescription of *M. japonica (*Houttuyn, 1782). *Zootaxa* **2022** 5189(1), 180-203. (doi:10.11646/zootaxa.5189.1.18)
28. Martin, R.P.; Olson, E.E.; Girard, M.G.; Smith, W.L.; Davis, M.P. Light in the darkness: new perspective on lanternfish relationships and classification using genomic and morphological data. *Mol. Phylogenetics Evol.* **2018** *121*, 71-85.(doi:10.1016/j.ympev.2017.12.029)
29. Paulin, C.D. Review of the morid genera *Gadella*, *Physiculus*, and *Salilota* (Teleostei: Gadiformes) with descriptions of seven new species. *N. Z. J. Zool.* **1989** 16(1), 93-133.
30. Babu, K.I.; Ho, H.C.; Mariyambi, P.C.; Sureshkumar, S. Two new species of the codling fish genus *Physiculus* from Lakshadweep, India (Gadiformes: Moridae). *Zootaxa* **2022** 5104(1), 111-124. (doi:10.11646/zootaxa.5104.1.6)
31. Duchatelet, L.; Moris, V.C.; Tomita, T.; Mahillon, J.; Sato, K.; Behets, C.; Mallefet, J. The megamouth shark, *Megachasma pelagios*, is not a luminous species. *PLoS One* **2020** 15(11), e0242196. (doi:10.1371/journal.pone.0242196)
32. Bullard, S.A. In Memoriam: George William Benz (1 January 1954–9 February 2015). *Acta Ichthyol. Piscat.* **2016** 46(2), 141-162. (doi:10.3750/AIP2016.46.2.12)
33. Hubbs, C.L.; Iwai, T.; Matsubara, K. (1967). External and internal characters, horizontal and vertical distributions, luminescence, and food of the dwarf pelagic shark, *Euprotomicrus bispinatus*. *Bull. Scripps Inst. Oceanogr. Univ. Calif.* **1967** 10, 1-81 (https://escholarship.org/uc/item/0868j08s)
34. de Carvalho, M.R. A synopsis of the deep-sea genus *Benthobatis* Alcock, with a redescription of the type species *Benthobatis moresbyi* Alcock, 1898 (Chondrichthyes, Torpediniformes, Narcinidae). In *Proceedings of the 5th Indo-Pacific Fish Conference*, Nouméa, New Caledonia, 3–8 November 1997
35. Munk, O. The eyes of *Ipnops murrayi* Gunther 1887. *Galathea Rep.* **1959** 3, 79-87.
36. Herring, P.J.; Morin, J.G. Bioluminescence in fishes. In *Bioluminescence in Action*, 1st ed.; Herring, P.J., ed.; Academic Press: London, UK, 1978; pp. 273-329.
37. Munk, O. On the eye and the so-called preorbital light organ of the isospondylous deep-sea fish, *Bathylaco nigricans* Goode and Bean, 1896. *Galathea Rep.* **1968** 9, 211-218.
38. Bertelsen, E.; Krefft, G.; Marshall, N.B. The fishes of the family Notosudidae. *Dana Rep.* **1976** 86, 1–114.
39. Robins, C.R. Additional comments on the structure and relationships of the mirapinniform fish family Kasidoroidae. *Bull. Mar. Sci.* **1966** 16(4), 696-701.
40. Haneda, Y. *Harpodon nehereus*, a nonluminous fish. *Pac. Sci.* **1950** 4, 135-138.
41. Paxton, J.R.; Trnski, T.; Johnson, G.D. Centomimidae. In *The living marine resources of the Eastern Central Atlantic. Volume 3: Bony fishes part 1 (Elopiformes to Scorpaeniformes)*, 1st ed.; Carpenter, K.E.; De Angelis, N.; Eds.; Food and Agriculture Organization: Rome, Italy, 2016; pp. 2174–2182.
42. Crane, J.M. Bioluminescence in the batfish *Dibranchus atlanticus*. *Copeia* **1968** 1968(2), 410-411.
43. Bowman, T.; Phillips, F. Bioluminiscence in the freshwater amphipod, *Hyalella azteca*, caused by pathogenic bacteria. *Proc. Biol. Soc. Wash.* **1984** 97, 526–528.
44. Dougherty, L.F.; Johnsen, S.; Caldwell, R.L.; Marshall, N.J. A dynamic broadband reflector built from microscopic silica spheres in the 'disco'clam *Ctenoides ales*. *J. R. Soc. Interface* **2014** 11(98), 20140407. (doi: 10.1098/rsif.2014.0407)
45. Cohen, D.M. Notes on the morid fish genera *Lotella* and *Physiculus* in Japanese waters. *Jpn. J. Ichthyol.* **1979** 26(3), 225-230. (doi:10.11369/jji1950.26.225)
46. Inman, O.L. A pathogenic luminescent bacterium. *Biol. Bull.* **1927** 53, 197–200. (doi:10.2307/1537000)
47. Bousfield, E.L.; Klawe, W.L. *Orchestoidea gracilis*, a New Beach Hopper (Amphipoda: Talitridae) from Lower California, Mexico, with Remarks on Its Luminescence. *Bull. South. Calif. Acad. Sci.* **1963** 62, 1–8. (doi:10.3160/0038-3872-62.1.1)
48. Giard, A. Sur l'infection phosphorescente de talitres et autres crustacés. *C. R. Acad. Sci. Paris* **1889** 109, 503–506.

49. Yasaki, Y. Bacteriologic Studies on Bioluminescence: 1. Cause of Luminescence in the Fresh Water Shrimp, *Xiphocaridina Compressa* (De Haan). *J. Infect. Dis.* **1927** 404-407.
50. Lochhead, J.H. On the distribution of a marine cladoceran, *Penilia avirostris* Dana (Crustacea, Branchiopoda), with a note on its reported bioluminescence. *Biol. Bull.* **1954** 107(1), 92-105. (doi:10.2307/1538633)
51. Haddock, S.H.; Case, J.F. Bioluminescence spectra of shallow and deep-sea gelatinous zooplankton: ctenophores, medusae and siphonophores. *Mar. Biol.* **1999** 133(3), 571-582. (doi:10.1007/s002270050497)
52. Gershwin, L.A.; Zeidler, W.; Davie, P.J. Ctenophora of Australia. *Mem. Queensl. Mus.* **2010** 54(3), 1-45.
53. Cronin, H.A.; Cohen, J.H.; Berge, J.; Johnsen, G.; Moline, M.A. Bioluminescence as an ecological factor during high Arctic polar night. *Sci. Rep.* **2016** 6(1), 36374. (doi:10.1038/srep36374)
54. Dawydoff, C. Contribution à la connaissance des cténophores pélagiques des eaux de l'Indochine. *Bull. Biol. Fr. Belg.* **1946** 80 (2), 113-170.
55. Haddock, S.H.; Case, J.F. Not all ctenophores are bioluminescent: *Pleurobrachia*. *Biol. Bull.* **1995** 189(3), 356-362. (doi:10.2307/1542153)
56. Morin, J.G.; Hastings, J.W. Biochemistry of the bioluminescence of colonial hydroids and other coelenterates. *J. Cell. Physiol.* **1971** 77(3), 305-311. (doi:10.1002/jcp.1040770304)
57. Matsumoto, G.I.; Harbison, G.R. *In situ* observations of foraging, feeding, and escape behavior in three orders of oceanic ctenophores: Lobata, Cestida, and Beroida. *Mar. Biol.* **1993** 117, 279-287. (doi:10.1007/BF00345673)
58. Okada, Y.K. Light localization in Ctenophores. *Science* **1926** 63(1627), 262-262. (doi:10.1126/science.63.1627.262)
59. Harvey, E.N. Studies on Bioluminescence. XIII. Luminescence in the Coelenterates. *Biol. Bull.* **1921** 41(5), 220-287. (doi:10.2307/1536528)
60. Wiens, M.; Wang, X.; Unger, A.; Schröder, H.C.; Grebenjuk, V.A.; Pisignano, D.; Jochum, K.P.; Müller, W.E. Flashing light signaling circuit in sponges: endogenous light generation after tissue ablation in *Suberites domuncula*. *J. Cell. Biochem.* **2010** 111(6), 1377-1389. (doi:10.1002/jcb.22866)
61. Tur, J.M. Redescription and biological aspects of *Hormathia alba* (Andres, 1881), a luminescent sea anemone (Anthozoa, Actiniaria). *Helgoländer Meeresunters.* **1993** 47, 213-219. (doi:10.1007/BF02430359)
62. Herring, P.J. The spectral characteristics of luminous marine organisms. *Proc. R. Soc. B.* **1983** 220(1219), 183-217. (doi:10.1098/rspb.1983.0095)
63. Herring, P.J. Bioluminescence in invertebrates other than insects. In *Bioluminescence in Action*, 1st ed.; Herring, P.J., ed.; Academic Press: London, UK, 1978; pp. 199-240.
64. Herring, P.J. Observations on bioluminescence in some deep-water anthozoans. In *Proceedings of the Fifth International Conference on Coelenterate Biology*, Southampton, UK, 10–14 July 1989.
65. Widder, E.A.; Latz, M.I.; Case, J.F. Marine bioluminescence spectra measured with an optical multichannel detection system. *Biol. Bull.* **1983** 165(3), 791-810. (doi:10.2307/1541479)
66. Williams, G.C. The Pennatulacea of Southern Africa. *Ann. S. Afr. Mus.* **1990** 99, 31-119.
67. Muzik, K. A bioluminescent gorgonian, *Lepidisis olapa*, new species (Coelenterata: Octocorallia), from Hawaii. *Bull. Mar. Sci.* **1978** 28(4), 735-741.
68. Ramesh, C.; Meyer-Rochow, V.B. Bioluminescence in aquatic and terrestrial organisms elicited through various kinds of stimulation. *Aquat. Ecol.* **2021** 55(3), 737-764. (doi:10.1007/s10452-021-09875-0)
69. Williams, G.C. First record of a bioluminescent soft coral: description of a disjunct population of *Eleutherobia grayi* (Thomson and Dean, 1931) from the Solomon Islands, with a review of bioluminescence in the Octocorallia. *Proc. Calif. Acad. Sci.* **2001** 52, 209–225
70. Francis, W.R.; de Vilar, A.S. Bioluminescence and fluorescence of three sea pens in the north-west Mediterranean Sea. *bioRxiv.* **2020** 1-15. (doi:10.1101/2020.12.08.416396)
71. Wampler, J.E.; Karkhanis, Y.D.; Morin, J.G.; Cormier, M.J. Similarities in the bioluminescence from the Pennatulacea. *Biochim. Biophys. Acta Bioenerg.* **1973** 314(1), 104-109. (doi:10.1016/0005-2728(73)90068-6)
72. Geetha, S.; Kumar, J.Y.; Sornaraj, R. Observation of Pennatulacea (Order) From Gulf of Mannar Biosphere Reserve, Indian. *Scholars Acad. J. Biosci.* **2013** 1(6), 309-312
73. Tizard, T.H.; Moseley, H.N.; Buchanan, H.Y.; Murray, J. Narrative of the cruise of H.M.S. Challenger with a general account of the scientific results of the expedition. Report on the Scientific Results of the Voyage of the H.M.S. Challenger during the years 1873-76. *Narrative* **1885** 1 (first part), 1–509.
74. Harvey, E.N. Studies on bioluminescence: VI. Light production by a Japanese Pennatulid, C*avernularia haberi*. *Am. J. Physiol.* **1917** 42(2), 349-358.
75. Cormier, M.J.; Hori, K.; Anderson, J.M. Bioluminescence in coelenterates. *Biochim. Biophys. Acta Bioenerg.* **1974** 346(2), 137-164. (doi:10.1016/0304-4173(74)90007-X)
76. Panceri, P. Etudes sur la Phosphorescence des Animaux Marins. 6. Sur un Pennatulaire phosphorescent encore inconnu dans les environs de Naples. *Ann. Sci. Nat.* **1872** 5(16), 387–390.
77. Kumar, J.S.; Raghunathan, C.; Venkataraman, K. New Records of Octocorallia (Order: Pennatulacea) from Indian Waters. *Int. J. Appl. Biol. Pharm.* **2014** 5(2), 52-56.

78. Nicol, J.A. Observations on the luminescence of *Pennatula phosphorea*, with a note on the luminescence of *Virgularia mirabilis*. *J. Mar. Biol. Assoc. U. K.* **1958** 37(3):551–563.
79. Cutress, C.E.; Pequegnat, W.E. Three new species of *Zoantharia* from California. *Pac. Sci.* **1960** 14, 89-100.
80. Latz, M.I.; Frank, T.M.; Case, J.F. Spectral composition of bioluminescence of epipelagic organisms from the Sargasso Sea. *Mar. Biol.* **1988** 98:441 – 6. (doi:10.1007/BF00391120)
81. Mackie, G.O. Defensive strategies in planktonic coelenterates. *Mar. Freshw. Behav. Physiol.* **1995** 26(2-4), 119-129. (doi:10.1080/10236249509378933)
82. Hartlaub, C.C. Craspedote Medusen. Teil 1, Lieferung 3, Tiaridae. *Nordisches Plankton* **1914** 6, 237-363.
83. Davenport, D.; Nicol, J.C. Luminescence in hydromedusae. *Proc. R. Soc. B.* **1955** 144(916), 399-411. (doi:10.1098/rspb.1955.0066)
84. Gershwin, L.A.; Zeidler, W.; Davie, P.J. Medusae (Cnidaria) of Moreton Bay, Queensland, Australia. *Mem. Queensl. Mus.* **2010** 54(3), 47-108.
85. Clarke, G.L., Conover, R.J., David, C.N.; Nicol, J.A.C. Comparative studies of luminescence in copepods and other pelagic marine animals. *J. Mar. Biol. Assoc. U. K.* **1962** 42(3), 541-564. (doi:10.1017/S0025315400054254)
86. Morin, J.G.; Hastings, J.W. Energy transfer in a bioluminescent system. *J. Cell. Physiol.* **1971** 77(3), 313-318. (doi:10.1002/jcp.1040770305)
87. Russel, F.S. The medusae of the British Isles. Cambridge University Press, Cambridge, UK, 1953; 529 pp.
88. Levine, L.D.; Ward, W. (1982). Isolation and characterization of a photoprotein," phialidin", and a spectrally unique green-fluorescent protein from the bioluminescent jellyfish *Phialidium gregarium. Comp. Biochem. Physiol. B*. **1982** 72(1), 77-85. (doi:10.1016/0305-0491(82)90013-X)
89. Kato, K. On a luminous hydroid, *Clytia linearis*. *Zool. Mag. Tokyo* **1949** 58, 17-18.
90. Morin, J.G. Coelenterata bioluminescence. In *Coelenterate Biology: Reviews and New Perspectives*; Muscatine, L.; Lenhoff, H.M., Eds., Academic Press, New York, USA, 1974; pp. 397 – 438.
91. Panceri, P. Luminous Campanulariae. *Nature* **1877** 16, 30.
92. Romanes, G.J. An account of some new species, varieties, and monstrous forms of medusae. *Zool. J. Linn. Soc.* **1876** 12(64), 524-531.
93. Widder, E.A.; Bernstein, S.A.; Bracher, D.F.; Case, J.F.; Reisenbichler, K.R.; Torres, J.J.; Robison, B.H. Bioluminescence in the Monterey Submarine Canyon: image analysis of video recordings from a midwater submersible. *Mar. Biol.* **1989** 100(4), 541-551. (doi:10.1007/BF00394831)
94. Morin, J.G.; Reynolds, G.T. Luminescence and related fluorescence in coelenterates. *Biol. Bull.* **1970** 139 (2), 430-431.
95. Gosse, P.H. (1853). *A naturalist's rambles on the Devonshire coast* (Vol. 11): John Van Voorst, London, UK, 1853; pp. 1-451.
96. Freeman, G.; Ridgway, E.B. Endogenous photoproteins, calcium channels and calcium transients during metamorphosis in hydrozoans. *Roux's Arch. Dev. Biol.* **1987** 196, 30-50. (doi:10.1007/BF00376020)
97. Widder, E.A. Midwater Bioluminescence Assessment in the West Alboran Gyre (Mediterranean Sea) - Cruise Report RN Seward Johnson April 5-25, 1991 Cruise No. 90-092. Harbor Branch Oceanographic Institution: Gainesville, Florida, USA, 1991; pp. 1-100.
98. Lapota, D.; Galt, C.; Losee, J.R.; Huddell, H.D.; Orzech, J.K.; Nealson, K.H. (1988). Observations and measurements of planktonic bioluminescence in and around a milky sea. *J. Exp. Mar. Biol. Ecol.* **1988** 119(1), 55-81. (doi:10.1016/0022-0981(88)90152-9)
99. Haddock, S.H.D.; Pugh, P.R.; Mills, C.E.; Harbison, G.R. Medusae, siphonophores and ctenophores of the Alborán Sea, southwestern Mediterranean. *Sci. Mar.* **1996** 60(1), 145-163.
100. Pugh, P.; Haddock, S.H.D. Description of two new species of the genus *Erenna* (Siphonophora: Physonectae: Erennidae), with notes on recently collected specimens of other *Erenna* species. *Zootaxa* **2016** 4189(3), 401. (doi:10.11646/zootaxa.4189.3.1)
101. Haddock, S.H., Dunn, C.W.; Pugh, P.R.; Schnitzler, C.E. Bioluminescent and red-fluorescent lures in a deep-sea siphonophore. *Science* **2005** 309(5732), 263-263. (doi:10.1126/science.1110441)
102. Pugh, P.R. A revision of the family Forskaliidae (Siphonophora, Physonectae). *J. Nat. Hist.* **2003** 37, 1281-1327. (doi:10.1080/00222930110120638)
103. Nicol, J.A.C. Observations on Luminescence in Pelagic Animals. *J. Mar. Biol. Assoc. U. K.* **1958** 37, 705-752. (doi:10.1017/S0025315400005749)
104. Haddock, S.H.D.; Dunn, C.D.; Pugh, P.R. A re-examination of siphonophore terminology and morphology, applied to the description of two new prayine species with remarkable bio-optical properties. *J. Mar. Biol. Assoc. U. K.* **2005** 85 (3), 695-707. (doi:10.1017/S0025315405011616)
105. Pugh, P.R. A review of the family Sphaeronectidae (class Hydrozoa, order Siphonophora), with the description of three new species. *Zootaxa* **2009** 2147, 1-48. (doi:10.11646/zootaxa.2147.1.1)
106. The Bioluminescence Web Page. Available online: https://biolum.eemb.ucsb.edu/ (accessed on 15 January 2024)

107. Herring, P.J.; Widder, E.A. Bioluminescence of deep-sea coronate medusae (Cnidaria: Scyphozoa). *Mar. Biol.* **2004** 146, 39-51. (doi:10.1007/s00227-004-1430-7)
108. Gershwin, L.A.; Collins, A.G. A preliminary phylogeny of Pelagiidae (Cnidaria, Scyphozoa), with new observations of *Chrysaora colorata* comb. nov. *J. Nat. Hist.* **2002** 36(2), 127-148. (doi:10.1080/00222930010003819)
109. Kato, K. A new luminous species of the Nemertea, *Emplectonema kandai* sp. nov. *Jpn. J. Zool.* **1939** 8(2), 251-254.
110. Haneda, Y. Luminous organisms of Japan and the Far East. In *The Luminescence of Biological Systems*; Johnson, F.H., Ed; American Association for the Advancement of Science, Washington, DC, USA, 1955; pp. 335.
111. Knox, G.A.; Green, K.M. The Polychaetes of New Zealand part 3: Lysaretidae. *J. R. Soc. N. Z.* **1972** 2(3), 431-434. (doi:10.1080/03036758.1972.10421827)
112. Ben-Eliahu, M.N. Polychaete cryptofauna from rims of similar intertidal vermetid reefs on the Mediterranean coast of Israel and in the Gulf of Elat: Sedentaria. *Isr. J. Ecol. Evol.* **1976** 25(4), 121-155. (doi:10.1080/00212210.1976.10688434)
113. Kin, I.; Oba, Y. Bioluminescent properties of *Mesochaetopterus japonicus* (Polychaeta: Chaetopteridae) with comparison to *Chaetopterus*, *Plankton Benthos Res.* **2020**, 15(3), 228-231. (doi:10.3800/pbr.15.228)
114. Deheyn, D.D.; Enzor, L.A.; Dubowitz, A.; Urbach, J.S.; Blair, D. Optical and physicochemical characterization of the luminous mucous secreted by the marine worm *Chaetopterus sp. Physiol. Biochem. Zool.* **2013** 86(6), 702-715. (doi:10.1086/673869)
115. Nishi, E., Arai, H.; Sasanuma, S.-I. A new species of *Chaetopterus* (Polychaeta: Chaetopteridae) from off Tokyo Bay, Central Japan, with comments on its bioluminescence. *Actinia* **2000** 13, 1–12.
116. M'Intosh, W.C. On the abyssal theory of light, the protozoic-absorption theory, and the azoic-mud theory, propounded in the reports of HMS ‘Porcupine,’ 1869 and 1870. *Ann. Mag. Nat. Hist.* **1872** 9(49), 1-13.
117. Anctil, M. Luminescence control in isolated notopods of the tube-worm *Chaetopterus variopedatus*: effects of cholinergic and GABAergic drugs. *Comp. Biochem. Physiol. C Toxicol. Pharmacol.* **1981** 68(2), 187-194. (doi:10.1016/0306-4492(81)90014-9)
118. Osborn, K.J.; Rouse, G.W.; Goffredi, S.K.; Robison, B H. Description and relationships of *Chaetopterus pugaporcinus*, an unusual pelagic polychaete (Annelida, Chaetopteridae). *Biol. Bull.* **2007** 212(1), 40-54. (doi:10.2307/25066579)
119. Nicol, J.A.C. Spectral composition of the light of *Chaetopterus*. J. Mar. Biol. Assoc. U. K. 1957 36(3), 629-642. (doi: 10.1017/S0025315400025893)
120. Nishi, E.; Hickman, C.P.; Bailey-Brock, J.H. *Chaetopterus* and *Mesochaetopterus* (Polychaeta: Chaetopteridae) from the Galapagos Islands, with descriptions of four new species. *Proc. Acad. Nat. Sci. Philadelphia* **2009** 158, 239-259. (doi:10.1635/053.158.0113)
121. Nishi, E.; Hsieh, H.-L. Chaetopterid polychaetes from Taiwan and Okinawa Island (Japan), with descriptions of two new species. *Zool. Stud.* **2009** 48(3), 370-379.
122. Nishi, E. Redescription of *Mesochaetopterus selangolus* (Polychaeta: Chaetopteridae) based on type specimens and recently collected material from Morib Beach, Selangor State, Malaysia. *Pac. Sci.* **1999** 53: 24-36.
123. Zhang, Y.; Rouse, G.W.; Qiu, J.-W. A new species of *Mesochaetopterus* (Annelida, Chaetopteridae) from Hong Kong, with comments on the phylogeny of the family. *Zootaxa* **2015** 3974(4): 495-506. (doi:10.11646/zootaxa.3974.4.2)
124. Petersen, J.A.; Fanta, E.S. On two new species of *Mesochaetopterus* (Polychaeta) from the Brazilian coast. *Beitr. Neotrop. Fauna* **1969** 6(2): 120-136. (doi:10.1080/01650526909360422)
125. Raymond, J.A.; DeVries, A.L. Bioluminescence in McMurdo Sound, Antarctica. *Limnol. Oceanogr.* **1976** 21(4), 599-602. (doi:10.4319/lo.1976.21.4.0599)
126. Lecuyer, B.; Arrio, B. Some spectral characteristics of the light emitting system of the polynoid worms. *Photochem. Photobiol.* **1975** 22(5), 213-215. (doi:10.1111/j.1751-1097.1975.tb06738.x)
127. Nicol, J.A.C. Spectral composition of the light of polynoid worms. *J. Mar. Biol. Assoc. U. K.* **1957** 36, 529 – 38. (doi:10.1017/S0025315400025820)
128. Andrews, E.A. Report upon the Annelida Polychaeta of Beaufort, North Carolina. *Proc. U. S. Natl. Mus.* **1891** 14(852), 277-302.
129. Salazar-Silva, P. Redescription of *Harmothoe aculeata* (Polychaeta: Polynoidae) and description of three new similar species from the Grand Caribbean region. *J. Mar. Biol. Assoc. U. K.* **2003** 83(1), 55-64. (doi:10.1017/S0025315403006805h)
130. Khvorostanski, C. Sur la lumination des animaux de la mer Blanche. *Inter. Congr. Zool. Moscow* **1892** 2, 185-186.
131. Fauvel P. Polychètes errantes. Faune de France: LeChavelier, Paris, France, 1923; pp. 1-488.
132. Darboux, J.G. Recherche sur les Aphroditiens. *Trav. Instn. Zool. U Montpellier et Stat. Mar. Cette. Mem.* **1899** 6.
133. Herrera, A.A. Electrophysiology of bioluminescent excitable epithelial cells in a polynoid polychaete worm. *J. Comp Physiol.* **1979** 129, 67-78. (doi:10.1007/BF00679913)
134. Lloyd, R.E.; Captain, I.M.S.; WILLEY, A.; Muscum, C. Notes on the phosphorescence in marine animals. *Rec. Indian Mus.* **1907** 1, 257.

135. Nicol, J. Luminescence in polynoid worms. *J. Mar. Biol. Assoc. U. K.* **1953** 32(1), 65-84. (doi:10.1017/S0025315400011437)
136. Taboada, S. ; Serra Silva, A.; Díez-Vives, C.; Neal, L.; Cristobo, J. ; Ríos, P.; Hestetun, T.J., Clark, B.; Rossi, M.E., Junoy, j.; Navarro, J.; Riesgo, A. Sleeping with the enemy: unravelling the symbiotic relationships between the scale worm *Neopolynoe chondrocladiae* (Annelida: Polynoidae) and its carnivorous sponge hosts. *Zool. J. Linn. Soc.* **2021** 193(1), 295-318. (doi:10.1093/zoolinnean/zlaa146)
137. Panceri, P. Catalogo degli Anellidi, Gefirei e Turbellarie d'Italia. *Atti Soc. Ital. Sc. N. Milano* **1875** 18, 2-3.
138. Pettibone, M.H. Marine polychaete worms of the New England region. I. Aphroditidae through Trochochaetidae. *Bull. U.S. Natl. Mus.* **1963** 227 (part 1), 1–356. (doi:10.5479/si.03629236.227.1)
139. Bassot, J.M. Sites actifs et facilitation dans trois systèmes bioluminescents. *Arch. Zool. Exper. Gen.* **1979** 120(1), 5-24.
140. Verdes, A., Álvarez-Campos, P.; Nygren, A.; San Martín, G.; Deheyn, D.D.; Gruber, D.F.; Holford, M. Molecular phylogeny and evolution of bioluminescence in *Odontosyllis* (Annelida, Syllidae). *Invert. System.* **2022** 36(7), 622-630. (doi :10.1071/IS22007)
141. Shimomura, O.; Johnson, F.H.; Saiga, Y. Partial purification and properties of the *Odontosyllis* luminescence system. *J. Cell. Comp. Physiol.* **1963** 61, 275 – 92. (doi:10.1002/jcp.1030590302)
142. Atputhanathan, M. Study on two annelids worms *Pontodrilus bermudensis*, Beddard,1891, *Odontosyllis gravelyi*, Fauvel, 1928. In *Hydrobiological Survey of the Thondaimannar Lagoon Bulletin no. 6.*; Kugathasan, K.S., Ed.; Hydrobiological Survey Research Council of the Northern Province Science Teachers Association: Jaffna, Sri Lanka, 1968; pp. 1-73 pp.
143. Fukuda, M.V.; de Matos Nogueira, J.M. A new species of *Odontosyllis* Claparede, 1863 (Polychaeta: Syllidae: Eusyllinae), and description of Brazilian material of *Odontosyllis cf. fulgurans* (Audouin and Milne Edwards, 1834). *Zool. Stud.* **2006** 45(2), 223-233.
144. van Lummel, L.A.E. Over lichtende wormpjes in de baai van Batavia. *De Trop. Nat.* **1932** 21(6), 85-87.
145. Gaston, G.R.; Hall, J. Lunar periodicity and bioluminescence of swarming *Odontosyllis luminosa* (Polychaeta: Syllidae) in Belize. *Gulf Caribb. Res.* **2000** 12(1), 47-51. (doi:10.18785/gcr.1201.07)
146. Mitani, Y.; Yasuno, R.; Futahashi, R.; Ohmiya, Y. Luciferase gene of a Caribbean fireworm (Syllidae) from Puerto Rico. *Sci. Rep.* **2019** 9, 13015. (doi:10.1038/s41598-019-49538-7)
147. Ehrenberg, C.G. Das Leuchten des Meeres. Neue Beobachtungen nebst Übersicht der Hauptmomente der geschichtlichen Entwiclung dieses merkwürdigen Phänomens. *Konigl. Akad. Wiss.* **1836** 1834, 411–575.
148. Deheyn, D.D.; Latz, M.I. Internal and secreted bioluminescence of the marine polychaete *Odontosyllis phosphorea* (Syllidae). *Invertebr. Biol.* **2009** 12,31–45. (doi:10.1111/j.1744-7410.2008.00149.x)
149. Daly, J. Reversible epitoky in the life history of the polychaete *Odontosyllis polycera* (Schmarda 1861). *J. Mar. Biol. Assoc. U. K.* **1975** 55(2), 327-344. (doi:10.1017/S0025315400015976)
150. Mitani, Y.; Yasuno, R.; Isaka, M.; Mitsuda, N.; Futahashi, R.; Kamagata, Y.; Ohmiya, Y. Novel gene encoding a unique luciferase from the fireworm *Odontsyllis undecimdonta*. *Sci. Rep.* **2018** 8(1), 1-7. (doi:10.1038/s41598-018-31086-1)
151. Bonhomme, C. La Bioluminescence de quelques annélides méditerranéennes:(Etude histologique et hystophysiologique). *Nat. Monspel., Sér. Zool.* **1958** 2, 7-137.
152. Gouveneaux, A.; Flood, P.R.; Mallefet, J. Unexpected diversity of bioluminescence in planktonic worms. *Luminescence* **2017** 32(3), 394-400. (doi:10.1002/bio.3192)
153. Evstignev, P.V.; Williams, R.; Piontkovski, S.A.; Bileva, O.K. Spatio-temporal succession and distribution of bioluminescent organisms of the central Atlantic Ocean. In *Proceedings of the 6th International Symposium on Bioluminescence and Chemiluminescence*, Cambridge, UK, 5-8 September 1994.
154. Salerno, V. Ulteriori particolari sulla conduzione del materiale presente nelle pinne di *Tomopteris Johnstonella nationalis* Apstein. *Atti R. Accad. Pelorit., Cl. Sci. Fis. Mat. Biol.* **1965** 11, 203-209.
155. Greeff, R. Ueber die rosettenförmigen leuchtorgane der tomopteriden und zwei neue arten von *Tomopteris*. *Zool. Anz.* **1882** 5, 384-7.
156. Oliveira, A.G.; Amaral, D.T.; Hannon, M.C.; Schulze, A. First record of bioluminescence in a sipunculan worm. *Front. Mar. Sci.* **2021** 8, 762706. (doi:10.3389/fmars.2021.762706)
157. Gibbs, P.E. Polychaete annelids from the Cook Islands. *J. Zool.* **1972** 168(2), 199-220. (doi:10.1111/j.1469-7998.1972.tb01347.x)
158. Averincev, V.G. *Chauvinelia arctica*, sp. n. (Acrocirridae, Polychaeta) from the Canadian plain. *Issled. Fauny Morej Zool. Inst., Akad. Nauk SSSR* **1980** 25: 57–62.
159. Osborn, K.J.; Haddock, S.H.; Pleijel, F.; Madin, L.P.; Rouse, G.W. Deep-sea, swimming worms with luminescent "bombs". *Science* **2009** 325(5943), 964-964. (doi:10.1126/science.1172488)
160. Osborn, K.J., Haddock, S.H.; Rouse, G.W. *Swima* (Annelida, Acrocirridae), holopelagic worms from the deep Pacific. *Zool. J. Linn. Soc.* **2011** 163(3), 663-678. (doi:10.1111/j.1096-3642.2011.00727.x)
161. Bonhomme, C. La luminescence de *Heterocirrus bioculatus* Keferstein. *Bull. Inst. Océanogr. Monaco* **1944** 871, 2 – 7.

162. Petersen, M.E. Reproduction and development in Cirratulidae (Annelida: Polychaeta). *Hydrobiologia* **1999** 402, 107 – 128. (doi:10.1023/A:1003736408195)
163. Verrill, A.E. Notice of the remarkable marine fauna occupying the outer banks off the southern coast of New England, No. 7, and of some additions to the fauna of Vineyard Sound. *Am. J. Sci.* **1882** 24, 360 – 371.
164. Gibbs, P.E. A comparative study of reproductive cycles in four polychaete species belonging to the family Cirratulidae. *J. Mar. Biol. Ass. U. K.* **1971** 51, 745 – 769. (doi:10.1017/S002531540001794X)
165. Berkeley, E.; Berkeley, C. Notes on Polychæta from the coast of Western Canada. *J. Nat. Hist.* **1950** 3(25), 50-69.
166. Molisch, H. *Leuchtend Pflanzen Eine physiologische Studie*. Gustav Fischer Verlag: Jena, Germany, 1904; pp.1-198.
167. Kin, I.; Jimi, N.; Oba, Y. Bioluminescence properties of *Thelepus japonicus* (Annelida: Terebelliformia). *Luminescence* **2019** 34(6), 602-606. (doi:10.1002/bio.3643)
168. Living Lights List. Available online: https://www3.chubu.ac.jp/faculty/oba_yuichi/living_light_list/ (accessed on 15 January 2024)
169. Francis, W.R.; Powers, M.L.; Haddock, S.H.D. Bioluminescence spectra from three deep-sea polychaete worms. *Mar. Biol.* **2016** 163, 255. (doi:10.1007/s00227-016-3028-2)
170. Jimi, N.; Bessho-Uehara, M.; Nakamura, K.; Sakata, M.; Hayashi, T.; Kanie, S.; Mitani, Y.; Ohmiya, Y.; Tsuyuki, A.; Ota, Y.; Woo, S.P.; Ogoh, K. Investigating the diversity of bioluminescent marine worm *Polycirrus* (Annelida), with description of three new species from the Western Pacific. *R. Soc. Open Sci.* **2023** 10(3), 230039. (doi:10.1098/rsos.230039)
171. Dahlgren, U. The production of light by animals. Light production as seen in worms. *J. Franklin Inst.* **1916** 181, 659-696.
172. Bonhomme, C. Sur un mode particulier d'élimination des produits photogenes chez *Polycirrus caliendrum* Clap., et *Polycirrus aurantiacus* Grube. *Bull. Soc. Zool. Fr*. **1953** 77, 341.
173. Huber, M.E.; Arneson, C.A.; Widder, E.A. Extremely blue bioluminescence in the polychaete *Polycirrus perplexus* (Terebellidae). *Bull. Mar. Sci.* **1989** 44(3), 1236-1239.
174. Verrill, A.E. Notice of recent additions to the marine invertebrata of the northeastern coast of America, with descriptions of new genera and species and critical remarks on others. Part I. Annelida, Gephyraea, Nemertina, Nematoda, Polyzoa, Tunicata, Mollusca, Anthozoa, Echinodermata, Porifera. *Proc. U. S. Natl. Mus.* **1879** 2, 165 – 205.
175. Verrill, A.E. Additions to the Turbellaria, Nemertina, and Annelida of the Bermudas, with revisions of some New England genera and species. *Trans. Conn. Acad.* **1900** 10(2), 595-671.
176. Haneda, Y. Luminous Organisms. Kouseisha-Kouseikaku: Tokyo, Japan, 1985; pp. 1-318.
177. Harvey, E.N. Oxygen and luminescence, with a description of methods for removing oxygen from cells and fluids. *Biol. Bull.* **1926** 51(2), 89-97.
178. Kosuge, T. Bioluminescence of *Cucullaea labiata* (Mollusca: Bivalvia: Cucullaeidae) collected in Vietnam. *J. Nanki Biol. Soc.* **2018** 60(1), 53-55.
179. Haneda, Y. Luminosity in *Rocellaria grandis* (Deshayes)(Lamellibranchia). *Sci. South Seas* **1939** 2, 36-39.
180. Okada, Y.K. Luminescence chez les Mollusques lamellibranches. *Bull. Soc. Zool. Fr.* **1927** 52, 95 – 98.
181. Nicol, J.A.C. (1958). Spectral composition of the light of *Pholas dactylus* L. *J. Mar. Biol. Ass. U. K.* **1958** 37, 43. (doi:10.1017/S0025315400014806)
182. Robison, B.H.; Young, R.E. Bioluminescence in pelagic octopods. *Pac. Sci.* **1981** 35, 39 – 44.
183. Johnsen, S.; Balser, E.J.; Fisher, E.C.; Widder, E.A. Bioluminescence in the deep-sea cirrate octopod *Stauroteuthis syrtensis* Verrill (Mollusca: Cephalopoda). *Biol. Bull.* **1999** 197(1), 26-39. (doi:10.2307/1542994)
184. Guerrero-Kommritz, J.; Cantera, J.; León, J.; Puentes, V. First observation on *Cirrothauma sp*. in the Colombian Southern Caribbean. *Biodiv. Internat. J.* **2018** 2(3), 272-273. (doi:10.15406/bij.2018.02.00072)
185. Collins, M.A.; Henriques, C. A revision of the family Stauroteuthidae (Octopoda: Cirrata) with redescriptions of *Stauroteuthis syrtensis* and *S. gilchristi*. *J. Mar. Biol. Ass. U. K.* **2000** 80(4), 685-697. (doi:10.1017/S0025315400002514)
186. Johnsen, S.; Balser, E.J.; Widder, E.A. Light-emitting suckers in an octopus. *Nature* **1999** 398(6723), 113-114. (doi:10.1038/18131)
187. Lu, C.C. A new family of myopsid squid from Australasian waters (Cephalopoda: Teuthida). *Phuket Mar. Biol. Cent.* **2005** 66: 71-82.
188. Alexeyev, D.O. The systematic position of bioluminescent squids of family Loliginidae (Cephalopoda, Myopsida). *Zool. Zhurnal* **1992** 71: 12 – 23.
189. Anderson, F.E.; Bergman, A.; Cheng, S.H.; Pankey, M.S.; Valinassab, T. Lights out: the evolution of bacterial bioluminescence in Loliginidae. *Hydrobiologia* **2014** 725, 189-203. (doi:10.1007/s10750-013-1599-1)

190. Guerrero-Ferreira, R.C.; Nishiguchi, M.K. Biodiversity among luminescent symbionts from squid of the genera *Uroteuthis*, *Loliolus* and *Euprymna* (Mollusca: Cephalopoda). *Cladistics* **2007** 23, 497–506. (doi:10.1111/j.1096-0031.2007.00155.x)
191. Vecchione, M., Brakoniecki, T.F.; Natsukari, Y.; Hanlon, R.T. A provisional generic classification of the family Loliginidae. *Smithson. Contrib. Zool.* **1998** 586, 215–222.
192. Jereb, P.; Roper, C.F.E. Cephalopods of the world: An annotated and illustrated catalogue of cephalopod species known to date (Volume 2). Myopsid and oegopsid squids; Food and Agriculture Organization of the United Nations: Rome, Italy, 2005, pp. 1-649.
193. Haneda, Y. Observations on the luminescence of the shallow-water squid, *Uroteuthis bartschi*. *Sci. Rep. Yokosuka City Mus.* **1964** 8, 10-16.
194. Thouand, G.; Daniel, P.; Horry, H.; Picart, P.; Durand, M.J.; Killham, K.; Knox, O.C.G.; Dubow, M.S.; Rousseau, M. Comparison of the spectral emission of lux recombinant and bioluminescent marine bacteria. *Luminescence* **2003** 18(3), 145-155. (doi:10.1002/bio.716)
195. Fukasawa, S.; Dunlap, P.V. Identification of luminous bacteria isolated from the light organ of the squid, *Doryteuthis kensaki*. *Agric. Biol. Chem.* **1986** 50(6), 1645-1646. (doi:10.1271/bbb1961.50.1645)
196. Urbanczyk, H., Ast, J.C.; Dunlap, P.V. Phylogeny, genomics, and symbiosis of *Photobacterium*. *FEMS Microbiol. Rev.* **2011** 35(2), 324-342. (doi:10.1111/j.1574-6976.2010.00250.x)
197. Judkins, H.; Lindgren, A.; Villanueva, R.; Clark, K.; Vecchione, M. A description of three new bathyteuthid squid species from the North Atlantic and Gulf of Mexico. *Bull. Mar. Sci.* **2020** 96(2), 281-296. (doi:10.5343/bms.2019.0051)
198. Shea, E.K.; Stadler, J.; Lindgren, A. (2020). *Brachioteuthis beanii* Verrill, 1881 (Cephalopoda: Brachioteuthidae) in the northwest Atlantic. *Bull.Mar. Sci.* **2020** 96(2), 309-322. (doi:10.5343/bms.2019.0073)
199. Lipinski, M.R. Preliminary description of two new species of cephalopods (Cephalopoda: Brachioteuthidae) from South Atlantic and Antarctic waters. *Bull. Natl. Inst. Ocean. Fish.* **2001** 1(152), 3-14.
200. Verhoeff, T.J. A new species of *Stauroteuthis* (Octopoda: Cirrata) and further novel cirrate octopods from Australian waters. *Molluscan Res.* **2023** 1-20. (doi:10.1080/13235818.2023.2232534)
201. Braid, H.E. Resolving the taxonomic status of *Asperoteuthis lui* Salcedo-Vargas, 1999 (Cephalopoda, Chiroteuthidae) using integrative taxonomy. *Mar. Biodivers.* **2017** 47, 621-635. (doi:10.1007/s12526-016-0547-5)
202. Tree of Life Web Project. Available online: http://tolweb.org/tree/ (accessed on 15 January 2024)
203. Kubota, T.; Koshiga, M.; Okutani, T. Rare and Interesting Squid from Japan VII: Some Biological Data on *Chiroteuthis imperator* from Suruga Bay, Japan (Cephalopoda: Chiroteuthidae). *Venus* **1981** 40(3), 150-159. (doi:10.18941/venusjjm.40.3_150)
204. Joubin, L. Notes préliminaires sur les Céphalopodes des croisieres du Dana (1921-1922). 4e Partie. *Ann. Inst. Oceanogr.* **1933** 13(1), 1-49.
205. Salcedo-Vargas, M.A. Cephalopods from the Netherlands Indian Ocean Programme (NIOP)-I. *Chiroteuthis spoeli* n. spec. and *Chiroteuthis picteti somaliensis* n. subspec. *Beaufortia* **1996** 46(2), 11-26.
206. Battaglia, P.; Canese, S.; Salvati, E.; Greco, S. *In situ* observations of three deep-sea cephalopods in the central Mediterranean Sea. *Mar. Biol.* **2023** 170(11), 151. (doi:10.1007/s00227-023-04264-7)
207. Joubin, L. Observations sur divers Céphalopodes. Quatrième note: *Grimalditeuthis richardi* Joubin 1898. *Bull. Soc. Zool. Fr.* **1898** 23, 101-113.
208. Silva-Dávila, R.D.; Avendaño-Ibarra, R.; Young, R.E.; Hochberg, F.G.; Hernández-Rivas, M.E. First record and description of *Planctoteuthis* (Cephalopoda: Chiroteuthidae) paralarvae in the Gulf of California, Mexico. *Lat. Am. J. Aquat. Res.* **2018** 46(2), 280-288. (doi :10.3856/vol46-issue2-fulltext-4)
209. Escánez, A.; Roura, Á.; Riera, R.; González, Á.F.; Guerra, Á. New data on the systematics of comb-fin squids *Chtenopteryx spp.* (Cephalopoda: Chtenopterygidae) from the Canary Islands. *Zool. Stud.* **2018** 57. (doi:10.6620/ZS.2018.57-40)
210. Voss, N.A. A generic revision of the Cranchiidae (Cephalopoda; Oegopsida). *Bull. Mar. Sci.* **1980** 30(2), 365-412.
211. Kubodera T.; Okutani, T. An unusual squid from the Sea of Japan, *Enigmocranchia nipponica*, new genus and new species (Cephalopoda: Decembrachiata: Cranchiidae). *Natl. Mus. Nat. Sci. Monogr.* **2014** 44, 149-156.
212. Judkins, H.; Rose-Mann, L.; Lindgren, A.; Taite, M.; Bush, S.; Vecchione, M. A newly discovered *Helicocranchia* species (Cephalopoda: Cranchiidae: Taoniinae) in the northern Gulf of Mexico. Bull. Mar. Sci. **2022** 98(3), 419-430. (doi:10.5343/bms.2021.0048)
213. Evans, A.B.; Bolstad, K.S.R. Diversity of the squid genus *Leachia* (Oegopsida: Cranchiidae) in the Pacific Ocean. *Mar. Biol.* **2023** 170(6), 72. (doi:10.1007/s00227-023-04215-2)
214. Berry, S.S. Cephalopods of the Kermadec Islands. *Proc. Acad. Nat. Sci. Phila.* **1916** 68: 45–66

215. Young, R.E.; Arnold, J.M. The functional morphology of a ventral photophore from the mesopelagic squid, *Abralia trigonura*. *Malacologia* **1982** 23(1), 135-163.
216. Herring, P.J.; Widder, E.A.; Haddock, S.H.D. Correlation of bioluminescence emissions with ventral photophores in the mesopelagic squid *Abralia veranyi* (Cephalopoda: Enoploteuthidae). *Mar. Biol.* **1992** 112(2), 293-298. (doi:10.1007/BF00702474)
217. Tsuji, F.I. Bioluminescence reaction catalyzed by membrane-bound luciferase in the "firefly squid," *Watasenia scintillans*. *Biochim. Biophys. Acta Biomembr.* **2002** 1564(1), 189-197. (doi:10.1016/S0005-2736(02)00447-9)
218. Young, R.E. The systematics and areal distribution of pelagic cephalopods from the seas off Southern California. *Smithson. Contrib. Zool.* **1972** 97, 159. (doi:10.5479/si.00810282.97)
219. Voss, N.A.; Nesis, K.N.; Rodhouse, P.G. The cephalopod family Histioteuthidae (Oegopsida): systematics, biology, and biogeography. *Smithson. Contrib. Zool.* **1998** 586, 293-372.
220. Vérany, J.B. Mollusques méditeranéens: Observés, décrits, figurés, et chromolithographiés d'après le vivant. Première partie: Céphalopodes de la Méditerranée. GêneImprimerie des Sourds-Muets: Gênes, France, 1851; pp. 1-130.
221. Dilly, P.N.; Herring, P.J. Ultrastructural features of the light organs of *Histioteuthis macrohista* (Mollusca: Cephalopoda). *J. Zool.* **1981** 195(2), 255-266. (doi:10.1111/j.1469-7998.1981.tb03463.x)
222. Young, R.E. Function of the dimorphic eyes in the midwater squid *Histioteuthis dofleini*. *Pac. Sci.* **1975** 29, 211–218.
223. Arocha, F. A new species of *Nematolampas* (cephalopoda: Oegopsida) from the Western Central Atlantic with an overview of the family Lycoteuthidae. *Bull Mar. Sci.* **2003** 72(3), 941-953.
224. Berry, S.S. Light Production in Cephalopods, I: An Introductory Survey. *Biol. Bull.* **1920** 38(3), 141-169. (doi:10.2307/1536213)
225. Braid, H.E.; McBride, P.D.; Bolstad, K.S. Molecular phylogenetic analysis of the squid family Mastigoteuthidae (Mollusca, Cephalopoda) based on three mitochondrial genes. *Hydrobiologia* **2014** 725, 145-164. (doi:10.1007/s10750-013-1775-3)
226. Kelly, J.T. Systematics of the Octopoteuthidae Berry, 1912 (Cephalopoda: Oegopsida). PhD thesis, Auckland University of Technology, Auckland, New Zealand, 30 July 2019.
227. Bush, S.L.; Robison, B.H.; Caldwell, R.L. Behaving in the dark: locomotor, chromatic, postural, and bioluminescent behaviors of the deep-sea squid *Octopoteuthis deletron* Young 1972. *Biol. Bull.* **2009** 216(1), 7-22. (doi:10.2307/25470719)
228. Young, R.E.; Roper, C.F.E. Intensity regulation of bioluminescence during countershading in living animals. Fish. Bull. **1976** 75, 239-252.
229. Herring, P.J.; Dilly, P.N.; Cope, C. Different types of photophore in the oceanic squids *Octopoteuthis* and *Taningia* (Cephalopoda: Octopoteuthidae). *J. Zool.* **1992** 227(3), 479-491. (doi:10.1111/j.1469-7998.1992.tb04408.x)
230. Galeazzo, G.A.; Mirza, J.D.; Dorr, F.A.; Pinto, E.; Stevani, C.V.; Lohrmann, K.B.; Oliveira, A.G. Characterizing the Bioluminescence of the Humboldt Squid, *Dosidicus gigas* (d'Orbigny, 1835): One of the Largest Luminescent Animals in the World. *Photochem. Photobiol.* **2019** 95(5), 1179-1185. (doi:10.1111/php.13106)
231. Sasaki, M. On three interesting new oegopsids from the Bay of Sagami. *J. Coll. Agric. Tohoku Imp. Univ.* **1915** 6 (6): 131–150.
232. Fernández-Álvarez, F.Á.; Braid, H.E.; Nigmatullin, C.M.; Bolstad, K.S.; Haimovici, M.; Sánchez, P.; Sajikumar, K.K.; Ragesh, N.; Villanueva, R. Global biodiversity of the genus *Ommastrephes* (Ommastrephidae: Cephalopoda): an allopatric cryptic species complex. *Zool. J. Linn. Soc.* **2020** 190(2), 460-482. (doi:10.1093/zoolinnean/zlaa014)
233. Tsuji, F.I.; Leisman, G.B. K+/Na+-triggered bioluminescence in the oceanic squid *Symplectoteuthis oualaniensis*. *Proc. Natl. Acad. Sci. U.S.A.* **1981** 78(11), 6719-6723. (doi:10.1073/pnas.78.11.6719)
234. Girsch, S.J.; Herring, P.J.; McCapra, F. Structure and preliminary biochemical characterization of the bioluminescent system of *Ommastrephes pteropus* (Steenstrup)(Mollusca: Cephalopoda). *J. Mar. Biol. Ass. U. K.* **1976** 56(3), 707-722. (doi:10.1017/S0025315400020749)
235. Bolstad, K.S. 2010. Systematics of the Onychoteuthidae Gray, 1847 (Cephalopoda: Oegopsida). *Zootaxa* **2010** 2696: 1–186. (doi:10.11646/zootaxa.2696.1.1)
236. Fernández-Álvarez, F.Á.; Colmenero, A.I.; Barría, C. First record of the elusive oceanic squid *Thysanoteuthis rhombus* Troschel, 1857 (Cephalopoda: Thysanoteuthidae) in the Catalan coast. *Graellsia* **2021** 77(1):e122. (doi:10.3989/graellsia.2021.v77.284)
237. Lindgren, A.R. Systematics and distribution of the squid genus *Pterygioteuthis* (Cephalopoda: Oegopsida) in the eastern tropical Pacific Ocean. *J. Molluscan Stud.* **2010** 76(4), 389-398. (doi:10.1093/mollus/eyq028)

238. Bello, G. Evolution of the hectocotylus in Sepiolinae (Cephalopoda: Sepiolidae) and description of four new genera. *Eur. J. Taxon.* **2020** 655, 1–53. (doi:10.5852/ejt.2020.655)
239. Tanet, L.; Martini, S.; Casalot, L.; Tamburini, C. Reviews and syntheses: Bacterial bioluminescence – ecology and impact in the biological carbon pump. *Biogeosciences* **2020** 17(14), 3757-3778. (doi:10.5194/bg-17-3757-2020)
240. Young, R.E.; Vecchione, M.; Roper, C.F. A new genus and three new species of decapodiform cephalopods (Mollusca: Cephalopoda). *Rev. Fish Biol. Fish.* **2007** 17, 353-365. (doi:10.1007/s11160-007-9044-z)
241. Lu, C.C.; Okutani, T. Two new genera and species of sepioline squids (Cephalopoda: Sepiolidae) from Australia. *Mem. Mus. Vic.* **2022** 81, 1-23. (doi:10.24199/j.mmv.2022.81.01)
242. Lu, C.C.; Boucher-Rodoni, R. A new genus and species of sepiolid squid from the waters around Tonga in the central South Pacific (Mollusca: Cephalopoda: Sepiolidae). *Zootaxa* **2006** 1310(1), 37-51. (doi:10.11646/zootaxa.1310.1.2)
243. Jones, B.W.; Nishiguchi, M.K. Counterillumination in the Hawaiian bobtail squid, *Euprymna scolopes* Berry (Mollusca: Cephalopoda). *Mar. Biol.* **2004** 144, 1151 – 1155. (doi:10.1007/s00227-003-1285-3)
244. Voss, G.L. The cephalopoda obtained by the Harvard-Havana expedition off the coast of Cuba in 1938-39. *Bull. Mar. Sci.* **1955** 5, 81-115.
245. Kubodera, T.; Okutani, T. New additions of luminous bobtail squids to the Japanese cephalopod fauna (Sepiolida: Sepiolidae: Heteroteuthinae). *Venus* **2011** 69(3-4), 145-161.
246. Harman, R.F.; Seki, M.P. *Iridoteuthis iris* (Cephalopoda: Sepiolidae): new records from the Central North Pacific and first description of the adults. *Pac. Sci.* **1990** 44(2), 171-179.
247. Reid, A.L. Two new species of *Iridoteuthis* (Cephalopoda: Sepiolidae: Heteroteuthinae) from the southwest Pacific, with a redescription of *Stoloteuthis maoria* (Dell, 1959). *Zootaxa* **2021** 5005(4), 503-537. (doi:10.11646/zootaxa.5005.4.3)
248. Guerrero-Kommritz, J. ; Rodriguez-Bermudez, A. Sepiolids (Mollusca: Cephalopoda) from the southern Caribbean, Colombian coast, and a redescription of *Nectoteuthis pourtalesii* Verrill, 1883. *Mar. Biodivers.* **2017** 47, 203-224. (doi:10.1007/s12526-016-0462-9)
249. Jereb, P.; Roper, C.F.E. Cephalopods of the world: An annotated and illustrated catalogue of cephalopod species known to date (Volume 1). Chambered nautiluses and sepioids; Food and Agriculture Organization of the United Nations: Rome, Italy, 2005, pp. 1-262.
250. Sanchez, G.; Fernández-Álvarez, F.Á.; Taite, M., Sugimoto, C.; Jolly, J.; Simakov, O.; Marlétaz, F.; Allcock, L.; Rokhsar, D.S. Phylogenomics illuminates the evolution of bobtail and bottletail squid (order Sepiolida). *Commun. Biol.* **2021** 4(1), 819. (doi:10.1038/s42003-021-02348-y)
251. Fernández-Álvarez, FÁ.; Sánchez P.; Villanueva R. Morphological and molecular assessments of Bobtail Squids (Cephalopoda: Sepiolidae) reveal a hidden history of biodiversity. *Front. Mar. Sci.* **2021** 7 632261, 1-21. (doi:10.3389/fmars.2020.632261)
252. Relini, L.O.; Massi, D. The butterfly squid *Stoloteuthis leucoptera* in the Mediterranean. *J. Mar. Biol. Ass. U. K.* **1991** 71(1), 47-51. (doi:10.1017/S0025315400037383)
253. Deheyn, D.D.; Wilson N.G. Bioluminescent signals spatially amplified by wavelength-specific diffusion through the shell of a marine snail. *Proc. Roy. Soc. B.* **2011** 278(1715), 2112-21. (doi:10.1098/rspb.2010.2203)
254. Houbrick, R.S. Anatomy, reproductive biology, and phylogeny of the Planaxidae (Cerithiacea: Prosobranchia). *Smithson. Contrib. Zool.* **1987** 445, 1 – 58. (doi:10.5479/SI.00810282.445)
255. Haneda, Y. Studies on luminescence in marine snails. *Pac. Sci.* **1958** 12, 152 – 157.
256. Marshall, B.A. A luminescent eulimid (Mollusca: Gastropoda) from New Zealand. *Molluscan Res.* **1997** 18(1), 69-72. (doi:10.1080/13235818.1997.10673683)
257. Nicol, J.A.C. (1964). Special effectors: luminous organs, chromatophores, pigments, and poison gland. In *Physiology of mollusca* (volume 1); Wilbur, K.M.; Yonge, C.M., Eds.; Academic Press, London, UK, 1964; pp.353-381.
258. OPK Opistobranquis. Available online: https://opistobranquis.info/en/guia/nudibranchia/cladobranchia/unassigned-cladobranchia/phylliroe-lichtensteinii/ (accessed on 15 January 2024)
259. Youtube. Available online: https://www.youtube.com/watch?v=rd75tEQV8-U (accessed on 15 January 2024)
260. Gosliner, T.M.; Vallès, Y. Shedding light onto the genera (Mollusca: Nudibranchia) *Kaloplocamus* and *Plocamopherus* with description of new species belonging to these unique bioluminescent dorids. *Veliger* **2006** 48(3), 178-205
261. Alcock, A. A Naturalist in Indian Seas: Or, Four Years with the Royal Indian Marine Survey Ship Investigator; John Murray: London, UK, 1902; pp. 1-328.
262. King, P.E. Pycnogonids. Hutchinson: London, UK, 1973; pp. 1-144.
263. Herring, P.J. Bioluminescence in the Crustacea. *J. Crust. Biol.* **1985** 5(4), 557-573. (doi:10.1007/BF00026304)

264. Kiernik, E. Über einige bisher unbekannte leuchtende Tiere. *Zool. Anz.* **1908** 33, 376-380.
265. Herring, P.J. Copepod luminescence. *Hydrobiologia* **1988** 167(1), 183-195. (doi:10.1007/BF00026304)
266. Takenaka, Y.; Yamaguchi, A.; Tsuruoka, N.; Torimura, M.; Gojobori, T.; Shigeri, Y. Evolution of bioluminescence in marine planktonic copepods. *Mol. Biol. Evol.* **2012** 29(6), 1669-1681. (doi:10.1093/molbev/mss009)
267. Tett, P.B.; Kelly, M.G. Marine bioluminescence. *Oceanogr. Mar. Biol. Annu. Rev.* **1973** 11, 89-173.
268. Artiomkin, A.S., Filimonov, V.S.; Baldina, E.P.; Grese, V.N. Resultados preliminares sobre zooplancton y su luminiscencia en la region oriental del mar Caribe. *Acad. Cienc. Cuba Ser. Oceanol.* **1969** 2, 1- 11.
269. Rudyakov, Y.A.; Voronina, N.M. Plankton and bioluminescence in the Red Sea and the Gulf of Aden. *Oceanology* **1967** 7, 838-848.
270. Filimonov, V.S.; Chumakova, R.I. Biophysical characteristics of the luminescence of marine bioluminescent organisms. In Bioluminescence of the Sea; Gitelson, I.I., (ed.); Nauka: Moscow, Russia, 1969; pp. 40-67.
271. Krylov, C.A. Glandular topography and luminescence of *Metridia pacifica*. *Izv. sib. Otdel. Akad. Nauk SSSR* **1969** 3, 104-109.
272. Lapota, D.; Losee, J.R. Observations of bioluminescence in marine plankton from the Sea of Cortez. *J. Exp. Mar. Biol. Ecol.* **1984** 77(3), 209-239. (doi:10.1016/0022-0981(84)90121-7)
273. Hardy, A.C. The open sea. Its Natural History: The world of Plankton. Collins : London, UK, 1956, pp. 1-335.
274. Chihara, M.; Murano, M. An illustrated guide to marine plankton in Japan. Tokai University Press: Tokyo, Japan, 1997; pp. 1-1574.
275. Neilson, D.J.; Latz, M.I.; Case, J.F. Temporal variability in the vertical structure of bioluminescence in the North Atlantic Ocean. *J. Geophys. Res. Oceans* **1995** 100(C4), 6591-6603. (doi:10.1029/94JC01448)
276. Herring, P.J.; Latz, M.I.; Bannister, N.J.; Widder, E.A. Bioluminescence of the poecilostomatoid copepod *Oncaea conifera*. *Mar. Ecol. Prog. Ser.* **1993** 94, 297-309.
277. Takenaka, Y.; Noda-Ogura, A.; Imanishi, T.; Yamaguchi, A.; Gojobori, T.; Shigeri, Y. Computational analysis and functional expression of ancestral copepod luciferase. *Gene* **2013** 528, 201– 206. (doi:10.1016/j.gene.2013.07.011)
278. Markova, S.V.; Golz, S.; Frank, L.A.; Kalthof, B.; Vysotski, E.S. Cloning and expression of cDNA for a luciferase from the marine copepod *Metridia longa*. A novel secreted bioluminescent reporter enzyme. *J. Biol. Chem.* **2004** 279, 3212–3217. (doi:10.1074/jbc.M309639200)
279. David, C.N.; Conover, R.J. Preliminary investigation on the physiology and ecology of luminescence in the copepod, *Metridia lucens*. *Biol. Bull.* **1961** 121(1), 92-107. (doi:10.2307/1539462)
280. Bityukov, E.P; Evstigneev, P.V. Main characteristics of luminescence and its species specificity in copepods of genus *Pleuromamma. Ekol. Morya* **1982** 11, 53-62.
281. Latz, M.I.; Frank, T.M.; Bowlby, M.R.; Widder, E.A.; Case, J.F. Variability in flash characteristics of a bioluminescent copepod. *Biol. Bull.* **1987** 173(3), 489-503. (doi:10.2307/1541695)
282. Losee, J.R., Lapota, D.; Lieberman, S.H. Bioluminescence: a new tool for oceanography. In (ed.) A. Zirino Mapping strategies in chemical oceanography; Zirino, Z., Ed.; American Chemical Society: Washington, USA, 1985; pp: 211-234.
283. Morin, J.G. Luminaries of the reef: The history of luminescent ostracods and their courtship displays in the Caribbean. *J. Crustac. Biol.* **2019** 39(3), 227-243. (doi:10.1093/jcbiol/ruz009)
284. Cohen, A.C.; Morin, J.G. Two new bioluminescent ostracode genera, *Enewton* and *Photeros* (Myodocopida: Cypridinidae), with three new species from Jamaica. *J. Crustac. Biol.* **2010** 30(1), 1-55. (doi:10.1651/08-3075.1)
285. Cohen, A.C.; Morin, J.G. The cypridinid copulatory limb and a new genus *Kornickeria* (Ostracoda: Myodocopida) with four new species of bioluminescent ostracods from the Caribbean. *Zool. J. Linn. Soc.* **1993** 108(1), 23-84. (doi:10.1111/J.1096-3642.1993.TB02559B.X)
286. Reda, N.J.; Morin, J.G.; Torres, E.; Cohen, A.C.; Schawaroch, V.; Gerrish, G.A. *Maristella*, a new bioluminescent ostracod genus in the Myodocopida (Cypridinidae). *Zool. J. Linn. Soc.* **2019** 187(4), 1078-1118. (doi:10.1093/zoolinnean/zlz082)
287. Torres, E.; Morin, J.G. *Vargula annecohenae*, a new species of bioluminescent ostracode (Myodocopida: Cypridinidae) from Belize. *J. Crustac. Biol.* **2007** 27(4), 649-659. (doi:10.1651/S-2769.1)
288. Huvard, A.L. Analysis of visual pigment absorbance and luminescence emission spectra in marine ostracodes (Crustacea: Ostracoda). *Comp. Biochem. Physiol. A Mol. Integr. Physiol.* **1993** 104(2), 333-338.
289. Torres, E.; Cohen, A.C. (2005). *Vargula morini*, a new species of bioluminescent ostracode (Myodocopida: Cypridinidae) from Belize and an associated copepod (Copepoda: Siphonostomatoida: Nicothoidae). *J. Crustac. Biol.* 25(1), 11-24. (doi:10.1651/C-2455)
290. Parker, A.R. A new species of Australian bioluminescent ostracod (Myodocopina: Cypridinidae) with an unreported iridescent structure. *J. Nat. Hist.* **1998** 32 (5), 645-660. (doi:10.1080/00222939800770341)

291. Cohen, A.C.; Morin, J.G. Three new luminescent ostracodes of the genus *Vargula* (Myodocopida, Cypridininae) from the San Blas Region of Panama. *Contrib. Sci.* **1986** 373, 1-23.
292. Morin, J.G.; Cohen, A.C. Two new luminescent ostracodes of the genus *Vargula* (Myodocopida: Cypridinidae) from the San Blas region of Panama. *J. Crustac. Biol.* **1988** 8, 620 – 638. (doi:10.1163/193724088X00468)
293. Angel, M.V. Bioluminescence in planktonic halocyprid ostracods. *J. Mar. Biol. Assoc. U. K.* **1968** 48(1), 255-257. (doi:10.1017/S0025315400032562)
294. Rudjakov, J.A. The study of the luminescence of pelagic ostracods. In Bioenergetics and biological spectrophotometry; Nauka: Moscow, Russia, 1967; pp. 52-62.
295. Campbell, A.K.; Herring, P.J. Imidazolopyrazine bioluminescence in copepods and other marine organisms. *Mar. Biol.* **1990** 104, 219-225. (doi:10.1007/BF01313261)
296. Oba, Y.; Tsuduki, H.; Kato, S.I.; Ojika, M.; Inouye, S. Identification of the luciferin – luciferase system and quantification of coelenterazine by mass spectrometry in the deep-sea luminous ostracod *Conchoecia pseudodiscophora. ChemBioChem* **2004** 5(11), 1495-1499. (doi:10.1002/cbic.200400102)
297. Batchelder, H.P.; Swift, E. Bioluminescent potential and variability in some Sargasso Sea planktonic halocyprid ostracods. *J. Crustac. Biol.* **1988** 8(4), 520-523. (doi:10.1163/193724088X00350)
298. Angel, M.V. Planktonic oceanic ostracods—historical, present and future. *P. Roy. Soc. Edinb. B.* **1972** 73, 213-228. (doi:10.1017/S0080455X00002277)
299. Herring, P.J. Studies on bioluminescent marine amphipods. *J. Mar. Biol. Assoc. U. K.* **1981** 61(1), 161-176. (doi:10.1017/S0025315400045999)
300. Bowlby, M.R.; Widder, E.A.; Case, J.F. Disparate forms of bioluminescence from the amphipods *Cyphocaris faurei*, *Scina crassicornis* and *S. borealis*. *Mar. Biol.* **1991** 108(2), 247-253. (doi:10.1007/BF01344339)
301. Bowman, T. Bioluminescence in Two Species of Pelagic Amphipods. *J. Fish. Res. Board Can.* **1967** 24, 687 – 688. (doi:10.1139/f67-059)
302. Petrus, J.L. Bioluminiscencia en *Gammarus aequicauda* (Crustacea, Amphipoda) y *Chaetomorpha crassa* (Chlorophyceae) de la laguna costera de la Albufera des Grau (Menorca). *Boll. Soc. Hist. Nat. Balears* **1993** 36, 41 – 44.
303. Woltereck, R. *Scypholanceola*, eine neue hyperidengattung mit reflektororgan. *Zool. Anz.* **1905** 29, 413–416.
304. Herring, P.J. Luminescence in Marine Amphipods. *Nature* **1967** 1260 – 1261. (doi:10.1038/2141260a0)
305. Van Vollenhoven, S. De Dieren van Nederland. Natuurlijke Historie van Nederland (Volume 1); A. C. Kruseman: Haarlem, The Netherlands, 1860; 296 pp. 1-296.
306. Wittmann, K.J. The Petalophthalmidae (Crustacea: Mysida) of the ANDEEP I–III expeditions to the Antarctic deep sea, with description of a new species and first record of photophores in mysids. *Crustac. Res.* **2022** 51(0), 55-89. (doi:10.18353/crustacea.51.0_55)
307. Meland, K.; Aas, P.Ø. A taxonomical review of the *Gnathophausia* (Crustacea, Lophogastrida), with new records from the northern mid-Atlantic ridge. *Zootaxa* **2013** 3664, 199-225. (doi: 10.11646/zootaxa.3664.2.5)
308. Shimomura, O.; Inoue, S.; Johnson, F.H.; Haneda, Y. Widespread occurrence of coelenterazine in marine bioluminescence. *Comp. Biochem. Physiol. B, Biochem. Mol. Biol.* **1980** 65(2), 435-437. (doi:10.1016/0305-0491(80)90044-9)
309. Vereshchaka, A.L.; Kulagin, D.N.; Lunina, A.A. A phylogenetic study of krill (Crustacea: Euphausiacea) reveals new taxa and co-evolution of morphological characters. *Cladistics* **2019** 35(2), 150-172 (doi:10.1111/cla.12239)
310. Tsuji, F.I.; Haneda, Y.; Lynch III, R.V.; Sugiyama, N. Luminescence cross-reactions of *Porichthys* luciferin and theories on the origin of luciferin in some shallow-water fishes. *Comp. Biochem. Physiol.* **1971** 40(1), 163-179. (doi:10.1016/0300-9629(71)90159-9)
311. Swift, E.; Biggley, W.H.; Napora, T.A. The bioluminescence emission spectra of *Pyrosoma atlanticum*, *P. spinosum* (Tunicata), *Euphausia tenera* (Crustacea) and *Gonostoma sp.* (Pisces). *J. Mar. Biol. Assoc. U. K.* **1977** 57(3), 817-823. (doi:10.1017/S0025315400025170)
312. Herring, P.J.; Locket, N.A. The luminescence and photophores of euphausiid crustaceans. *J. Zool.* **1978** 186(4), 431-462. (doi:10.1111/j.1469-7998.1978.tb03932.x)
313. Hansen, H.J. The euphausiacean crustaceans of the "Albatross" expedition to the Philippines. *Proc. U. S. Natl. Mus.* **1916** 49(2129), 635-654.
314. Calman, W.T. On deep-sea Crustacea from the south west of Ireland. *Trans. Roy. Irish Acad.* **1896** 31, 1-22.
315. Sars, G.O. Preliminary notices on the Schizopoda of H.M.S. Challenger Expedition. *Forh. Vidensk.-Selsk. Kristiania* **1883** 7, 1-43.
316. Cram, D.L.,; Malan, O.G. On the possibility of surveying krill (*Euphausia superba* Dana) in the Southern Ocean by remote sensing. *S. Afr. J. Antarct. Res.* **1977** 7, 1–6.
317. Guglielmo, L.; Granata, A.; Guglielmo, R. Orden euphausiacea. *Ibero Divers. Etimol.* **2015** 86A, 1–20.

318. Kampa, E.M.; Boden, B.P. Light generation in a sonic-scattering layer. *Deep Sea Res.* **1957** 4, 73-92. (doi:10.1016/0146-6313(56)90038-7)
319. Brinton, E. A new abyssal euphausiid, *Thysanopoda minyops*, with comparisons of eye size, photophores, and associated structures among deep-living species. *J. Crustac. Biol.* **1987** 7(4), 636-666. (doi:10.1163/193724087X00405)
320. Herring, P.J. Bioluminescence in decapod Crustacea. *J. Mar. Biol. Assoc. U. K.* **1976** 56(4), 1029-1047. (doi:10.1017/S0025315400021056)
321. Wong, J.M.; Pérez-Moreno, J.L.; Chan, T.Y.; Frank, T.M.; Bracken-Grissom, H.D. Phylogenetic and transcriptomic analyses reveal the evolution of bioluminescence and light detection in marine deep-sea shrimps of the family Oplophoridae (Crustacea: Decapoda). *Mol. Phylogenetics Evol.* **2015** 83, 278-292. (doi:10.1016/j.ympev.2014.11.013)
322. Frank, T.M.; Case, J.F. Visual spectral sensitivities of bioluminescent deep-sea crustaceans. *Biol. Bull.* **1988** 175(2), 261-273. (doi:10.2307/1541567)
323. Dennell, R. Observations on the luminescence of bathypelagic Crustacea Decapoda of the Bermuda area. *Zool. J. Linn. Soc.* **1955** 42, 393-406. (doi:10.1111/j.1096-3642.1955.tb02215.x)
324. Crosnier, A. Crustaces Decapodes, Peneides Aristeidae (Benthesicyminae, Aristeinae, Solenocerinae). *Faune Madag.* **1978** 46, 1-197.
325. Dall, W. Australian species of Aristeidae and Benthesicymidae (Penaeoidea: Decapoda). *Mem. Queensl. Mus.* **2001** 46(2), 409–441.
326. Kemp, S.W. The Decapoda Natantia of the coasts of Ireland. *Fisheries Ireland Sci. Invest.* **1908** 1, 1–190.
327. Biggley, W.H.; Napora, T.A.; Swift, E. The color of bioluminescent secretions from decapod prawns in the genera *Oplophorus* and *Systellaspis*. In Bioluminescence Current Perspectives; Nealson, K.H., Ed.; Burgess Publishing Co., Minneapolis, USA, 1981; pp. 66-71.
328. Johnson, F.H.; Stachel, H.D.; Shimomura, O.; Haneda, Y. Partial purification of luminescence system of a deep-sea shrimp, *Hoplophorus gracilorostris*. In *Bioluminescence in Progress*; Johnson, F.; Haneda, Y., Eds.; Princeton University Press, Princeton, USA, 1966; pp. 523-532.
329. Yaldwyn, J.C.J. Deep-sea prawns - their colour and luminescence. *Aust. Nat. Hist.* **1963** 14, 149-152.
330. Inoue, S.; Kakoi, H. ; Goto, T. *Oplophorus* luciferin, bioluminescent substance of the decapod shrimps, *Oplophorus spinosus* and *Heterocarpus laevigatus*. *J. Chem. Soc., Chem. Commun.* **1976** (24), 1056-1057.
331. Vourey, E. Au large de la Nouvelle-Calédonie - Découverte du micronecton (poster presentation). Communauté du Pacifique (Projet BIOPELAGOS), 2019.
332. Balss, H. Decapoda Leuchtorgane. In *Bronn's Klassen und Ordnung des Tierreichs* (Volume 5), C.F. Winter: Leipzig, Germany, 1944; pp. 661-667.
333. Frank, T.M.; Johnsen, S.; Bracken-Grissom, H.; Messing, C.G.; Widder, E. Vision and Bioluminescence in the Deep-sea Benthos (abstract #ME34A-0789. American Geophysical Union, Ocean Sciences Meeting, New Orleans, USA, 21-26 February 2016.
334. Herring, P.J.; Barnes, A.T. Light-stimulated bioluminescence of *Thalassocaris crinita* (Dana)(Decapoda, Caridea). *Crustaceana* **1976** 31(1), 107-110. (doi:10.1163/156854076X00864)
335. Gopala, M.; Williamson, D. I. Decapod Crustacea from the International Indian Ocean Expedition. The species of *Thalassocaris* (Caridea) and their larvae. *J. Zool.* **1971** 165, 27-51. (doi:10.1111/j.1469-7998.1971.tb02175.x)
336. Oceanographic expeditions. IX. The bathypelagic caridean Crustacea. *Zoologica N. Y.* **1940** 25, 117-209.
337. Vereshchaka, A.L.; Olesen, J.; Lunina, A.A. Global diversity and phylogeny of pelagic shrimps of the former genera *Sergestes* and *Sergia* (Crustacea, Dendrobranchiata, Sergestidae), with definition of eight new genera. *PloS One* **2014** 9(11), e112057. (doi:10.1371/journal.pone.0112057)
338. Thomson, C.M.; Herring, P.J.; Campbell, A.K. Coelenterazine distribution and luciferase characteristics in oceanic decapod crustaceans. *Mar. Biol.* **1995** 124, 197-207. (doi:10.1007/bf00347123)
339. Mencher, F.M. Aspects of countershading in Hawaiian mesopelagic sergestid shrimp. Masters Thesis, Univ. of Hawaii., Honolulu, USA, December 1978.
340. Warner, J.A.; Latz, M.I.; Case, J.F. Cryptic bioluminescence in a midwater shrimp. *Science* **1979** 203(4385), 1109-1110. (doi:10.1126/science.203.4385.1109)
341. Hansen, H.J. Crustacés Décapodes (Sergestides) provenant des campagnes des yachts Hirondelle et Princesse Alice (1885-1915). *Résultats des Campagnes scientifiques accomplies par le Prince Albert I de Monaco* **1922** 64, 1-232.
342. Farfante, I.P. American solenocerid shrimps of the genera *Hymenopenaeus*, *Haliporoides*, *Pleoticus*, *Hadropenaeus* new genus, *Andmesopenaeus* new genus. *Fish. Bull.* **1977** 75(2), 261-346.
343. Burkenroad, M.D. The Aristaeinae, Solenocerinae and pelagic Penaeinae of the Bingham Oceanographic collection. *Bull. Bingham Oceanogr. Collect.* **1936** 5, 1-151.

344. Delage Y., Hérouard E. Les procordés. In *Traité de Zoologie Concrète* (Volume VIII); Schleicher Frères: Paris, France, 1898; pp. 3-64.
345. Cormier, M.J.; Dure, L.S. Studies on the bioluminescence of *Balanoglossus biminiensis* extracts: I. Requirement for hydrogen peroxide and characteristics of the system. *J. Biol. Chem.* **1963** 238(2), 785-789. (doi: 10.1016/S0021-9258(18)81335-7)
346. Kuwano, H. New enteropneust from Misaki. *Anttot. Zool. Jap.* **1902** 4, 77-84.
347. Panceri, P. La luce e gli organi luminosi di alcuni annelidi. *Atti. Accad. Sci. Napoli* **1875** 7, 1–20.
348. Harvey, E.N. On the inhibition of animal luminescence by light. *Biol. Bull.* **1926** 51, 85-8.
349. Kanakubo, A.; Koga, K.; Isobe, M.; Yoza, K. Tetrabromohydroquinone and riboflavin are possibly responsible for green luminescence in the luminous acorn worm, *Ptychodera flava*. *Luminescence* **2005** 20(6), 397-400. (doi:10.1002/bio.803)
350. Herring, P.J. New observations on the bioluminescence of echinoderms. *J. Zool.* **1974** 172(3), 401-418. (doi:10.1111/j.1469-7998.1974.tb04116.x)
351. Mallefet, J.; Martinez-Soares, P.; Eléaume, M.; O'hara, T.; Duchatelet, L. New insights on crinoid (Echinodermata; Crinoidea) bioluminescence. *Front. Mar. Sci.* **2023** 10, 1136138. (doi:10.3389/fmars.2023.1136138)
352. Dilly, P.N. Enigma of coloration and light emission in deep-sea animals. *Endeavour* **1973** 32 (115), 25–29.
353. Bessho-Uehara, M.; Mallefet, J.; Haddock, S.H. Glowing sea cucumbers: Bioluminescence in the Holothuroidea. In *The world of sea cucumbers*; Mercier, A.; Hamel J.F.; Suhrbier A.D.; Pearce, C.M., Eds.; Academic Press: London, UK, 2024; pp. 361-375.
354. Robison, B.H. Bioluminescence in the benthopelagic holothurian *Enypniastes eximia*. *J. Mar. Biol. Assoc. U. K.* **1992** 72(2), 463-472. (doi:10.1017/S0025315400037826)
355. Bell, A.C. Histology and ultrastructure of *Acrocnida brachiata*. PhD thesis, Queen's University Belfast, Belfast, UK, 1974.
356. Mallefet, J.; Vanhoutte, P.; Baguet, F. Study of *Amphipholis squamata* luminescence. In *Echinoderm Research* ; CRC Press: Boca Raton, USA, 1991; pp. 125-130.
357. Mallefet, J. (2009). "Echinoderm bioluminescence: why, when, and how do so many ophiuroids glow?," in Bioluminescence in Focus: a Collection of Illuminating Essays, ed. V. B. Rochow (Kerala: Research Signpost), 67–84.
358. Mallefet, J. Echinoderm bioluminescence: why, when, and how do so many ophiuroids glow? In *Bioluminescence in focus—a collection of illuminating essays*; Meyer Rochow, V.B., Ed.; Research Signpost, Trivandrum, India, 2009; pp. 67–83.
359. Mallefet, J.; Barker, M.; Byrne, M.; O'Hara, T.; Heinzeller, T.; Nebelsick, J.H. First study of bioluminescence in *Ophionereis*. In *Proceedings of the 11th International Echinoderm Conference*, Munich, Germany, 6-10 October 2003.
360. Mallefet, J.; Fujita, T. First luminescence survey of Okinawa brittle stars. *Luminescence* **2014** 29, 29-30.
361. Dewael, Y.; Mallefet, J. Calcium involvement in the luminescence control of three ophiuroid species (Echinodermata). *Comp. Biochem. Physiol. C Toxicol. Pharmacol.* **2002** 131(2), 153-160. (doi:10.1016/s1532-0456(01)00288-5)
362. Kato, K. On a luminous ophiuroid. *Zool. Mag. Tokyo* **1947** 57, 10.
363. Pentreath, R.J. Feeding mechanisms and the functional morphology of podia and spines in some New Zealand ophiuroids (Echinodermata). *J. Zool.* **1970** 161(3), 395-429. (doi:10.1111/j.1469-7998.1970.tb04520.x)
364. Brehm, P.H. Bioluminescence. The anatomy and physiology of its nervous control in *Ophiopsila californica*. PhD Thesis, University of California, Berkeley, USA, 1975.
365. Guille, A.; Laboute, P. ; Menou, J.L. Guide des étoiles de mer, oursins et autres échinodermes du lagon de Nouvelle-Calédonie (Vol. 25); Editions de Forston, Pris, France, 1986; pp. 1-239.
366. Woolsey, E.; Byrne, M.; Webster, J.M.; Williams, S.; Pizarro, O.; Thornborough, K.; Bridge, T. *Ophiopsila pantherina* beds on subaqueous dunes off the Great Barrier Reef. In *Echinoderms in a Changing World: Proceedings of the 13th International Echinoderm Conference*, Hobart, Australia, 5-9 January 2009.
367. Okanishi, M.; Fujii, T. A new record of brittle star *Ophiopsila cf. polyacantha* (Echinodermata: Ophiuroidea) from Southwestern Japan, with notes on its bioluminescence. *Species Divers.* **2020** 25(2), 283-294. (doi:10.12782/specdiv.25.283)
368. Grober, M.S. Responses of tropical reef fauna to brittle-star luminescence (Echinodermata: Ophiuroidea). *J. Exp. Mar. Biol. Ecol.* **1988** 115(2), 157-168. (doi:10.1016/0022-0981(88)90100-1)
369. Okanishi, M.; Oba, Y.; Fujita, Y. Brittle stars from a submarine cave of Christmas Island, northwestern Australia, with description of a new bioluminescent species *Ophiopsila xmasilluminans* (Echinodermata: Ophiuroidea) and notes on its behaviour. *Raffles Bull. Zool.* **2019** 67, 421-439. (doi:10.26107/RBZ-2019-0034)

370. Jones, A.; Mallefet J. Study of the luminescence in the black brittle-star *Ophiocomina nigra*: toward a new pattern of light emission in ophiuroids. *Zoosymposia* **2012** 7, 139–145. (doi: 10.11646/zoosymposia.7.1.13)
371. Galt, C.P.; Grober, M.S.; Sykes, P. F. Taxonomic correlates of bioluminescence among appendicularians (Urochordata: Larvacea). *Biol. Bull.* **1985** 168(1), 125-134. (doi:10.2307/1541178)
372. Lohmann, H. Das Gehäuse der Appendicularien nach seiner Bildungsweise, seinem Bau und seiner Funktion. *Zool. Anz.* **1899** 22(587), 206-214.
373. Flood, P.R. A new appendicularian, *Oikopleura gorskyi* n. sp. (Tunicata), from Norwegian fjords. *Beaufortia* **2000** 50, 69-77.
374. Galt, C.P.; Sykes, P.F. Sites of bioluminescence in the appendicularians *Oikopleura dioica* and *O. labradoriensis* (Urochordata: Larvacea). *Mar. Biol.* **1983** 77, 155-159. (doi:10.1007/BF00396313)
375. Tarasov, N.I. Luminescence of the sea. Academy of Sciences of the USSR: Moscow, Russia, 1956; pp.1-203.
376. Aoki, M.; Hashimoto, K.; Watanabe, H. The intrinsic origin of bioluminescence in the ascidian, *Clavelina miniata*. *Biol. Bull.* **1989** 176(1), 57-62. (doi:10.2307/1541889)
377. Robison, B.H.; Raskoff, K.A.; Sherlock, R.E. Ecological substrate in midwater: *Doliolula equus*, a new mesopelagic tunicate. *J. Mar. Biol. Assoc. U. K.* **2005** 85 (3), 655-663. (doi:10.1017/S0025315405011586)
378. Tessler, M.; Gaffney, J.P.; Oliveira, A.G.; Guarnaccia, A.; Dobi, K.C.; Gujarati, N.A.; Galbraith, M.; Mirza, J.D.; Sparks, J.S.; Pieribone, V.A.; Wood, R.J.; Gruber, D.F. (2020). A putative chordate luciferase from a cosmopolitan tunicate indicates convergent bioluminescence evolution across phyla. *Sci. Rep.* **2020** 10(1), 1-11. (doi:10.1038/s41598-020-73446-w)
379. van Soest, R. Juvenile colonies of the genus *Pyrostremma* Garstang, 1929 (Tunicata, Thaliacea). *Bull Zool. Mus*. **1974** 4(4), 23-28.
380. van Soest, R. Taxonomy of the subfamily Cyclosalpinae Yount, 1954 (Tunicata, Thaliacea), with descriptions of two new species. *Beaufortia* **1974** 22(288), 17-55.
381. van Soest, R.W.M. Observations on taxonomy and distribution of some salps (Tunicata, Thaliacea), with descriptions of three new species. *Beaufortia* **1975** 23(302): 105-130.
382. Haneda, Y.; Tokioka, T. Droplets from the Plankton net. *Publ. Seto Mar. Biol. Lab.* **1954** 3(3), 369-371.
383. Mallefet, J.; Stevens, D.W.; Duchatelet, L. Bioluminescence of the largest luminous vertebrate, the kitefin shark, *Dalatias licha*: first insights and comparative aspects. *Front. Mar. Sci.* **2021** 8, 633582. (doi:10.3389/fmars.2021.633582)
384. de Figueiredo Petean, F.; de Carvalho, M.R. Comparative morphology and systematics of the cookiecutter sharks, genus *Isistius* Gill (1864)(Chondrichthyes: Squaliformes: Dalatiidae). *PLoS One* **2018** 13(8), e0201913. (doi:10.1371/journal.pone.0201913)
385. Claes, J.M.; Delroisse, J.; Grace, M.A.; Doosey, M.H.; Duchatelet, L.; Mallefet, J. Histological evidence for secretory bioluminescence from pectoral pockets of the American Pocket Shark (*Mollisquama mississippiensis*). *Sci. Rep.* **2020** 10(1), 18762. (doi:10.1038/s41598-020-75656-8)
386. Dolganov, V.N. A new shark from the family Squalidae caught on the Naska submarine ridge. *Zool. Zhurnal* **1984** 63(10), 1589-1591.
387. Shirai, S.; Nakaya, K. A new squalid species of the genus *Centroscyllium* from the Emperor seamont chain. *Jpn. J. Ichtyol.* **1990** 36, 391–398. (doi:10.11369/jji1950.36.391)
388. Burckhardt, R. On the luminous organs of selachian fishes. *Ann. Mag. Nat. Hist.* **1900** 7, 558–568. (doi:10.1080/00222930008678424)
389. Abe, T. Description of a new squaloid shark, *Centroscyllium kamoharai*, from Japan. *Jpn. J. Ichthyol.* **1966** 13, 190–198. (doi:10.11369/jji1950.13.190)
390. Iwai, T. Luminous organs of the deep-sea squaloid shark, *Centroscyllium ritteri* Jordan and Fowler. *Pac. Sci.* **1960** 14, 51–54.
391. Ebert, D.A.; Straube, N.; Leslie, R.W.; Weigmann, S. *Etmopterus alphus* n. sp.: a new lanternshark (Squaliformes: Etmopteridae) from the south-western Indian Ocean. *Afr. J. Mar. Sci.* **2016** 38(3), 329-340. (doi:10.2989/1814232X.2016.1198275)
392. Vásquez, V.E.; Ebert, D.A.; Long, D.J. *Etmopterus benchleyi* n. sp., a new lanternshark (Squaliformes: Etmopteridae) from the central eastern Pacific Ocean. *J. Ocean. Sci. Found.* **2015** 17, 43–55.
393. Castro, J.I. The sharks of north America. Oxford University Press, Oxford, UK, 2010; pp. 1-640.
394. Ebert, D.A.; Leslie, R.W.; Weigmann, S. *Etmopterus brosei* sp. nov.: a new lanternshark (Squaliformes: Etmopteridae) from the southeastern Atlantic and southwestern Indian oceans, with a revised key to the *Etmopterus lucifer* clade. *Marine Biodivers.* **2021** 51(3), 53. (doi:10.1007/s12526-021-01173-0)
395. Reif, W.E. Functions of scales and photophores in mesopelagic luminescent sharks. *Acta Zool.* **1985** 66(2), 111-118. (doi:10.1111/j.1463-6395.1985.tb00829.x)

396. Schaaf-DaSilva, J.A.; Ebert, D.A. *Etmopterus burgessi* sp. nov., a new species of lanternshark (Squaliformes: Etmopteridae) from Taiwan. *Zootaxa* **2006** 1373, 53-64. (doi:10.11646/ZOOTAXA.1373.1.3)
397. Last, P.R.; Burgess, G.H.; Séret, B. Description of six new species of lantern-sharks of the genus *Etmopterus* (Squaloidea: Etmopteridae) from the Australasian region. *Cybium* **2002** 26, 203–223.
398. Fricke, R.; Koch, I. A New Species of the Lantern Shark Genus *Etmopterus* from Southern Africa (Elasmobranchii: Squalidae). *Stutt. Beitr. Naturk.* **1990** 450, 1-9.
399. Chan, W.L. New sharks from the South China Sea. *J. Zool.* **1966** 148, 218–237. (doi:10.1111/j.1469-7998.1966.tb02949.x)
400. Knuckey, J.D.S.; Ebert, D.A.; Burgess, G.H. *Etmopterus joungi* n. sp., a new species of lanternshark (Squaliformes: Etmopteridae) from Taiwan. *Aqua Int. J. Ichthyol.* **2010** 17, 61–72.
401. Ebert, D.A.; Papastamatiou, Y.P.; Kajiura, S.M.; Wetherbee, B.M. *Etmopterus lailae* sp. nov., a new lanternshark (Squaliformes: Etmopteridae) from the Northwestern Hawaiian Islands. *Zootaxa* **2017** 4237, 371–382.
402. Straube, N.; Iglésias, S.P.; Sellos, D.Y.; Kriwet, J.; Schliewen, U.K. Molecular phylogeny and node time estimation of bioluminescent lantern sharks (Elasmobranchii: Etmopteridae). *Mol. Phylogenet. Evol.* **2010** 56, 905–917. (doi:10.1016/j.ympev.2010.04.042)
403. Ebert, D.A.; Van Hees, K.E. *Etmopterus marshae* sp. nov., a new lanternshark (Squaliformes: Etmopteridae) from the Philippine Islands, with a revised key to the *Etmopterus lucifer* clade. *Zootaxa* **2018** 4508, 197–210. (doi:10.11646/zootaxa.4508.2.3)
404. Claes, J.M.; Mallefet, J. Comparative control of luminescence in sharks: new insights from the slendertail lanternshark (*Etmopterus molleri*). *J. Exp. Mar. Biol. Ecol* **2015** 467, 87-94. (doi: 10.1016/j.jembe.2015.03.008)
405. , V.N.; Balanov, A.A. *Etmopterus parini* sp. n. (Squaliformes: Etmopteridae), a new shark species from the northwestern Pacific Ocean. *Biol. Morya* **2018** 44, 427–430.
406. Oshima, H. Some observations on the luminous organs of fishes. *J. Coll. Sci., Imp. Univ., Tokyo* **1911** 27, 1–25.
407. White, W.T.; Ebert, D.A.; Mana, R.R.; Corrigan, S. *Etmopterus samadiae* n. sp., a new lanternshark (Squaliformes: Etmopteridae) from Papua New Guinea. *Zootaxa* **2017** 4244, 339–354. (doi:10.11646/zootaxa.4244.3.3)
408. Bigelow, H.B.; Schoeder, W.C.; Springer, S. New and little known sharks from the Atlantic and from the Gulf of Mexico. *Bull. Mus. Comp. Zool. Harv. Coll.* **1953** 109, 213–276.
409. Ebert, D.A.; Compagno, L.J.; De Vries, M.J. A new lanternshark (Squaliformes: Etmopteridae: *Etmopterus*) from southern Africa. *Copeia* **2011** 2011(3), 379-384. (doi:10.2307/41261894)
410. Claes, J.M.; Aksnes, D.L.; Mallefet, J. (2010). Phantom hunter of the fjords: camouflage by counterillumination in a shark (*Etmopterus spinax*). *J. Exp. Mar. Biol. Ecol.* **2010** 388(1-2), 28-32. (doi:10.1016/j.jembe.2010.03.009)
411. Dolganov, V.N. On the little-known sharks *Etmopterus villosus* (Etmopteridae) and *Scymnodalatias sherwoodi* (Somniosidae) from the Pacific Ocean. *J. Ichthyol.* **2019**, 59, 275–279. (doi:10.1134/S003294521902005X)
412. Duchatelet, L.; Marion, R.; Mallefet, J. A third luminous shark family: Confirmation of luminescence ability for *Zameus squamulosus* (Squaliformes; Somniosidae). *Photochem. Photobiol.* **2021** 97(4), 739-744. (doi:10.1111/php.13393)
413. Sparks, J.S.; Chakrabarty, P. Description of a New Species of Ponyfish (Teleostei: Leiognathidae: Equulitini: *Photolateralis*) from the Gulf of Oman. *Am. Mus. Novit.* **2019** 3929 1-14. (doi:https://doi.org/10.1206/3929.1)
414. Suzuki, H.; Kimura, S. Taxonomic revision of the *Equulites elongatus* (Günther 1874) species group (Perciformes: Leiognathidae) with the description of a new species. *Ichthyol. Res.* **2017** 64(3): 339-352. (doi:10.1007/s10228-017-0572-9)
415. McFall-Ngai, M.; Morin, J.G. (1991). Camouflage by disruptive illumination in leiognathids, a family of shallow-water, bioluminescent fishes. *J. Exp. Biol.* **1991** 156(1), 119-137. (doi:10.1242/jeb.156.1.119)
416. Haneda, Y. Luminous organs of fish which emit light indirectly. *Pac. Sci.* **1950** 4, 214–227
417. Alavi-Yeganeh, M.S.; Khajavi, M.; Kimura, S. A new ponyfish, *Deveximentum mekranensis* (Teleostei: Leiognathidae), from the Gulf of Oman. *Ichthyol. Res.* **2021** 68 (3), 437-444. (doi:10.1007/s10228-020-00794-y)
418. Okamoto, M.; Williams, J. T.; Carpenter, K. E.; Santos, M. D.; Kimura, S. Description of a new species of the genus *Acropoma* (Acropomatidae) from the Arafura Sea with a redescription of *Acropoma leobergi* Prokofiev 2018 and the first record of *Acropoma boholensis* Yamanoue and Matsuura 2002 from the South China Sea. *Ichthyol. Res.* **2019** 67(1), 39-49. (doi:10.1007/s10228-019-00696-8)
419. Ghedotti, M.J.; Gruber, J.N.; Barton, R.W.; Davis, M.P.; Smith, W.L. Morphology and evolution of bioluminescent organs in the glowbellies (Percomorpha: Acropomatidae) with comments on the taxonomy and phylogeny of Acropomatiformes. *J. Morphol.* **2018** 279(11), 1640-1653. (doi:10.1002/jmor.20894)
420. Okamoto, M.; Golani, D. Three new species of the genus *Acropoma* (Perciformes: Acropomatidae) from the Indian Ocean. *Ichthyol. Res.* **2017** 65(1), 101-114. (doi:10.1007/s10228-017-0595-2)

421. Prokofiev, A.M. A New Species of Luminous Percoids of the *Acropoma* Genus (Acropomatidae). *J. Ichthyol.* **2018** 58(6), 939-943. (doi:10.1134/S0032945218060152)
422. Okamoto, M.; Randall, J.E.; Motomura, H. *Acropoma musorstom*, a new lanternbelly (Acropomatidae) from the South Pacific and the first record of *Acropoma splendens* from the Andaman Sea off southwestern Thailand. *Ichthyol. Res.* **2021** 68, 517-528. (doi:10.1007/s10228-021-00802-9)
423. Okamoto, M. *Acropoma profundum*, a new species of lanternbelly (Teleostei: Perciformes: Acropomatidae) from the Solomon Islands. *Species Divers.* **2014** 19, 9-14. (doi:10.1007/s10228-021-00802-9)
424. Mayer, G.F. A revision of the cardinalfish genus *Epigonus* (Perciformes, Apogonidae), with descriptions of two new species. *Bull. Mus. Comp. Zool.* **1974** 146,147-203.
425. Idrees babu, K.; Akhilesh, K.V. *Epigonus indicus*, a new species of deepwater cardinalfish (Perciformes: Epigonidae) from the Indian Ocean. *J. Ocean Sci. Found.* **2020** 36, 20-27. (doi:10.5281/zenodo.4243312)
426. Herring, P.J. Bioluminescence of the oceanic apogonid fishes *Howella brodiei* and *Florenciella lugubris*. *J. Mar. Biol. Assoc. U. K.* **1992** 72(1), 139-148. (doi:10.1017/S0025315400048840)
427. Tominaga, Y. Internal morphology, mutual relationships and systematic position of the fishes belonging to the family Pempheridae. *Jpn. J. Ichthyol.* **1968** 15(2), 43-95..
428. Haneda, Y.; Johnson, F.H.; Shimomura, O. (1966). The origin of luciferin in the luminous ducts of *Parapriacanthus ransonneti*, *Pempheris klunzingeri*, and *Apogon ellioti*. In *Bioluminescence in Progress*; Johnson, F.; Haneda, Y., Eds.; Princeton University Press, Princeton, USA, 1966; pp. 533-545.
429. Sazonov, Y.I. Morphology and significance of the luminous organs in alepocephaloid fishes. *Biosyst. Ecol. Ser.* **1996** 11, 151–163.
430. Best, A.C.G.; Bone, Q. On the integument and photophores of the alepocephalid fishes *Xenodermichthys* and *Photostylus*. *J. Mar. Biol. Assoc. U. K.* **1976** 56(1), 227-236. (doi:10.1017/S0025315400020567)
431. Markle, D.F. Taxonomy and distribution of *Rouleina attrita* and *Rouleina maderensis* (Pisces, Alepocephalidae). *Fish. Bull.* **1971** 76, 79-87
432. Sazonov, Y.I. A new species of the genus *Rouleina* (Argentiniformes, Alepocephalidae) from the Philippine Archipelago Seas and a redescription of *R. livida* and *R. nuda*. *J. Ichthyol.* **1999** 39(7), 479-487.
433. Beebe, W. Deep-sea fishes of the Bermuda Oceanographic Expeditions. Family Alepocephalidae. *Zoologica N. Y.* **1933** 16, 15-91.
434. Matsubara, K.; Iwai, T. Notes on the alepocephalid fish *Xenodermichthys nodulosus* Gunther, from Japan. *Bull. Misaki Mar. Biol. Inst.* **1969** 1-10.
435. Poulsen, J.Y. New observations and ontogenetic transformation of photogenic tissues in the tubeshoulder *Sagamichthys schnakenbecki* (Platytroctidae, Alepocephaliformes). *J. Fish Biol.* **2019** 94(1), 62-76. (doi:10.1111/jfb.13857)
436. Sazonov, YI.; Merrett, N.R. Alepocephaloid fishes (Argentiniformes, Alepocephaloidei) captured during the cruise of the R/V Discovery in the Arabian Sea, with description of a new species of *Normichthys* (Platytroctidae). *J. Ichthyol.* **2001** 41(suppl. 1), S37-S50.
437. Tucker, D.W. Report on the fishes collected by S. Y. "Rosaura" in the North and Central Atlantic, 1937–1938. Part 1. Rosaura Expedition Report Number 4, Part 1. *Bull. Br. Mus.* **1954** 2, 163–214.
438. Castle, P.H.J.; Paxton, J.R. A new genus and species of luminescent eel (Pisces: Congridae) from the Arafura Sea, Northern Australia. *Copeia* **1984** 1984(1), 72-81.
439. Beebe, W. Deep-sea fishes of the Bermuda oceanographic expeditions. 1: Family Derichthyidae. 2: Family Nessorhamphidae. *Zoologica N. Y.* **1935** 21, 195-205.
440. Poulsen, J.Y. A new species of pencil smelt *Nansenia boreacrassicauda* (Microstomatidae, Argentiniformes) from the North Atlantic Ocean. *Zootaxa* **2015** 4020(3), 517-532. (doi:10.11646/zootaxa.4020.3.6)
441. Parin, N.V.; Belyanina, T.N.; Evseenko, S.A. Materials to the revision of the genus *Dolichopteryx* and closely related taxa (*Ioichthys*, *Bathylychnops*) with the separation of a new genus *Dolichopteroides* and description of three new species (fam. Opisthoproctidae). *J. Ichthyol.* **2009** 49(10), 839-851. (doi:10.1134/S0032945209100014)
442. Poulsen, J.Y.; Sado, T.; Hahn, C.; Byrkjedal, I.; Moku, M.; Miya, M. Preservation Obscures Pelagic Deep-Sea Fish Diversity: Doubling the Number of Sole-Bearing Opisthoproctids and Resurrection of the Genus *Monacoa* (Opisthoproctidae, Argentiniformes). *Plos One* **2016** 11(8), e0159762. (doi:10.1371/journal.pone.0159762)
443. Prokofiev, A.M.; Kukuev, E.I. A New Species of *Rhynchohyalus* from the Southeastern Pacific Ocean with Notes on *R. natalensis* (Opisthoproctidae). *J. Ichthyol.* **2020** 60(4), 513-519. (doi:10.1134/S0032945220040189)
444. Bertelsen, E.; Munk, O. Rectal light organs in the argentinoid fishes *Opisthoproctus* and *Winteria*. *Dana Rep.* **1964** 62, 1–17.
445. Fricke, R.; Durville, P. *Chlorophthalmus vulcanus*, a new species of greeneye from La Réunion, southwestern Indian Ocean (Teleostei: Chlorophthalmidae). *FishTaxa* **2020** 17, 1-11.

446. Somiya, H. Bacterial bioluminescence in chlorophthalmid deep-sea fish: a possible interrelationship between the light organ and the eyes. *Experientia* **1977** 33(7), 906-909. (doi:10.1007/BF01951274)
447. Prokofiev, A.M. A New Species of Greeneye (Chlorophthalmidae) from the Waters of Vietnam. *J. Ichthyol.* **2020** 60(6): 938-942.
448. Saito, R.; Sakai, T.; Shimizu, T.; Mizumachi, K.; Koizumi, R.; Ono, T.; Sogame, Y. Isolation of *Photobacterium kishitanii taigaleon* from a local fish Mehikari (greeneye) found near Iwaki city Japan, and possible application for water quality assessment. *Asian Jr. of Microbiol. Biotech. Env. Sc.* **2020** 22(4), 584-593.
449. Wassersug, R.J.; Johnson, R.K. A remarkable pyloric caecum in the evermannellid genus *Coccorella* with notes on gut structure and function in alepisauroid fishes (Pisces, Myctophiformes). *J. Zool.* **1976** 179(2), 273-289. (doi:10.1111/j.1469-7998.1976.tb02296.x)
450. Rofen R.R. Family Paralepididae. In *Fishes of Western North Atlantic*, pt. 5(1); Olsen, Y.H.; Atz, J.W., Eds.; Memoir Sears Foundation for Marine Research: New Haven, USA, 1966; pp. 205–461.
451. Graae, M.J.F. *Lestidium bigelowi*, a new species of paralepidid fish with photophores. *Breviora* **1967** 277, 1-10.
452. Ho, H.C.; Graham, K.; Russell, B. Three new species of the barracudina genus *Lestidium* (Aulopiformes: Paralepididae) from the Indo-West Pacific. *Zootaxa* **2020** 4767(1), 71-88. (doi:10.11646/zootaxa.4767.1.3)
453. Gilbert, C.H. The deep-sea fishes of the Hawaiian Islands. *Fish. Bull.* **1905** 23(2), 577-713.
454. Ho, H.C.; Tsai, S.Y.; Li, H.H. The barracudina genera *Lestidium* and *Lestrolepis* of Taiwan, with descriptions of two new species (Aulopiformes: Paralepididae). *Zootaxa* **2019** 4702(1), 114-139. (doi:10.11646/zootaxa.4702.1.16)
455. Ghedotti, M.J.; Barton, R.W. ; Simons, A.M. ; Davis, M.P. The first report of luminescent liver tissue in fishes: Evolution and structure of bioluminescent organs in the deep-sea naked barracudinas (Aulopiformes: Lestidiidae). *J. Morphol.* **2015** 276(3), 310-318. (doi:10.1002/jmor.20341)
456. Ho, H.C.; Golani, D. A new species of *Lestrolepis* from the Red Sea, with redescription of *Lestrolepis pofi* (Harry, 1953) (Aulopiformes: Parelepididae). *Zootaxa* **2019** 4619(3), 571-579. (doi:10.11646/zootaxa.4619.3.10)
457. Johnston, I.A.; Herring, P.J. The transformation of muscle into bioluminescent tissue in the fish *Benthalbella infans* Zugmayer. *Proc. Roy. Soc. B.* **1985** 225(1239), 213-218. (doi:10.1098/rspb.1985.0059)
458. Johnson, R.K. A revision of the alepisauroid family Scopelarchidae (Pisces: Myctophiformes). *Fieldiana Zool.* **1974** 66, 1-249.
459. Walker, H.J.,Jr.; Rosenblatt, R.H. Pacific toadfishes of the genus *Porichthys* (Batrachoididae) with descriptions of three new species. *Copeia* **1988** 1988 (4), 887-904.
460. Gilbert, C. Western Atlantic batrachoidid fishes of the genus *Porichthy*s, including three new species. *Bull. Mar. Sci.* **1968** 18 (3), 671-730.
461. Schwartz, F.J. Atlantic Midshipman, *Porichthys plectrodon*, in North Carolina. *J. North Carolina Acad. Sci.* **2013** 129(3), 111-114. (doi:10.7572/2167-5880-129.3.111)
462. Haneda, Y. A preliminary report on two new luminous fish of Bombay and Hong Kong. *Sci. Rep. Yokosuka City Mus.* **1961** 6, 45-50.
463. Sasaki, K. Phylogeny of the family Sciaenidae, with notes on its zoogeography (Teleostei, Perciformes). *Mem. Fac. Fish., Hokkaido Univ.* **1989** 36(1-2), 1-137.
464. Seah, Y.G.; Hanafi, N.; Maslan, A.G.; Chao, N.L. A new species of *Larimichthys* from Terengganu, east coast of Peninsular Malaysia (Perciformes: Sciaenidae). *Zootaxa* **2015** 3956(2), 271-280. (doi:10.11646/zootaxa.3956.2.7)
465. Trewavas, E. The sciaenid fishes (croakers or drums) of the Indo-West-Pacific. *Trans. Zool. Soc. London* **1977** 33(4): 253-541. (doi:10.1111/j.1096-3642.1977.tb00052.x)
466. Last, P.; Pogonoski, J.J. Revision of the fish family Euclichthyidae (Pisces: Gadiformes) with the description of two new species from the Western Pacific. *Zootaxa* **2020** 4758(2), 231-256. (doi:10.11646/zootaxa.4758.2.2)
467. Okamura, O. Relationships of the Suborder Macrouroidei and related groups, with comments on Merlucciidae and *Steindachneria*. In *Papers on the systematics of gadiform fishes*; Cohen, D. M., Ed.; Natural History Museum of Los Angeles County: Los Angeles, USA, 1989; pp. 129-142.
468. Merrett, N.R. A new species of the deep-sea fish genus *Coryphaenoides gunnerus* (Macrouridae) from the tropical eastern North Atlantic and its relationship with *Coryphaenoides marshalli* Iwamoto, 1970. *J. Fish Biol.* **1983** 22(3), 265-278. (doi:10.1111/j.1095-8649.1983.tb04750.x)
469. Iwamoto, T.; Williams, A. Grenadiers (Pisces, Gadiformes) from the continental slope of western and northwestern Australia. *Proc. Calif. Acad. Sci.* **1999** 51(3): 105-243.
470. Gilbert, C.H.; Hubbs, C.L. (1920). Contributions to the biology of the Philippine Archipelago and adjacent regions. The macrouroid fishes of the Philippine Islands and the East Indies. *Bull. U.S. Natl. Mus.* **1920** 100(1,pt 7), 369-588.
471. Iwamoto, T. Eastern Pacific macrourids of the genus *Coelorinchus* Giorna (Pisces: Gadiformes), with description of a new species from Chile. *Proc. Calif. Acad. Sci.* **1978** 41(12), 307-337.

472. Iwamoto, T.; Golani, D.; Baranes, 1.; Goren, M. Two new grenadiers (Teleostei, Gadiformes, Macrouridae) from the Seychelles and Mascarene Ridge, Western Indian Ocean. *Proc. Calif. Acad. Sci.* **2006** 57(12-24), 433-442.
473. Chiou, M.-L.; Shao, K.-T.; Iwamoto, T. New species of *Caelorinchus* (Macrouridae, Gadiformes, Teleostei) from Taiwan, with a redescription of *Caelorinchus brevirostris* Okamura. *Copeia* **2004** 2004(2), 298-304.
474. Hickling, C.F. A new type of luminescence in fishes, III. The gland in *Coelorhynchus coelorhynchus* Risso. *J. Mar. Biol. Assoc. U. K.* **1931** 17(3), 853-875. (doi:10.1017/S0025315400009346)
475. McCann, C.; McKnight, D.G. The marine fauna of New Zealand: macrourid fishes (Pisces: Gadida). *New Zealand Oceanog. Inst. Mem.* **1980** 61, 1-91.
476. McMillan, P.J.; Paulin, C.D. Description of nine new species of rattails of the genus *Caelorinchus* (Pisces, Macrouridae) from New Zealand. *Copeia* **1993** 1993(3), 819-840.
477. Iwamoto, T.; Merrett, N.R. Pisces Gadiformes: Taxonomy of grenadiers of the New Caledonian region, southwest Pacific. In Résultats des Campagnes MUSORSTOM 18 ; Crosnier, A., Ed.; Mémoires du Muséum national d'Histoire naturelle (Série A, Zoologie. 176); Paris, France, 1997; pp. 473-570.
478. Iwamoto, T., Nakayama, N., Shao, K.T.; Ho, H.C. Synopsis of the grenadier fishes (Gadiformes; Teleostei) of Taiwan. *Proc. Calif. Acad. Sci.* 2015 62, 31-126.
479. Jawad, L.A.; Al-Mamry, J.M. New records of *Coelorinchus flabellispinnis* (Alcock, 1894), *Coryphaenoides macrolophus* (Alcock, 1894) and *Nezumia investigatoris* (Alcock, 1894) (family: Macruridae) from the Arabian Sea coasts of Oman. *J. Appl. Ichthyol.* **2012** 28(2), 287-289. (doi:10.1111/j.1439-0426.2011.01922.x)
480. Iwamoto, T.; Ho, H.-C.; Shao, K.-T. Description of a new *Coelorinchus* (Macrouridae, Gadiformes, Teleostei) from Taiwan, with notable new records of grenadiers from the South China Sea. *Zootaxa* **2009** 2326, 39-50. (doi:10.11646/zootaxa.2326.1.3)
481. Prokofiev, A.M.; Iwamoto, T. A new *Coelorinchus* from the western Indian Ocean with comments on *the C. tokiensis* group of species (Teleostei: Gadiformes: Macrouridae). *Zootaxa* **2023** 5301(1), 137-150. (doi:10.11646/zootaxa.5301.1.7)
482. Iwamoto, T.; Graham, K.J. Two new Australian grenadiers of the *Coelorinchus fasciatus* species group (Macrouridae: Gadiformes: Teleostei). *Proc. Calif. Acad. Sci.* **2008** 59(5), 133-146.
483. Haneda, Y. The luminescence of some deep-sea fishes of the families Gadidae and Macrouridae. *Pac. Sci.* **1951** 5, 372–378.
484. Sazonov, Y.I.; Iwamoto, T. Grenadiers (Pisces, Gadiformes) of the Nazca and Sala y Gomez ridges, southeastern Pacific. *Proc. Calif. Acad. Sci.* **1992** 48(2), 27-95.
485. Arai, T.; Iwamoto, T. A new species of the macrourid fish genus *Coelorinchus* from off Tasmania, New Zealand, and the Falkland Islands. *Jpn. J. Ichthyol.* **1979** 26 (3), 238-246. (doi:10.11369/jji1950.26.238)
486. Iwamoto, T.; Anderson, E. Review of the grenadiers (Teleostei: Gadiformes) of southern Africa, with descriptions of four new species. *Ichthyol. Bull. J.L.B. Smith Inst. Ichthyol.* **1994** 61, 1-28.
487. Kelly, E.; Gerritsen, H.D. Monitoring the recovery of exploited deep-water species (EMFF 2014-2020 Marine Institute Report Series). Marine Institute.: Galway, Ireland, 2022; pp. 1-77.
488. Prokofiev, A.M.; Iwamoto, T. Revision of the grenadier genus *Coelorinchus* (Teleostei: Macrouridae) from the Mascarene Ridge, western Indian Ocean, with description of two new species. *Proc. Calif. Acad. Sci.* **2020** 66(9), 231-273.
489. Iwamoto, T.; Ungaro, N. A new grenadier (Gadiformes,Macrouridae) from the Mediterranean. *Cybium* 2002 26(1), 27-32.
490. Iwamoto, T.; Anderson, M.E. New species of *Caelorinchus* (Macrouridae, Gadiformes, Teleostei) from the Indian Ocean. *Copeia* **1999** 1999(4), 1079-1083.
491. Nakayama, N.; Endo, H. A new species of the grenadier genus *Coelorinchus* (Actinopterygii: Gadiformes: Macrouridae) from the Timor Sea, eastern Indian Ocean. *Ichthyol. Res.* **2018** 65, 12-20. (doi:10.1007/s10228-017-0585-4)
492. McMillan, P.; Iwamoto, T. Two new species of *Coelorinchus* (Teleostei, Gadiformes, Macrouridae) from the Tasman Sea. *Proc. Calif. Acad. Sci.* **2009** 60(4), 39-51.
493. Nakayama, N., Prokofiev, A.M.; Kawai, T. *Coelorinchus posteromaculatus* (Actinopterygii, Gadiformes, Macrouridae), a new species of grenadier from the eastern Indian Ocean. *Ichthyol. Res.* **2020** 67, 465-472. (doi:10.1007/s10228-020-00741-x)
494.
495. Nakayama, N.; Takaoka, H.; Miyamoto, K. First Record of the Grenadier *Coelorinchus sheni* (Actinopterygii: Gadiformes: Macrouridae) from Japan. *Species Divers.* **2018** 23(1), 121-127. (doi:10.12782/specdiv.23.121)
496. Prokofiev, A.M.; Iwamoto, T. A new species of the grenadier genus *Coelorinchus* (Gadiformes: Macrouridae) from the western Indian Ocean. *Zootaxa* **2022** 5194(2), 193-212. (doi:10.11646/zootaxa.5194.2.3)

497. McMillan, P.; Iwamoto, T. Descriptions of four species of grenadier fishes of the genera *Hymenocephalus* and *Hymenogadus* (Teleostei, Gadiformes, Macrouridae) from the New Zealand region and Tasman Sea, including two new species of *Hymenocephalus*. *Zootaxa* **2014** 3856(1), 117-134. (doi:10.11646/zootaxa.3856.1.5)
498. Nakayama, N.; Endo, H.; Schwarzhans, W. A new grenadier of the genus *Hymenocephalus* from Tosa Bay, southern Japan (Actinopterygii: Gadiformes: Macrouridae). *Ichthyol. Res.* **2015** 62(4): 504-511. (doi:10.1007/s10228-015-0464-9)
499. Gilbert, C.H.; Hubbs, C. L. (1917). Description of *Hymenocephalus tenuis*, a new macruroid fish from the Hawaiian Islands. *Proc. U. S. Natl. Mus.***1917** 54 (2231), 173–175. (doi:10.5479/si.00963801.54-2231.173)
500. Iwamoto, T. First Hawaiian record of the grenadier *Lepidorhynchus denticulatus* (Macrouridae: Gadiformes: Teleostei). *Proc. Calif. Acad. Sci.* **2005** 56(1/17), 23.
501. Sazonov, Y.I.; Shcherbachev, Y.N.; Iwamoto, T. The grenadier genus *Mataeocephalus* Berg, 1898 (Teleostei, Gadiformes, Macrouridae), with descriptions of two new species. *Proc. Calif. Acad. Sci.* **2003** 54 (17), 279-301.
502. Nakayama, N.; Endo, H. *Mesovagus*, a replacement name for the grenadier genus *Mesobius* Hubbs and Iwamoto 1977 (Actinopterygii: Gadiformes: Macrouridae), a junior homonym of *Mesobius* Chamberlin 1951 (Chilopoda: Lithobiomorpha: Lithobiidae). *Ichthyol. Res.* **2017** 64, 120-122. (doi:10.1007/s10228-016-0531-x)
503. Ruby, E.G.; Morin, J.G. Specificity of symbiosis between deep-sea fishes and psychrotrophic luminous bacteria. *Deep Sea Res.* **1978** 25(2), 161-167. (doi:10.1016/0146-6291(78)90003-6)
504. Iwamoto, T.; McMillan, P. A new grenadier, genus *Trachonurus*, from New Zealand and Australia (Pisces: Gadiformes: Macrouridae). *Mem. Mus. Victoria* **1997** 56(1), 255-259.
505. Iwamoto, T. *Trachonurus robinsi*, a new species of grenadier (Gadiformes, Macrouridae) from the Philippines. *Bull. Mar. Sci.* **1997** 60(3), 942-949.
506. Gilbert, C.H.; Cramer, F. Report on the fishes dredged in deep water near the Hawaiian Islands, with descriptions and figures of twenty-three new species. *Proc. U. S. Natl. Mus.* **1897** 19 (1114), 403-435. (doi:10.5479/si.00963801.19-1114.403)
507. Merrett, N.R. (2003). Preliminary guide to the identification of the early life history stages of bathygadid & macrourid fishes of the western central North Atlantic; National Oceanic and Atmospheric Administration: Florida, USA, 2003; pp. 1-31.
508. Günther, A.C.L.G. Report on the deep-sea fishes collected by HMS Challenger during the years 1873-76. *Rep. Sci. Res. Voy. HMS Challenger* **1887** 22, 1-268.
509. Cohen, D.M. Bioluminescence in the Gulf of Mexico anacanthine fish *Steindachneria argentea. Copeia* **1964** 1964(2), 406-409.
510. Marshall, N..; Cohen, D.M. Order Anacanthini (Gadiformes): characters and synopsis of families. *Mem. Sears Found. Mar. Res.* **1973** 1(6), 479-495.
511. Paulin, C.D.; Roberts, C.D. Review of the morid cods (Teleostei, Paracanthopterygii, Moridae) of New Caledonia, southwest Pacific Ocean, with description of a new species of *Gadella.* In Résultats des Campagnes MUSORSTOM 17; Séret, B., Ed.; Mémoires du Muséum national d'Histoire: Paris, France, 1997; pp. 17-41
512. Sazonov, Y.I.; Shcherbachev, Y.N. A review of the Indian Ocean species from the genus *Gadella* (Gadiformes, Moridae), with a description of two new species. *J. Ichthyol.* **2000** 40(Suppl. 1), S64-S73.
513. Paulin, C.D. Review of the morid genera *Gadella, Physiculus*, and *Salilota* (Teleostei: Gadiformes) with descriptions of seven new species. *N. Z. J. Zool.* **1989** 16(1), 93-133. (doi:10.1080/03014223.1989.10423706)
514. Long, D.J.; McCosker, J.E. A new species of the morid genus *Gadella* (Teleostei: Gadiformes) from the Galapagos Islands. *Ichthyol. Res.* **1998** 45(1), 1-5. (doi:10.1007/BF02678568)
515. Pires, A.M.; Carvalho-Filho, A.; Ferreira, R.C.; Viana, D.; Nunes, D.; Hazin, F.H. Review of the Brazilian species of *Physiculus* (Gadiformes: Moridae), with description of a new species from Saint Peter and Saint Paul Archipelago, equatorial Atlantic. *Zootaxa* **2019** 4671(1), 67-80. (doi:10.11646/zootaxa.4671.1.5)
516. Babu, K I.; Ho, H.C.; Mariyambi, P.C.; Sureshkumar, S. Two new species of the codling fish genus *Physiculus* from Lakshadweep, India (Gadiformes: Moridae). *Zootaxa* **2022** 5104(1), 111-124. (doi:10.11646/zootaxa.5104.1.6)
517. Tang, C.N. Description of a new codling species of *Physiculus* from Taiwan (Gadiformes: Moridae). *Zootaxa* **2021** 5052(1), 105-116. (doi:10.11646/zootaxa.5052.1.6)
518. Ho, H.C. A new genus and species of the cod fish family Moridae (Order Gadiiformes) from southwestern Taiwan. *Zootaxa* **2019** 4702(1), 32-40. (doi:10.11646/zootaxa.4702.1.8)
519. Gon, O.; Liao, Y.-C.; Kwang-Tsao, S. A new species of the cardinalfish genus *Jaydia* (Teleostei: Apogonidae) from the Philippines. *Zootaxa* **2015** 3980(2), 286-292. (doi:10.11646/zootaxa.4007.4.12)
520. Gon, O.; Allen, G.R. A new luminous cardinalfish of the genus *Apogon* (Perciformes: Apogonidae) from the western Pacific Ocean. *J.L.B. Smith Inst. Ichthyol. Spec. Publ.* **1998** 62, 1-9.

521. Haneda, Y.; Tsuji, F.I.; Sugiyama, N. Luminescent systems in apogonid fishes from the Philippines. *Science* **1969** 165 (3889),188–190. (doi:10.1126/science.165.3889.188)
522. Fraser, T.H.; Lachner, E.A. An unusual Indo-west Pacific cardinalfish of the genus *Apogon* (Teleostei: Apogonidae). *Proc. Biol. Soc. Wash.* **1984** 97(3), 632-636.
523. Gon, O.; Allen, G.R. Revision of the Indo-Pacific cardinalfish genus *Siphamia* (Perciformes: Apogonidae). *Zootaxa* **2012** 3294(1), 1-84. (doi:10.11646/zootaxa.3294.1.1)
524. Allen, G.R.; Erdmann, M.V. *Siphamia arnazae*, a new species of cardinalfish (Teleostei: Apogonidae) from Papua New Guinea. *J. Ocean Sci. Found.* **2019** 33, 1-8. (doi:10.5281/zenodo.3012186)
525. Fishelson, L.; Gon, O.; Goren, M.; Ben-David-Zaslow, R. The oral cavity and bioluminescent organs of the cardinal fish species *Siphamia permutata* and *S. cephalotes* (Perciformes, Apogonidae). *Mar. Biol.* **2005** 147, 603-609. (doi:10.1007/s00227-005-1613-x)
526. Haneda, Y. Observations on a luminous apogonid fish, *Siphamia versicolor* and on others of the same genus. *Sci. Rep. Yokosuka City Mus.* **1965** 11, 1–12.
527. Haneda, Y., Tsuji, F.I.; N. Sugiyama, N. Newly observed luminescence in apogonid fishes from the Philippines. *Sci. Rep. Yokosuka City Mus.* **1969** 15, 1–9.
528. Yoshida, T.; Motomura, H. A new cardinalfish, *Verulux solmaculata* (Perciformes: Apogonidae), from Papua New Guinea and Australia. *Ichthyol. Res.* **2016** 64(1), 64-70. (doi:10.1007/s10228-016-0539-2)
529. Fraser, T.H.; Prokofiev, A.M. A new genus and species of cardinalfish (Percomorpha, Apogonidae, Sphaeramiini) from the coastal waters of Vietnam: luminescent or not. *Zootaxa* **2016** 4144(2), 227-242. (doi:10.11646/zootaxa.4144.2.5)
530. Ramaiah, N.; Chandramohan, D. Occurrence of *Photobacterium leiognathi*, as the bait organ symbiont in frogfish *Antennarius hispidus*. *Indian J. Mar. Sci.* 1992 21, 210-211.
531. Ho, H.-C.; Kawai, T.; Amaoka, K. Records of deep-sea anglerfishes (Lophiiformes: Ceratioidei) from Indonesia, with descriptions of three new species. *Zootaxa* **2016** 4121(3), 267-294. (doi:10.11646/zootaxa.4121.3.3)
532. Ho, H.-C.; Shao, K.-T. Two new deep-sea anglerfishes (Oneirodidae and Gigantactidae) from Taiwan, with synopsis of Taiwanese ceratioids. *Zootaxa* **2019** 4702(1), 10-18. (doi:10.11646/zootaxa.4702.1.5)
533. Bertelsen, E.; Krefft, G.The ceratioid family Himantolophidae (Pisces, Lophiiformes). *Steenstrupia* **1988** 14(2), 9–89.
534. Beebe, W.; Crane, J. Eastern Pacific expeditions of the New York zoological society. XXXVII Deep-sea Ceratioid Fishes. *Zoologica N. Y.* **1947** 31, 151-181.
535. Rajeeshkumar, M.; Pietsch, T.W.; Saravanane, N. A new species of deep-sea anglerfish, genus *Himantolophus* (Lophiiformes: Himantolophidae) from the Andaman Sea, India. *Zootaxa* **2022** 5178(6), 589-594. (doi:10.11646/zootaxa.5178.6.6)
536. Stewart, A.L.; Pietsch, T.W. A new species of deep-sea anglerfish, genus *Himantolophus* (Lophiiformes: Himantolophidae) from the Western South Pacific, with comments on the validity of *H. pseudalbinares. Zootaxa* **2010** 2671, 53-60.
537. Haneda, Y. Observations on the luminescence of the deep sea luminous angler fish, *Himantolophus groenlandicus*. *Sci. Rept. Yokosuka City Mus.* **1968** 14, 1–6.
538. Pietsch, T.W.; Kenaley, C.P. A new species of deep-sea ceratioid anglerfish, genus *Himantolophus* (Lophiiformes: Himantolophidae), from southern waters of all three major oceans of the world. *Copeia* **2011** 2011(4), 490-496. (doi:10.2307/41416567)
539. Beebe, W. The depths of the sea. Strange life forms a mile below the surface. *Nat. Geogr. Mag.* **1932** 61(1), 65–88.
540. Bertelsen, E. Ceratioidei, Caulophrynidae, Melanocetidae, Himantolophidae, Diceratiidae, Oneirodidae, Thaumatichthyidae, Centrophrynidae, Ceratiidae, Gigantactinidae, and Linophrynidae]. In *Fishes of the North-eastern Atlantic and Mediterranean*, Vol. 3.; Whitehead, P.J.P.; Bauchot, M.-L., Hureau, J.-C., Nielsen, J.; Tortonese, E., Eds., United Nations Educational Scientific and Cultural Organization: Paris, France, 1986; pp. 1371–1414.
541. Hansen, K.; Herring, P.J. Dual bioluminescent systems in the anglerfish genus *Linophryne* (Pisces: Ceratioidea). *J. Zool.* **1977** 182(1), 103-124. (doi:10.1111/j.1469-7998.1977.tb04144.x)
542. Pietsch, T.W. Ergebnisse der Forschungsreisen des FFS "Walther Herwig" nach Südamerika. XIX. Systematics and distribution of ceratioid fishes of the genus *Dolopichthy*s (family Oneirodidae), with the description of a new species. *Arch. FischWiss.* **1972** 23(1), 1–28.
543. Waterman, T.H. Studies on deep-sea anglerfishes (Ceratioidea). I. An historical survey of our present state of knowledge. 11. Three new species. *Bull. Museum Comp. Zool. Harvard* **1939** 85, 65-94. (doi:10.1002/jmor.1050820202)

544. Herring, P.J.; O. Munk. The escal light gland of the deep-sea anglerfish *Haplophryne mollis* (Pisces: Ceratioidei), with observations on luminescence control. *J. Mar. Biol. Assoc. U. K.* **1994** 74, 747–763. (doi:10.1017/S0025315400090020)
545. Prokofiev, A.M. New and rare species of deepsea pelagic fishes of families Opisthoproctidae, Melanostomiidae, Oneirodidae, and Linophrynidae. *J. Ichthyol.* **2014** 54(6), 377-383. (doi:10.1134/S0032945214040092)
546. Prokofiev, A.M. New Species and New Records of Deepsea Anglerfish of the Family Oneirodidae. *J. Ichthyol.* **2014** 54 (8), 602-607. (doi:10.1134/S0032945214050075)
547. Rajeeshkumar, M.P.; Meera, K.M.; Hashim, M. A New Species of the Deep-Sea Ceratioid Anglerfish Genus Oneirodes (Lophiiformes: Oneirodidae) from the Western Indian Ocean. *Copeia* **2017** 105(1), 82-84.
548. Pietsch, T.W.; Sutton, T.T. A New Species of the Ceratioid Anglerfish Genus *Lasiognathus* Regan (Lophiiformes: Oneirodidae) from the Northern Gulf of Mexico. *Copeia* **2015** 103(2), 429-432.
549. Sutton, T.T.; Hulley, P.A.; Wienerroither, R.; Zaera-Perez, D.; Paxton, J.R. (2020). Identification guide to the mesopelagic fishes of the central and south-east Atlantic Ocean. Food and Agriculture Organization of the United Nations: Rome, Italy, 2020; pp. 1-346.
550. Edwards, A.S.; Herring, P.J. Observations on the comparative morphology and operation of the photogenic tissues of myctophid fishes. *Mar. Biol.* **1977** 41(1), 59-70. (doi:10.1007/BF00390582)
551. Wisner, R.L. The taxonomy and distribution of lanternfishes (family Myctophidae) in the eastern Pacific Ocean. *Rep. U.S. Nav. Ocean Res. Dev. Act.* **1976** 3, 1–229.
552. Hulley, P.A.; Duhamel, G. A review of the lanternfish genus *Bolinichthys* Paxton, 1972 (Myctophidae). *Cybium* **2009** 33(4), 259-304.
553. Mensinger, A.F.; Case, J.F. Luminescent properties of deep sea fish. *J. Exp. Mar. Biol. Ecol.* **1990** 144(1), 1-15. (doi:10.1016/0022-0981(90)90015-5)
554. Widder, E.A. Marine bioluminescence: why do so many animals in the open ocean make light? *Harbor Branch Oceanogr. Inst.* **2001** 1, 1–9.
555. Barnes, A.T.; Case, J.F. The luminescence of lanternfish (Myctophidae): spontaneous activity and responses to mechanical, electrical, and chemical stimulation. *J. Exp. Mar. Biol. Ecol.* **1974** 15(2), 203-221. (doi:10.1016/0022-0981(74)90046-X)
556. Kawaguchi, K.; Aioi, K. Myctophid fishes of the genus *Myctophum* (Myctophidae) in the Pacific and Indian Oceans. *J. Oceanogr.* **1972** 28, 161-175. (doi:10.1007/BF02108760)
557. Gilbert, C.H. Reports on the scientific results of the expedition to the tropical Pacific, in charge of alexander Agassiz, by the U.S. Fish Commission steamer "Albatross" from August 1899 to March 1900, Commander Jefferson T. Moser, U.S.N., commanding. X. The lantern fishes. *Mem. Mus. Comp. Zoològy* **1908** 26(6), 217-237.
558. Hulley, P.A. Myctophidae. In *Smiths' sea fishes*; Smith M.M.; Heemstra, P.C., Eds.; Springer-Verlag: Berlin, Germany, 1986; pp. 282-321.
559. Nafpaktitis, B.G. Systematics and distribution of lanternfishes of the genera *Lobianchia* and *Diaphus* (Myctophidae) in the Indian Ocean. *Nat. Hist. Mus. Los Angeles County, Sci. Bull.* **1978** 30, 1-92.
560. Prokofiev, A.M.; Emelyanova, O.R.; Orlov, A.M.; Orlova, S.Y. A New Species of *Diaphus* Associated with Seamounts of the Emperor Chain, North-Western Pacific Ocean (Teleostei: Myctophiformes: Myctophidae). *J. Mar. Sci. Eng.* **2022** 10(1), 65. (doi:10.3390/jmse10010065)
561. Becker, V.E.; Prut'ko, V.G. A new species of the genus *Diaphus* (Myctophidae) from the northeastern Indian Ocean. *J. Ichthyol.* **1984** 24 (6), 883-887.
562. Fowler, H.W. Descriptions of new fishes obtained from 1907 to 1910, chiefly in the Philippine Islands and adjacent seas. *Proc. Acad. Nat. Sci. Phila.* **1934** 85, 233-367.
563. Becker, V.E. Benthopelagic fishes of the genera *Idiolychnus* and *Diaphus* (Myctophidae) from the southeastern Pacific, with the description of two new species. *J. Ichthyol.* **1992** 32 (6), 3-10.
564. Flynn, A.J.; Pogonoski, J. J. Guide to Mesopelagic Fishes of the Southern Tasman Sea; CSIRO Marine and Atmospheric Research: Hobart, Australia, 2012; pp. 1-221.
565. Borsa, P.; Lagrange, D.; Millet, L.; Vourey, E. Lanternfish *Diaphus spp.* specimens of a DNA barcoding survey of the mesopelagic micronekton communities of the New Caledonian and Wal lis-and-Futuna EEZ. *Research Square* **2022** 14 Aug. (doi :10.21203/rs.3.rs-2973026/v2)
566. Tåning, A.V. Notes on scopelids from the Dana Expeditions. I. *Vidensk. Meddel. Naturhist. Foren. Kjøbenhavn* **1932** 94, 125-146.
567. Kulikova, E.B. On the lantern fishes of the genus *Diaphus* (Scopelidae) from the western Pacific. *Tr. Inst. Okeanol. Im. P. P. Shirshova, Akad. Nauk SSSR.* **1961** 43, 5-39.
568. Baguet, F. Excitation and control of isolated photophores of luminous fishes. *Prog. Neurobiol.* **1975** 5, 97-125. (doi:10.1016/0301-0082(75)90016-7)

569. Nafpaktitis, B.G.; Robertson, D.A.; Paxton, J.R. Four new species of the lanternfish genus *Diaphus* (Myctophidae) from the Indo-Pacific. *N. Z. J. Mar. Freshwater Res.* **1995** 29 (3), 335-344. (doi:10.1080/00288330.1995.9516668)
570. Tsuji, F.I.; Haneda, Y. Luminescent system in a myctophid fish, *Diaphus elucens* Brauer. *Nature* **1971** 233(5322), 623-624. (doi:10.1038/233623a0)
571. Kawaguchi, K.; Nafpaktitis, B.G. A new lanternfish, *Diaphus kuroshio* (family Myctophidae), from the Kuroshio waters off Japan. *Jpn. J. Ichthyol.* **1978** 25(2), 89-91. (doi:10.11369/jji1950.25.89)
572. Becker, V.E.; Shcherbachev, Y.N. Benthopelagic species of the families Neoscopelidae and Myctophidae from the Indian Ocean, with a description of a new species of *Diaphus*. *J. Ichthyol.* **1990** 30 (5), 845-855.
573. Nafpaktitis, B.G. Taxonomy and distribution of the lanternfishes, genera *Lobianchia* and *Diaphus*, in the North Atlantic. *Dana Rep.* **1968** 73, 1-131.
574. Gjøsaeter, J. *Diaphus pallidus* sp. n., a new lanternfish from the Gulf of Aden (Pisces, Myctophidae). *Zool. Scr.* **1989** 18(4), 537-539. (doi:10.1111/j.1463-6409.1989.tb00147.x)
575. Denton, E.J.; Herring, P.J.; Widder, E.A.; Latz, M.F.; Case, J.F. The roles of filters in the photophores of oceanic animals and their relation to vision in the oceanic environment. *Proc. Roy. Soc. B.* **1985** 225(1238), 63-97. (doi:10.1098/rspb.1985.0051)
576. Bourret, P. Poissons Téléostéens: Gonostomatidae, Sternoptychidae, et Myctophidae (MUSORSTOM II). In *Résultats des Campagnes MUSORSTOM I et II Philippines (1976, 1980) (Tome 2)*; Forest, J., Ed. ; Mémoires du Muséum national d'Histoire naturelle (Série A, Zoologie. 133), Paris, France, 1985; pp. 55-82.
577. Nafpaktitis, B.G. A new record and a new species of lanternfish, genus *Diaphus* (family Myctophidae), from the North Atlantic Ocean. *Contrib. Sci. Los Angel. Calif.* **1974** 254, 1-6.
578. Gilbert, C.H. The lanternfishes of Japan. *Mem. Carnegie Mus.* **1913** 6(2), 67-107.
579. Paitio, J.; Yano, D.; Muneyama, E.; Takei, S; Asada, H.; Iwasaka, M.; Oba, Y. Reflector of the body photophore in lanternfish is mechanistically tuned to project the biochemical emission in photocytes for counterillumination. *Biochem. Biophys. Res. Commun.* **2020** 521(4), 821-826. (doi:10.1016/j.bbrc.2019.10.197)
580. Duchatelet, L.; Hermans, C.; Duhamel, G.; Cherel, Y.; Guinet, C.; Mallefet, J. Coelenterazine detection in five myctophid species from the Kerguelen Plateau. In *Proceedings of the Second Kerguelen Plateau Symposium on Marine Ecosystems and Fisheries*, Hobart, Australia, 15 November 2017.
581. Nafpaktitis, B.G.; Paxton, J.R. *Idiolychnus*, a new genus of Myctophidae based on *Diaphus urolampus*. *Copeia* **1978** 1978, 492-497.
582. Maul, G.E. A new subspecies of *Lampadena urophaos* Paxton 1963 from the Atlantic Ocean. Bocagiana. *Bol. Mus. Munic. Funchal* **1969** 22, 1-8.
583. Zahuranec, B.J. Zoogeography and systematics of the lanternfishes of the genus *Nannobrachium* (Myctophidae: Lampanictini). *Smithson. Contrib. Zool.* **2000** 607, 1-69. (doi:10.5479/si.00810282.607)
584. Beebe, W. Eastern Pacific expeditions of the New York Zoological Society. XXXIII. Pacific Myctophidae (fishes). *Zool. N.Y.* **1944** 29, 59-95.
585. O'Day, W.T. The histology and fine structure of some bioluminescent organs in deep-sea fishes. PhD thesis, University of Southern California, Berkeley, USA, September 1972.
586. Day, F. The fishes of India; being a natural history of the fishes known to inhabit the seas and fresh waters of India, Burma, and Ceylon. Bernard Quaritch: London, UK, 1877; pp. 369-552.
587. Becker, V.E.; Borodulina, O.D. "*Myctophum asperum*" species-group with description of a new species, and *Myctophum selenops* Tåning (Myctophidae, Osteichthyes). Taxonomy and distribution. *Tr. Inst. Okeanol. Im. P. P. Shirshova, Akad. Nauk SSSR* **1978** 111, 108-128.
588. Tsarin, S.A. Description of a new species in the species group "*Myctophum asperum*" (Myctophidae) with comments on this group. *J. Ichthyol.* **1993** 33(4), 93-98.
589. Nicol, J.A.C.J. Spectral composition of the light of the lanternfish, *Myctophum punctatum*. *J. Mar. Biol. Assoc. U. K.* **1960** 39(1), 27-32. (doi:10.1017/S0025315400013072)
590. Coad, B.W. *Encyclopedia of Canadian fishes.* Canadian Museum of Nature and Canadian Sportfishing Productions: Singapore, Republic of Singapore, 1995; pp. 1-928.
591. Anctil, M. Stimulation of bioluminescence in lanternfishes (Myctophidae) II. *Can. J. Zool.* **1972** 50(2), 233-237. (doi:10.1139/z72-034)
592. Hubbs, C.L.; Wisner, R.L. *Parvilux*, a new genus of myctophid fishes from the northeastern Pacific, with two new species. *Zool. Med. Leiden* **1964** 39, 445-463.
593. Prokofiev, A.M. Two new species of *Protomyctophum* (Pisces, Teleostei, Myctophidae) from the Southern Ocean. *Beaufortia* **2004** 54(4), 67-74.

594. Anctil, M.; Gruchy, C.G. Stimulation and photography of bioluminescence in lanternfishes (Myctophidae). *J. Fish. Res. Board Can.* **1970** 27(4), 826-829. (doi:10.1139/f70-088)
595. Gago, F.J.; Ricord, R.C. *Symbolophorus reversus*: A New Species of Lanternfish from the Eastern Pacific (Myctophiformes: Myctophidae). *Copeia* **2005** 2005(1), 138-145.
596. Maul G.E. Nota sobra as duas espécies de género *Neoscopelus*. *Bol. Mus. Munic. Funchal* **1951** 5(13), 56-63.
597. Arai, R. A new iniomous fish of the genus *Neoscopelus* from Suruga Bay, Japan. *Bull. Natn. Sci. Mus. Tokyo* **1969** 12(3), 465-471.
598. Poulsen, J.Y.; Thorkildsen, S.; Arboe, N.H. Identification keys to halosaurs and notacanthids (Notacanthiformes, Elopomorpha) in the subarctic Atlantic Ocean including three new distributional records and multiple molecular OTUs of *Notacanthus cf. chemnitzii*. *Mar. Biodivers.* **2018** 48, 1009-1025. (doi:10.1007/s12526-017-0762-8)
599. Garman, S. The fishes. *Mem. Mus. Comp. Zool. Harvard* **1899** 24, 1-43 1.
600. McAllister, D. E. The significance of ventral bioluminescence in fishes. *J. Fish. Res. Board Can.* **1967** 24(3), 537-554. (doi: 10.1139/f67-047)
601. Wood-Mason, J.; Alcock, A. Natural history notes from H.M. Indian marine survey steamer 'Investigator', Commander R.F. Hoskyn, R.N., commanding. Series II, No. 1. On the results of deep-sea dredging during the season 1890-1891. *Ann. Mag. Nat. Hist.* **1891 8(6)**, 268-284.
602. Nielsen, J.G.; Bertelsen, E. The gulper-eel family Saccopharyngidae (Pisces, Anguilliformes). *Steenstrupia* **1985** 11(6), 157-206.
603. Tighe, K. A.; Nielsen, J.G. *Saccopharynx berteli*, a new gulper eel from the Pacific Ocean (Teleostei, Saccopharyngidae). *Ichthyol. Res.* **2000** 47(1), 39-41. (doi:10.1007/BF02674311)
604. Beebe, W. Nineteen new species and four post-larval deep-sea fish. *Zool. N. Y.* **1932** 13, 47-107.
605. Melo, M.R.S. A revision of the genus *Pseudoscopelus* Lütken (Chiasmodontidae: Acanthomorphata) with descriptions of three new species. *Zootaxa* **2010** 2710, 1-78. (doi:10.11646/zootaxa.2710.1.1)
606. Poey y Aloy, F. *Memorias sobre la historia natural de la isla de Cuba, acompañadas de sumarios latinos y extractos en francés.* Imprenta de Barcina: Havana, Cuba, 1851; pp. 311–312.
607. Grey, M. Gonostomatidae. In *Fishes of the Western North Atlantic*; Bigelow, H.B.; Cohen, D.M.; Dick, M.M.; Gibbs, R.H.Jr.; Grey, M.; Morrow, J.E.Jr.; Schultz, L.P.; Eds.; Memoir I Sear Foundation for Marine Research (Part 4): New Haven, USA, 1964; pp. 78–240.
608. Claes, J.M.; Mallefet, J. Hormonal control of luminescence from lantern shark (*Etmopterus spinax*) photophores. *J. Exp. Biol.* **2009** 212(22), 3684-3692. (doi:10.1242/jeb.034363)
609. Miya, M. *Cyclothone kobayashii,* a new gonostomatid fish (Teleostei: Stomiiformes) from the Southern Ocean, with notes on its ecology. *Copeia* **1994** 1994, 191-204.
610. Ozawa, T.; Oda, K.; Ida, T. Systematics and distribution of the *Diplophos taenia* species complex (Gonostomatidae), with a description of a new species. *Jpn. J. Ichthyol.* **1990** 37(2), 98-115. (doi:10.11369/jji1950.37.98)
611. David, P.M. (1970). Vertical distribution of zooplankton at 18N, 25W - RRS" Discovery" Cruise 30, 15 October-15 December 1969. National Institute of Oceanography: Wormley, UK, 1970; pp. 1-30.
612. Koeda, K.; Ho, H.C. A new Portholefish of the genus *Diplophos* (Stomiiformes: Gonostomatidae) from the western Pacific Ocean. *Zootaxa* **2019** 4702(1), 107-113. (doi:10.11646/ZOOTAXA.4702.1.15)
613. Ozawa, T.; Katayama, H. Early ontogeny of a South Pacific gonostomatid fish *Sigmops longipinnis*. *Ichthyol. Res.* **2003** 50, 195-197. (doi:10.1007/s10228-002-0151-5)
614. Mukhacheva, V.A. A review of the genus *Ichthyococcus* Bonaparte (Photochthyidae). *J. Ichthyol.* **1980** 20(6), 1-14.
615. Baguet, F.; Maréchal, G. Bioluminescence of bathypelagic fish from the Strait of Messina. *Comp. Biochem. Physiol. C Toxicol. Pharmacol.* **1976** 53(2), 75-82. (doi:10.1016/0306-4492(76)90057-5)
616. Parin, N.V.; Borodulina, O.D. Survey of the genus *Polymetme* (Photichthyidae) with a description of two new species. *J. Ichthyol.* **1990** 30(6): 108-121.
617. Mallefet, J.; Shimomura, O. Presence of coelenterazine in mesopelagic fishes from the Strait of Messina. *Mar. Biol.* **1995** 124, 381-385. (doi:10.1007/BF00363911)
618. Ahlstrom, E.; Counts, R. Development and distribution of *Vinciguerria lucetia* and related species in the Eastern Pacific. *Fish. Bull.* **1958** 139(58), 363–416.
619. Johnson, R.K.; Feltes, R.M. A new species of *Vinciguerria (*Salmoniformes: Photichthyidae) from the Red Sea and Gulf of Aqaba, with comments on the depauperacy of the Red Sea mesopelagic fish fauna. *Fieldiana Zool.* **1984** 22, 1-35. (doi:10.5962/bhl.title.2849)
620. Evseenko, S.A.; Suntsov, A.V. Early Stages of Development of Rare Deepwater Fishes from Notal Waters of the Southwestern Pacific. *J. Ichthyol.* **1995** 35(8), 111-122.

621. Ahlstrom, E.H.; Moser, H.G. A new gonostomatid fish from the tropical eastern Pacific. *Copeia* **1969** 1969(3), 493-500.
622. Prokofiev, A.M. *Argyripnus boreopacificus* sp. nova, a new species of hatchetfishes (Sternoptychidae) from the subtropical Northwestern Pacific. *J. Ichthyol.* **2017** 57(2), 325-328. (doi:10.1134/S0032945217020151)
623. Quéro, J.C. ; Spitz, J. ; Vayne, J.J. *Argyripnus hulleyi*: une nouvelle espèce de Sternoptychidae (Stomiiformes) de l'île de la Réunion (France, océan Indien). *Cybium* **2009** 33(1), 39-43.
624. Schultz, L.P. Review of the fishes of the genera *Polyipnu*s and *Argyropelecus* (Family Sternoptichidae), with descriptions of three new species. *Proc. U. S. Natl. Mus.* **1938** 86(3047), 135–155. (doi:10.5479/si.00963801.86-3047.135)
625. Kro̎nstro̎m, J.; Holmgren, S.; Baguet, F.; Salpietro, L.; Mallefet, J. Nitric oxide in control of luminescence from hatchetfish (*Argyropelecus hemigymnus*) photophores. *J. Exp. Biol.* **2005** 208(15), 2951-2961. (doi:10.1242/jeb.01712)
626. Grey, M. A preliminary review of the family Gonostomatidae, with a key to the genera and the description of a new species from the tropical Pacific. *Bull. Mus. Comp. Zool. Harvard* **1960** 122, 57-125.
627. Ahlstrom, E.H.; Richards, W.J.; Weitzman, S.H. Families Gonostomatidae, Sternoptychidae, and associated stomiiform groups: development and relationships. Proceedings from *An International Symposium Dedicated to the Memory of Elbert Halvor Ahlstrom*, Lawrence, USA, 15-18 august 1983.
628. Parin, N.V.; Kobyliansky, S.G. Review of the genus *Maurolicus* (Sternoptychidae, Stomiiformes), with re-establishing validity of five species considered junior synonyms of *M. muelleri* and descriptions of nine new species. *Tr. Inst. Okeanol. im. P. P. Shirshova, Akad. Nauk SSSR* **1993** 128, 69-107.
629. Baguet, F.; Christophe, B. Adrenergic stimulation of isolated photophores of *Maurolicus muelleri*. *Comp. Biochem. Physiol. C Toxicol. Pharmacol.* **1993** 75(1), 79-84. (doi:10.1016/0742-8413(83)90014-2)
630. Vourey, E.; Dupoux, C.; Harold, A.S. A new species of *Polyipnus* (Stomiiformes: Sternoptychidae) from the Western South Pacific. *Zootaxa* **2017** 4263(3), 567-577. (doi:10.11646/zootaxa.4263.3.8)
631. Haneda, Y. Some luminous fishes from the genera *Yarrella* and *Polyipnus*. *Pac. Sci.* **1952** 4,13–16.
632. Liao, Y.C.; Shao, K.T. Redescription of hachetfish, *Thorophos nexilis (*Myers, 1932)(Pisces: Stormiiformes: Sternoptychidae: Maurolicinae) from the Philippines with a key to the genus *Thorophos* Bruun, 1932. *J. Nat. Taiwan Mus.* **2008** 61(1), 17-23.
633. Parin, N.V.; Borodulina, O.D. Phylogeny, systematics, and zoogeography of the mesopelagic genus *Astronesthes* (Astronesthidae, Stomiiformes). *J. Ichthyol.* **2003** 43, 581- 601.
634. Liao, Y.-C.; Chen, L.-S.; Shao, K.-T. Review of the Astronesthid fishes (Stomiiformes: Stomiidae: Astronesthinae) from Taiwan with a description of one new species. *Zool. Stud.* **2006** 45(4), 517-528.
635. Nakayama, N.; Ohashi, S.; Tanaka, F. A new species of *Astronesthes* (Actinopterygii, Stomiidae, Astronesthinae) snaggletooth from the tropical western Pacific, with comments on the occurrence of *Astronesthes luetkeni* Regan and Trewavas in Japanese waters. *Ichthyol. Res.* **2021** 69, 8-89. (doi:10.1007/s10228-021-00813-6)
636. Barnett, M.A.; Gibbs R.H.Jr. Four new stomiatoid fishes of the genus *Bathophilus* with a revised key to the species of *Bathophilus*. *Copeia* **1968** 1968(4), 826-832.
637. Morrow, J.E.,Jr. Astronesthidae. In *Fishes of the Western North Atlantic*; Bigelow, H.B.; Cohen, D.M.; Dick, M.M.; Gibbs, R.H.Jr.; Grey, M.; Morrow, J.E.Jr.; Schultz, L.P.; Eds.; Memoir I Sear Foundation for Marine Research (Part 4): New Haven, USA, 1964; pp. 311–350.
638. Morrow, J.E.,Jr. Chauliodontidae. In *Fishes of the Western North Atlantic*; Bigelow, H.B.; Cohen, D.M.; Dick, M.M.; Gibbs, R.H.Jr.; Grey, M.; Morrow, J.E.Jr.; Schultz, L.P.; Eds.; Memoir I Sear Foundation for Marine Research (Part 4): New Haven, USA, 1964; pp. 274–289.
639. Morrow, J.E.Jr. 1961. Taxonomy of the deep sea fishes of the genus *Chauliodus*. *Bull. Mus. Comp. Zool.* **1961** 125, 249-294.
640. Mallefet, J.; Duchatelet, L.; Hermans, C.; Baguet, F. Luminescence control of Stomiidae photophores. *Acta Histochem.* **2019** 121(1), 7-15. (doi:10.1016/j.acthis.2018.10.001)
641. Novikova, N.S. A new species of the genus *Chauliodus* (Pisces, Chauliodontidae) from the southeastern Pacific. *J. Ichthyol.* **1972** 12(1), 41-48.
642. Beebe, W.; Crane, J. Deep-sea fishes of the Bermuda oceanographic expeditions: family Melanostomiatidae. *Zool. N. Y.* **1939** 24, 65-238.
643. Prokofiev, A.M. Three New Species of *Eustomias* (Melanostomiidae) From the Southern Subtropical Waters with the Description of a New Subgenus. *J. Ichthyol.* **2018** 58(1), 1-7. (doi:10.1134/S0032945218010113)
644. Nicol, J.A.C. (1969). 7 Bioluminescence. In *Fish physiology*; Hoar, W.S.; Randall, D.J.; Academic Press: Cambridge, USA, 1969; pp. 355-400

645. Koeda, K.; Ho, H.C. Review of the genus *Eustomias* (Stomiiformes: Stomiidae: Melanostomiinae) of Taiwan, with descriptions of three new species. *Zootaxa* **2019** 4702(1), 94-106. (doi:10.11646/zootaxa.4702.1.14)
646. Prokofiev, A.M. On Some Little-known Melanostomiidae with the Description of a New Species of *Eustomias*. *J. Ichthyol.* **2020** 60(3), 345-354. (doi:10.1134/S0032945220030121)
647. Prokofiev, A.M. New *Eustomias* Species (Melanostomiidae) from New Caledonia. *J. Ichthyol.* **2020** 60(2): 331-334. (doi:10.1134/S0032945220020162)
648. Prokofiev, A.M.; Orlov, A.M. *Eustomias securicula* sp. nov.—the Second Representative of the Subgenus *Biradiostomias* (Melanostomiidae) in the Pacific Ocean. *J. Ichthyol.* **2022** 62(2), 316-319. (doi:10.1134/S0032945222020151)
649. Prokofiev, A.M. New Species of *Eustomias* from the Waters of Madagascar (Melanostomiidae). *J. Ichthyol.* **2019** 59(2), 263-265. (doi:10.1134/S0032945219020176)
650. Prokofiev, A.M. *Grammatostomias ovatus* sp. nova (Melanostomiidae). *J. Ichthyol.* **2014** 54(6), 426-427. (doi:10.1134/s0032945214040109)
651. Brauer, A. Die Tiefsee-Fische. I. Systematischer Teil. In *Wissenschaftl. Ergebnisse der deutschen Tiefsee-Expedition "Valdivia," 1898-99*; Chun, C., Ed., Gustav fischer, Jena, Germany, 1906, pp. 1-432.
652. Beebe, W. Deep-sea fishes of the Bermuda Oceanographic expeditions. Family Idiacanthidae. *Zool. N. Y.* **1934** 16, 149-241.
653. Morrow, J.E.Jr.; Gibbs, R.H.Jr., Melanostomiatidae. In *Fishes of the Western North Atlantic*; Bigelow, H.B.; Cohen, D.M.; Dick, M.M.; Gibbs, R.H.Jr.; Grey, M.; Morrow, J.E.Jr.; Schultz, L.P.; Eds.; Memoir I Sear Foundation for Marine Research (Part 4): New Haven, USA, 1964; pp. 351–511.
654. Kenaley, C.P. Revision of the stoplight loosejaw genus *Malacosteus* (Teleostei: Stomiidae: Malacosteinae), with description of a new species from the temperate Southern Hemisphere and Indian Ocean. *Copeia* **2007** 2007(4), 886-900.
655. Parin, N.V.; Pokhil'skaya, G.N. On the taxonomy and distribution of the mesopelagic fish genus *Melanostomias* Brauer (Melanostomiatidae, Osteichthyes). *Tr. Inst. Okeanol. Im. P. P. Shirshova, Akad. Nauk SSSR* **1978** 111, 61-86.
656. Norman, J.R. Oceanic fishes and flatfishes collected in 1925-1927. *Discovery Rep.* **1930** 2, 261-369.
657. Milkova, V. Range records for ten species of stomiiform, aulopiform, and myctophiform fishes in British Columbia, Canada. *Northwest. Nat.* **2016** 97(2), 113-123. (doi:10.1898/NWN15-11.1)
658. Herring, P J.; Cope, C. Red bioluminescence in fishes: on the suborbital photophores of *Malacosteus*, *Pachystomias* and *Aristostomias*. *Mar. Biol.* **2005** 148(2), 383-394. (doi:10.1007/s00227-005-0085-3)
659. Prokofiev, A.M.; Frable, B.W. A new species of *Photonectes* from the Pacific Ocean off Oahu, Hawaii with a revised identification key for the genus (Teleostei: Stomiidae: Melanostomiinae). *Ichthyol. Herpetol.* 2021 109(3), 720-727. (doi:10.1643/i2020148)
660. Prokofiev, A.M.; Klepadlo, C. Two new species of *Photonectes* with blue luminous tissue on body, and a re-examination of *P. mirabilis* (Teleostei: Stomiidae). *Zootaxa* **2019** 4590(2), 270-282. (doi:10.11646/zootaxa.4590.2.4)
661. Prokofiev, A.M. Materials for revision of the genus *Photonectes* (Melanostomiidae). *J. Ichthyol.* **2019** 59, 449-476. (doi:10.1134/S003294521904012X)
662. Kenaley, C.P.; Hartel, K.E. A revision of Atlantic species of *Photostomias* (Teleostei: Stomiidae: Malacosteinae), with a description of a new species. *Ichthyol. Res.* **2005** 52, 251-263. (doi:10.1007/s10228-005-0281-7)
663. Kenaley, C.P. Revision of Indo-Pacific species of the loosejaw dragonfish genus *Photostomias* (Teleostei: Stomiidae: Malacosteinae). *Copeia* **2009** 2009(1), 175-189.
664. Gibbs, R.H.Jr. Taxonomy, sexual dimorphism, vertical distribution, and evolutionary zoogeography of the bathypelagic fish genus *Stomias* (family Stomiatidae). *Smithson. Contrib. Zool.* **1969** 31, 1–25.
665. Bolin, R L. A new stomiatoid fish from California. *Copeia* **1939** 1939(1), 39-41.
666. Johnson, G.D.; Rosenblatt, R.H. Mechanisms of light organ occlusion in flashlight fishes, family Anomalopidae (Teleostei: Beryciformes), and the evolution of the group. *Zool. J. Linn. Soc.* **1988** 94(1), 65-96. (doi:10.1111/j.1096-3642.1988.tb00882.x)
667. Rosenblatt, R.H.; Johnson, G.D. *Parmops coruscans*, a new genus and species of flashlight fish (Beryciformes: Anomalopidae) from the South Pacific. *Proc. Biol. Soc. Wash.* **1991** 104(2), 328-334.
668. Johnson, G.D.; Seeto, J.; Rosenblatt, R.H. *Parmops echinatus*, a new species of flashlight fish (Beryciformes: Anomalopidae) from Fiji. *Proc. Biol. Soc. Wash.* **2001** 114 (2), 497-500.
669. Hellinger, J.; Jägers, P.; Spoida, K.; Weiss, L.C.; Mark, M.D.; Herlitze, S. Analysis of the Territorial Aggressive Behavior of the Bioluminescent Flashlight Fish *Photoblepharon steinitzi* in the Red Sea. *Front. Mar. Sci.* **2020** 7, 78. (doi:10.3389/fmars.2020.00078)

670. Ho, H.-C.; Johnson, G.D. *Protoblepharon mccoskeri*, a new flashlight fish from eastern Taiwan (Teleostei: Anomalopidae). *Zootaxa* **2012** 3479, 77-87. (doi:10.11646/zootaxa.3479.1.5)
671. Baldwin, C.C.; Johnson, G.D.; Paxton, J.R. *Protoblepharon rosenblatti*, a new genus and species of flashlight fish (Beryciformes: Anomalopidae) from the tropical South Pacific, with comments on anomalopid phylogeny. *Proc. Biol. Soc. Wash.* **1997** 110 (3), 373-383.
672. Su, Y.; Lin, H.-C.; Ho, H.-C. A new cryptic species of the pineapple fish genus *Monocentris* (Family Monocentridae) from the western Pacific Ocean, with redescription of *M. japonica* (Houttuyn, 1782). *Zootaxa* **2022** 5189(1), 180-203. (doi:10.11646/zootaxa.5189.1.18)
673. Ghedotti, M.J.; DeKay, H.M.; Maile, A.J.; Smith, W.L.; Davis, M.P. (2021). Anatomy and evolution of bioluminescent organs in the slimeheads (Teleostei: Trachichthyidae). *J. Morphol.* **2021** 282(6), 820-832. (doi:10.1002/jmor.21349).
674. Haneda, Y. Observations on luminescence in the deep sea fish, *Paratrachichthys prosthemius*. *Sci. Rep. Yokosuka City Mus.* **1957** 2, 15-23.
675. Suntsov, A.V.; Widder, E.A.; Sutton, T.T. Bioluminescence in larval fishes. In *Fish Larval Physiology*, Finn, R.N.; Kapoor, B.G., Eds.; Univ. Bergen Press: Bergen, Norway, 2008; pp. 51–88.
676. Satterlie, R.A.; Anderson, P.A.V.; Case, J.F. Colonial coordination in anthozoans: Pennatulacea. *Mar. Behav. Physiol.* **1980** 7(1), 25 – 46. (doi:10.1080/10236248009386969)
677.)
